# Combined experience of six independent laboratories attempting to create an Ewing sarcoma mouse model

**DOI:** 10.18632/oncotarget.9388

**Published:** 2016-05-15

**Authors:** Tsion Zewdu Minas, Didier Surdez, Tahereh Javaheri, Miwa Tanaka, Michelle Howarth, Hong-Jun Kang, Jenny Han, Zhi-Yan Han, Barbara Sax, Barbara E. Kream, Sung-Hyeok Hong, Haydar Çelik, Franck Tirode, Jan Tuckermann, Jeffrey A. Toretsky, Lukas Kenner, Heinrich Kovar, Sean Lee, E. Alejandro Sweet-Cordero, Takuro Nakamura, Richard Moriggl, Olivier Delattre, Aykut Üren

**Affiliations:** ^1^ Department of Oncology, Georgetown University Medical Center, Washington, DC, United States of America; ^2^ Genetics and Biology of Cancers Unit, Institut Curie Research Center, PSL Research University, Île-de-France, Paris, France; ^3^ INSERM U830, Institut Curie Research Center, Île-de-France, Paris, France; ^4^ Ludwig Boltzmann Institute for Cancer Research, Vienna, Austria; ^5^ Division of Carcinogenesis, The Cancer Institute, Japanese Foundation for Cancer Research, Tokyo, Japan; ^6^ Division of Hematology and Oncology, Department of Pediatrics, Stanford University School of Medicine, Stanford, CA, United States of America; ^7^ Department of Pathology and Laboratory Medicine, Tulane University School of Medicine, New Orleans, LA, United States of America; ^8^ Department of Medicine, and Genetics and Genome Sciences, University of Connecticut Health Science Center, Farmington, CT, United States of America; ^9^ Institute of Comparative Molecular Endocrinology (CME), University of Ulm, Ulm, Germany; ^10^ Clinical Institute of Pathology, Medical University of Vienna, Vienna, Austria; ^11^ Institute of Animal Breeding and Genetics, University of Veterinary Medicine, Vienna, Austria; ^12^ Department of Pediatrics, Medical University of Vienna, Vienna, Austria; ^13^ Children´s Cancer Research Institute, St. Anna Kinderkrebsforschung, Vienna, Austria; ^14^ Unité de génétique somatique, Institut Curie, Île-de-France, Paris, France; ^15^ Department of Pathology of Laboratory Animals (UPLA), University of Veterinary Medicine, Vienna, Austria; ^16^ Medical University of Vienna, Vienna, Austria

**Keywords:** Ewing sarcoma, EWS-FLI1, EWS-FLI1 driven transgenic mouse model

## Abstract

Ewing sarcoma (ES) involves a tumor-specific chromosomal translocation that produces the EWS-FLI1 protein, which is required for the growth of ES cells both *in vitro* and *in vivo*. However, an EWS-FLI1-driven transgenic mouse model is not currently available. Here, we present data from six independent laboratories seeking an alternative approach to express EWS-FLI1 in different murine tissues. We used the *Runx2*, *Col1a2.3*, *Col1a3.6*, *Prx1*, *CAG*, *Nse*, *NEFL*, *Dermo1*, *P0*, *Sox9* and *Osterix* promoters to target *EWS-FLI1* or *Cre* expression. Additional approaches included the induction of an endogenous chromosomal translocation, *in utero* knock-in, and the injection of *Cre*-expressing adenovirus to induce *EWS-FLI1* expression locally in multiple lineages. Most models resulted in embryonic lethality or developmental defects. EWS-FLI1-induced apoptosis, promoter leakiness, the lack of potential cofactors, and the difficulty of expressing EWS-FLI1 in specific sites were considered the primary reasons for the failed attempts to create a transgenic mouse model of ES.

## INTRODUCTION

Ewing sarcoma (ES) is a highly malignant tumor of bone and soft tissue that occurs in children, adolescents, and young adults. Tumors often grow in close proximity to bone but can occur as soft tissue masses [1–3]. ES cases show a balanced chromosomal translocation [4] that joins the *EWS* gene (EWing Sarcoma) located on chromosome 22 to an ETS family gene, which is most commonly either *FLI1* (Friend Leukemia Insertion) located on chromosome 11, t(11;22) or *ERG* located on chromosome 21, t(21;22). The resulting fusion protein is termed EWS-FLI1 or EWS-ERG, respectively. Other infrequent variant fusion proteins are the products of ES translocations and are absent in non-tumor cells. FLI1 is an ETS family transcription factor with a conserved DNA binding domain. The carboxy terminal half of FLI1 contained in the EWS-FLI1 fusion protein retains its DNA binding domain. Therefore, EWS-FLI1 binds to DNA through the conserved ETS binding domain. However, the EWS-FLI1 fusion protein functions by a different mechanism than either EWS or FLI1 [5]. EWS-FLI1 is required to maintain the growth of ES cell lines, and when the expression level of EWS-FLI1 is reduced by alternative mechanisms, ES cell lines die in culture and xenografts in nude mice regress [6–13].

While the oncogenic activity of EWS-FLI1 is clear, the cell of origin for ES has been confounding due to the cytotoxic effects of expressing EWS-FLI1 in most primary cell types [14–16]. Previous studies have identified three primary cell types that are permissive for EWS-FLI1 expression and thus represent prime candidates for the elusive tumor cell of origin: (i) mesenchymal stem cells (MSCs) [17–19], (ii) neural crest stem cells [20], and (iii) embryonic osteochondrogenic progenitor cells [21].

Transgenic mouse models have been successfully developed for neoplasms with tumor-specific chromosomal translocations, including alveolar rhabdomyosarcoma, synovial sarcoma, myxoid liposarcomas, and clear cell sarcomas [22–27]. However, the same success has not been achieved in ES. When EWS-FLI1 was expressed ubiquitously under the native *EWS* promoter, either *in utero* or in adult mice, it resulted in lethality [16]. Because EWS-FLI1 induces apoptosis in mouse embryonic fibroblasts *in vitro*, the embryonic lethality resulting from broad transgenic expression was not surprising. When the expression of EWS-FLI1 was restricted to specific cell types, the animals survived but did not develop ES. EWS-FLI1 expression under the control of the *Prx1* promoter resulted in developmental malformations in the limbs, but not tumor formation [28]. When these animals were crossed with p53 null mice, EWS-FLI1 expression accelerated the p53 null-induced formation of osteosarcoma and shifted the tumor histology from osteosarcoma to undifferentiated sarcoma. Moreover, EWS-FLI1 expression under the control of the *Mx1* promoter resulted in the rapid development of myeloid/erythroid leukemia [29]. The *Prx-1* promoter is active in the primitive mesenchyme of the early limb bud, while the *Mx1* promoter is active in liver, spleen, bone marrow, and lymphoid tissues following induction with type I interferon (IFNα/β). A more recent attempt to create an ES transgenic mouse model utilized Cre-loxP-mediated somatic chromosomal translocation between the *EWS* and *FLI1* locus to express the fusion protein *in vivo* [30]. However, this strategy did not lead to any malignant neoplasms; instead, the mice presented with cardiomyopathy followed by death [30].

Experimental ES models consist of murine xenografts from established human ES cell lines or as allografts of mouse bone marrow-derived mesenchymal progenitors transfected with EWS-FLI1 [17, 19, 21, 31, 32]. The expression of EWS-FLI1 in zebrafish also results in tumor formation, with higher incidences on the p53 null background [33]. However, these models lack the essential elements of tumor initiation, as they are derived from established tumors or cell lines transformed *in vitro*. Therefore, these models do not fulfill the need for a transgenic mouse that develops spontaneous ES driven by EWS-FLI1 expression.

## RESULTS

To develop a clinically relevant ES mouse model for use in studying disease pathogenesis and testing novel therapies, we employed transgenic and non-transgenic approaches to express an *EWS-FLI1* transgene in different tissues at different times. Overall, 16 alternative methods were tried in 6 independent laboratories (Table 1). For simplicity of discussion, these models will be referred to by the numbers provided in Table 1 in this manuscript.

**Table 1:** A summary of sixteen approaches employed by six independent laboratories to express an EWS-FLI1 transgene in mice.

Model #	Target tissue	Promoter	Time of expression	Inducible?	Phenotype	Lab
#1 Runx2Cre-EF	Osteoblast precursor	Cre under Runx2 prom. EWS-FLI1 under Rosa26 prom	Embryonal (E12.5)	No	No phenotype in two clones on WT or INK4a/ARF^-/-^ background. Embryonic lethality (E13.5) in one clone.	Moriggl
# 2 OsxCre-EF	Osteoblast precursor	Cre under Osterix1 prom. EWS-FLI1 under Rosa26 prom.	Embryonal (E14.5) or 3 weeks old	Yes (Tet-off for Cre expression)	Embryonal: Lethal 3 weeks: Facial deformities on WT p53 and Rb 3 weeks: Leukemia and reduced osteosarcoma in p53^-/-^Rb^-/-^ background.	Üren
#3 Col1a2.3Cre-EF and Col1a3.6Cre-EF	Osteoblasts	Cre under Col1a2.3 or Col1a3.6 prom. EWS-FLI1 under EWS prom.	Embryonal (E18.5)	No	Embryonic lethal	Lee
# 4 Cosco-EF	Ubiquitous	EWS-FLI1 under EWS prom.	Embryonal	No	Embryonic lethal	Delattre
#5 Pgk-EF	Ubiquitous	EWS-FLI1 under Pgk prom.	Embryonal	No	Embryonic lethal	Delattre
# 6 Nse-EF and Nse-EF-SV	Neuronal tissue	EWS-FLI1 under Nse prom.	Embryonal	No	Embryonic lethal	Delattre
# 7 NEFL-EF	Neuronal tissue	EWS-FLI1 under dNEFL prom.	Adult	No	EWS-FLI1 expressed in adult brain and cerebellum but no tumor	Delattre
# 8 MT-EF	Ubiquitous	EWS-FLI1 under Metallothionein prom.	Variable	Yes (ZnCl2 for EWS-FLI1 expression)	No phenotype	Delattre
# 9 PLAPtTA-EF	Ubiquitous	tTA under PLAP prom. EWS-FLI1 under hCMV-TRE prom.	Embryonal	Yes (Tet-off for EWS-FLI1 expression)	Embryonic lethal	Delattre
#10 COMET and COMET^ΔNeo^	Ubiquitous	EWS-FLI1 under TRE^tight^ prom.	Variable	Yes (Tet-on for EWS-FLI1-luciferase expression)	EWS-FLI1 toxicity during spermatogenesis in chimera mice, no F1 KI progeny.	Delattre
#11 Prx1Cre-EF	Limb bud Mesenchyme	Cre under Prx1 prom. EWS-FLI1 under EWS prom.	Embryonal (E9.5)	No	Embryonic lethal	Lee
#12 Cre-TL-EF	Mesenchymal and neural crest tissue	Cre under Dermo1, Prx1, P0, Col1a2, or Sox9 prom. EWS-FLI1 under EWS prom.	Embryonal	No	No phenotype on WT or INK4a/ARF^-/-^ background	Sweet-Cordero
#13 RetroLTR-EF	Mesenchymal stem cells	EWS-FLI1 under Retroviral LTR	Adult	No	Fibrosarcoma	Nakamura
#14 piggyBac-EF	Mesenchymal stem cells	EWS-FLI1 under CMV prom.	Adult	No	Fibrosarcoma	Nakamura
#15 CreEP-TL-EF	Not-tissue selective. Cre injected IM	Cre under pMC1 prom. EWS-FLI1 under EWS prom.	4 weeks old	No	Muscle degeneration	Nakamura
#16 Ad5Cre-EF	Not-tissue selective. Virus injected IM, IP and IV	Cre under Ad5-CMV prom. EWS-FLI1 under Rosa26 prom.	1 day old1 week old 3 weeks old	No	IV: No phenotypeIP: Developmental defects in intestinesIM: Muscle atrophy	Üren

### Transgenic approaches: bone and bone precursors

#### EWS-FLI1 expression in osteoblast precursors *via* the *Runx2* promoter (Model #1^Runx2Cre-EF^)

Runx2 is a master transcription factor for chondrocyte and osteoblast differentiation that regulates bone formation [34]. We established a conditional EWS-FLI1 mouse model in which the expression of the fusion protein was controlled by Cre recombinase driven by the *Runx2* promoter in a 150 kB BAC transgene encompassing the *Runx2* gene. Here, an improved *Cre* codon sequence was inserted into the coding exon adjacent to the START codon to drive expression from the bone-specific distal promoter [35] (Supplementary Figure S1). Cre-inducible *ROSA26-loxP-STOP-loxP-EWS-FLI1* (*E/F*) mice [29], in which *EWS-FLI1* is under control of the *ROSA26* gene locus, were used. Therefore, EWS-FLI1 could be ubiquitously expressed following the removal of the STOP codon by Cre recombinase. To restrict and target EWS-FLI1 expression to the bone-forming lineage, *E/F* mice were crossed to *Runx2-Cre* mice. We used three different characterized *Runx2-Cre* transgenic mouse lines (#777, #784 and #1634) that gave different phenotypes. The highest Cre recombinase expression was observed in line #777 compared to lines #784 and #1634 [35].

An analysis of the tissues from *E/F* mice crossed with the #784 and #1634 *Runx2-Cre* transgenic lines (*E/F*+/-*Runx2-Cre*+ mice) showed Cre activity in the bone, bone marrow, calvaria, and testis (Supplementary Figure S2A). Offspring from these mice were viable and fertile (Supplementary Table S1). Careful and regular phenotype screening over 18 months did not reveal any obvious gross abnormalities or tumor formation. The excision of the STOP-cassette in the bone, testis, and isolated osteoblasts was confirmed by genomic PCR (Supplementary Figure S2B). However, *EWS-FLI1* could not be detected at the mRNA level (Supplementary Figure S3A). We failed to detect EWS-FLI1 expression in compound animals of the #784 *E/F*+/- *Runx2-Cre*+ and #1634 *E/F*+/- *Runx2-Cre*+ lines. We also noted the loss of *Cre* expression in compound mesenchymal tissue, although *Cre* was well expressed in mice without the *E/F* transgene (Supplementary Figure S4). In order to confirm that the *EWS-FLI1* locus was amenable to Cre-induced recombination, we isolated ear skin fibroblasts from these lines and expressed Cre recombinase using an adenoviral delivery system in cell culture (Supplementary Figure S5).

Crossing *E/F* mice with the #777 *Runx2-Cre* transgenic line resulted in early embryonic lethality around embryonic day 13.5 (E13.5), and no transgenic offspring could be generated (Supplementary Table S1). We hypothesized that EWS-FLI1 protein expression in the target tissues of the #777 *Runx2-Cre* line caused developmental problems that led to embryo resorption before E13.5 (two litters with 13 embryos were analyzed from *E/F*+/+ female *versus* the #777 *Runx2-Cre* male cross), which thereby excluded the usefulness of that model. We did not further trace the cause of death. Twelve litters of offspring from that cross were analyzed further. While genetic PCR showed that these offspring carried the *E/F* transgene, no #777 *Runx2-Cre* expression in parallel could be detected, suggesting negative selection and the loss of double transgenic embryos during early embryonic development.

EWS-FLI1 has a growth inhibitory effect in most primary cells, and the loss of p16^INK4a^ enables the cells to tolerate EWS-FLI1 expression *in vitro* [14]. Therefore, we postulated that the lethal effect of EWS-FLI1 on *Runx2-Cre*-expressing cells could be relieved by deleting the *p16ink4a* locus. Moreover, the loss of p16^INK4a^ is a relatively common event in ES [36]. Thus, we crossed *E/F*+/- *Runx2-Cre*+ mice (both #784 and #1634 lines) to an *Ink4a*-deficient background. The offspring of these mice were viable (Supplementary Table S1), and a careful analysis of these *E/F*+/- *Runx2-Cre*+ *Ink4a*-/- compound mice showed the same lack of EWS-FLI1-expressing offspring as seen in the *E/F*+/- *Runx2-Cre*+ mice. The deletion of the STOP-cassette was confirmed in the bone and testis, but no expression of EWS-FLI1 could be observed (Supplementary Figure S6). *Ink4a*-/- mice are prone to tumor formation of mainly fibrosarcoma, liposarcoma, angiosarcoma and lymphomas [37]. We observed 30 mice per genotype over 12 months and did not detect any changes in the frequency or histopathological type of tumors in *E/F*+/- *Runx2-Cre*+ *Ink4a*-/- compared to *Ink4a*-/- mice. Often, multiple different tumors occurred in single animals. However, we evaluated more than 10 tumors isolated from the different genotypes and could not detect *Cre* or *EWS-FLI1* expression in any of them. Therefore, we concluded that the toxic effect of EWS-FLI1 expression could not be rescued by the loss of Ink4a protein.

#### EWS-FLI1 expression in osteoblast precursors *via* the *Osterix-1* promoter (Model #2^OsxCre-EF^)

**Figure 1:**
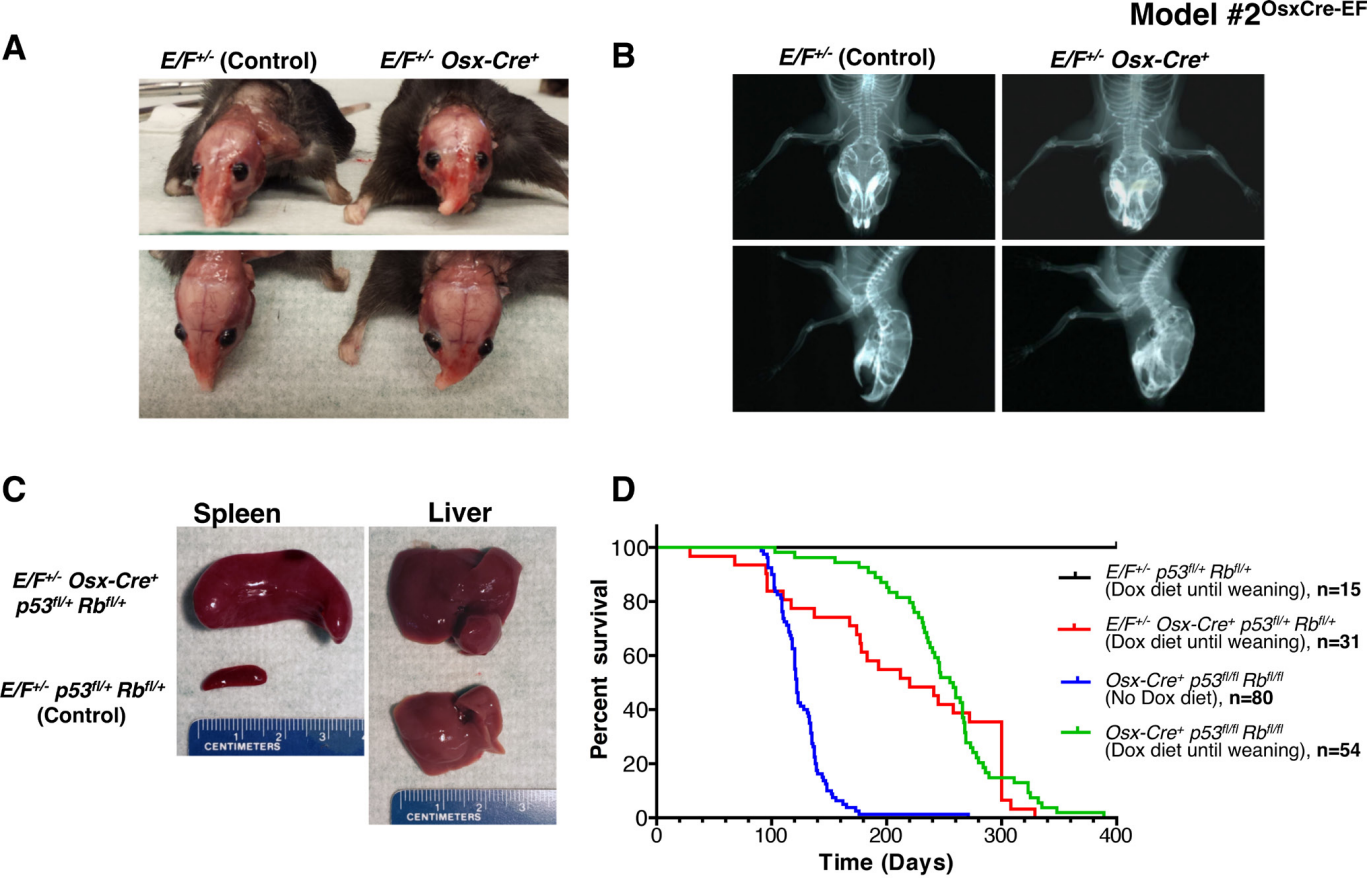
Postnatal expression of EWS-FLI1 in cells with the activated *Osterix* promoter in *E/F*^+/-^ *Osx-Cre*^+^mice with or without *p53* and *pRb* deletion in Model #2^OsxCre-EF^ **A**. Representative presentation of deformed nasal bone of an *E/F*+/- *Osx*-Cre^+^ mouse compared to an *E/F*+/- littermate control. **B**. X-ray imaging of an *E/F*+/- *Osx*-Cre^+^ mouse showing structural abnormalities in facial bones as compared to the clear facial bone structure of an *E/F*+/- littermate control. **C**. Representative photographs of spleens and livers collected from *E/F*+/- *p53*fl/+ *pRb*fl/+ (control) and *E/F*+/- *Osx-Cre*+ *p53*fl/+ *pRb*fl/+ littermates. Hepatomegaly and splenomegaly were consistently observed in *E/F*+/- *Osx-Cre*+ *p53*fl/+ *pRb*fl/+ mice displaying leukemia-like symptoms. **D**. Kaplan-Meier survival plots for the indicated genotypes: *E/F*+/- *p53*fl/+ *pRb*fl/+ (*n* = 15); *E/F*+/- *Osx-Cre*+ *p53*fl/+ *pRb*fl/+ (*n* = 31); and *Osx-Cre*+ *p53*fl/fl *pRb*fl/fl, (*n* = 54) mice with doxycline diet until weaning age. In addition, survival plot for *Osx-Cre*+ *p53*fl/fl *pRb*fl/fl (*n* = 80) mice with no doxycycline diet is presented.

Considering that most ES cases develop in bone or in close proximity to the bone, we hypothesized that the targeted expression of EWS-FLI1 in osteoblast progenitors may induce ES. Osterix (Osx) is a zinc finger-containing transcription factor required for osteoblast differentiation [38], and the promoter restricts Cre expression to the osteoblast lineage [39]. We expressed EWS-FLI1 in osteoblast progenitors by crossing *E/F* mice with inducible Tet-Off-based *Osx-Cre* mice (Supplementary Figure S7). The expression of EWS-FLI1 in *E/F*+/- *Osx-Cre*+ mice with wild-type (WT) *p53* and *Rb* was embryonically lethal. Thus, we delayed *Cre* expression, and hence *EWS-FLI1* expression, until weaning, upon which 32.6% of these mice developed evident facial bone deformities at the age of 5-6 months (Figure 1A and 1B). *Osx-Cre*-mediated deletion of *p53* and *Rb* in both *Osx-Cre*+ *p53*fl/+ *pRb*fl/+ and *Osx-Cre*+ *p53*fl/fl *pRb*fl/fl mice resulted in osteosarcoma, and the latency of the disease could be delayed using a doxycycline diet [40]. When we crossed *E/F* mice with *Osx-Cre*+ *p53*fl/fl *pRb*fl/fl mice, we observed embryonic lethality. The administration of doxycycline to pregnant mothers was required for viable births. When the *EWS-FLI1* expression was delayed until weaning, *E/F*+/- *Osx-Cre*+ *p53*fl/+ *pRb*fl/+ mice did not develop sarcoma; instead, 45.2% of these mice presented with leukemia (Figure 1C and 1D). We confirmed EWS-FLI1 expression in the spleens and livers of these leukemic mice at the mRNA and protein levels (Supplementary Figures S8A and B). Immunophenotyping suggested that the leukemia in *Osx-Cre*+ *p53*fl/+ *pRb*fl/+ mice was similar to the previously reported EWS-FLI-induced erythroid/myeloid leukemia, in which EWS-FLI1 expression was driven by *Mx1-Cre* [29]. As in *E/F*+/- *Mx1-Cre*+ mice, spleen cells were enriched for CD43^+^/CD71^+^/CD117^+^/CD45^-^ cell populations (Supplementary Figure S8C). Although *Osx-Cre* mainly targets osteoblast progenitors, it has also been shown to target non-osteoblast lineage cells in the bone marrow, such as stromal cells, adipocytes and perivascular cells [41]. Therefore, it is possible that EWS-FLI1 is expressed in the hematopoietic lineage and induces leukemia in *E/F*+/- *Osx-Cre*+ *p53*fl/+ *pRb*fl/+ mice as the result of leakiness of the *Osx-Cre* system. Alternatively, extracellular vesicle-mediated systemic *Cre* mRNA exchange may have occurred between osteoblast progenitor cells with the active *Osx* promoter and hematopoietic cells that would not otherwise express Cre [42].

#### EWS-FLI1 expression in osteoblasts *via* the *Col1a1* promoter (Model #3^Col1a2.3Cre-EF & Col1a3.6Cre-EF^)

This mouse model was created by knocking-in (KI) the human *FLI1* cDNA (spanning exons 5-9) with a C-terminal FLAG epitope into mouse *Ews* exon 7 (Supplementary Figure S9 and [16]). This approach closely mimics the translocation found in patients, as EWS-FLI1 is expressed under the control of native *Ews* promoter/enhancer elements. A loxP-flanked STOP-cassette was inserted in the antisense direction in *Ews* intron 6 to permit the conditional expression of EWS-FLI1 [see [16] for details]. The constitutive expression of EWS-FLI1 using an *EIIa-Cre* mouse failed to produce any viable pups [16]. To prevent cytotoxic effects and to direct EWS-FLI1 expression to osteogenic progenitors, we crossed an *Ews-FLI1*KI mouse to two transgenic Cre mice: *Col 3.6-Cre* and *Col 2.3-Cre* [43]. These transgenic lines direct Cre recombinase expression to the osteoblast lineage using 2.3-kb (*Col 2.3-Cre*) and 3.6-kb (*Col 3.6-Cre*) fragments of the rat *Col1a1* promoter. Additional Cre expression in tendons was reported in the *Col 3.6-Cre* mouse only. Upon genotyping the pups at weaning and also at birth (more than 100 combined), no mice carrying both the *Ews-FLI1*KI and *Cre* genes could be obtained, suggesting embryonic lethality resulting from EWS-FLI1 expression in osteoblast progenitors. Alternatively, low but detectable expression of Cre in non-osseous tissues of the *Col 2.3-Cre* and *Col 3.6-Cre* mice (such as the kidney, liver and skin) [43] might have resulted in embryonic lethality.

### Transgenic approaches: ubiquitous or neuronal tissue

#### Ubiquitous expression of EWS-FLI1 *via* the *EWS* or *Pgk* promoter (Model #4^Cosco-EF^ and Model #5^Pgk-EF^)

We tried expressing EWS-FLI1 under broad and highly active promoters in transgenic mouse models to recapitulate the human ES phenotype. A cosmid containing the *EWS* promoter and the full *EWS-FLI1* gene fusion were transfected into murine embryonic stem cells. We generated chimeric mice, but we were not able to obtain stable transgenic founder animals in the F0 generation (Supplementary Table S2, Supplementary Figure S10). We also tried expressing EWS-FLI1 under the control of a murine phosphoglucokinase-1 (*Pgk-1*) promoter (Supplementary Table S2, Supplementary Figure S11). We obtained two female F0 chimeric mice with the transgene, although one female was sterile and the progeny of the fertile mother were negative for the transgene.

#### Expression of EWS-FLI1 in neuronal tissues (Model #6^Nse-EF and Nse-EF-SV^ and Model #7^NEFL-EF^)

As some ES cells express neuronal markers, including neuron-specific enolase (*Nse*), we prepared two transgenic constructs with the murine *Nse* promoter: one contained the *EWS-FLI1* cDNA with the *FLI1* polyadenylation site (*Nse-EF*), and the other contained the *SV40* polyadenylation site (*Nse-EF-SV*). We transfected these two transgenes into murine embryonic stem cells to generate chimeric mice (Supplementary Table S2, Supplementary Figure S12). Among 32 chimeras we created for each model, only one *Nse-EF* chimera was transgenic, but no F1 was obtained from this founder. This result raised the hypothesis of embryonic toxicity in strains where EWS-FLI1 is expressed under constitutive (*EWS, Pgk*) or highly active (*Nse*) promoters. To overcome this problem, we expressed EWS-FLI1 under a minimally active neuron-specific promoter, neurofilament light chain (*NEFL*), as this gene was reported to be expressed in ES cells [44]. In Model #7^NEFL-EF^, we used the human *NEFL* promoter to drive the expression of EWS-FLI1 in mice (Supplementary Table S2, Supplementary Figure S13). Interestingly, four transgenic lines were obtained with Mendelian inheritance of the transgene. RT-PCR for the *EWS-FLI1* transcript confirmed its specific expression in adult brain tissues (Supplementary Figure S13), but no tumor development was observed in these mice even after long-term surveillance (>1 year). This transgenic model may have expressed EWS-FLI1 too late in cellular differentiation to cause the growth of an embryonic tumor because NEFL is only expressed in post-mitotic neurons [45].

#### Inducible expression of EWS-FLI1 *via* ubiquitous promoters (Model #8 ^MT-EF^, and Model #9^PLAPtTA-EF^)

In order to gain better control over the timing of EWS-FLI1 expression, we tried different inducible promoter systems. The metallothionein (*Mt*) promoter, which induces expression of a transgene upon administration of ZnCl_2_ to mice, was used to promote EWS-FLI1 expression in Model #8^MT-EF^ (Supplementary Table S2, Supplementary Figure S14). Four stable transgenic lines were obtained. After ZnCl_2_ administration to the mice (24 h up to several weeks), various tissues were collected, but EWS-FLI1 expression was not detected in any of the tissues. We also used a tet-off system to control EWS-FLI1 expression (Supplementary Table S2, Supplementary Figure S15). In order to conditionally induce *CMV*-driven EWS-FLI1 expression upon doxycycline removal, two stable pUHD-10.3-EF strains were backcrossed with the LT1 strain that expresses the tetracycline-controlled transactivator (tTA) under the control of the human placental alkaline phosphatase (*PLAP*) promoter. Unfortunately, none of these backcrosses (Model #9^PLAPtTA-EF^) gave rise to double transgenic mice.

#### Spatiotemporal regulation of EWS-FLI1 expression (Model #10^COMET and COMETΔNeo^)

**Figure 2:**
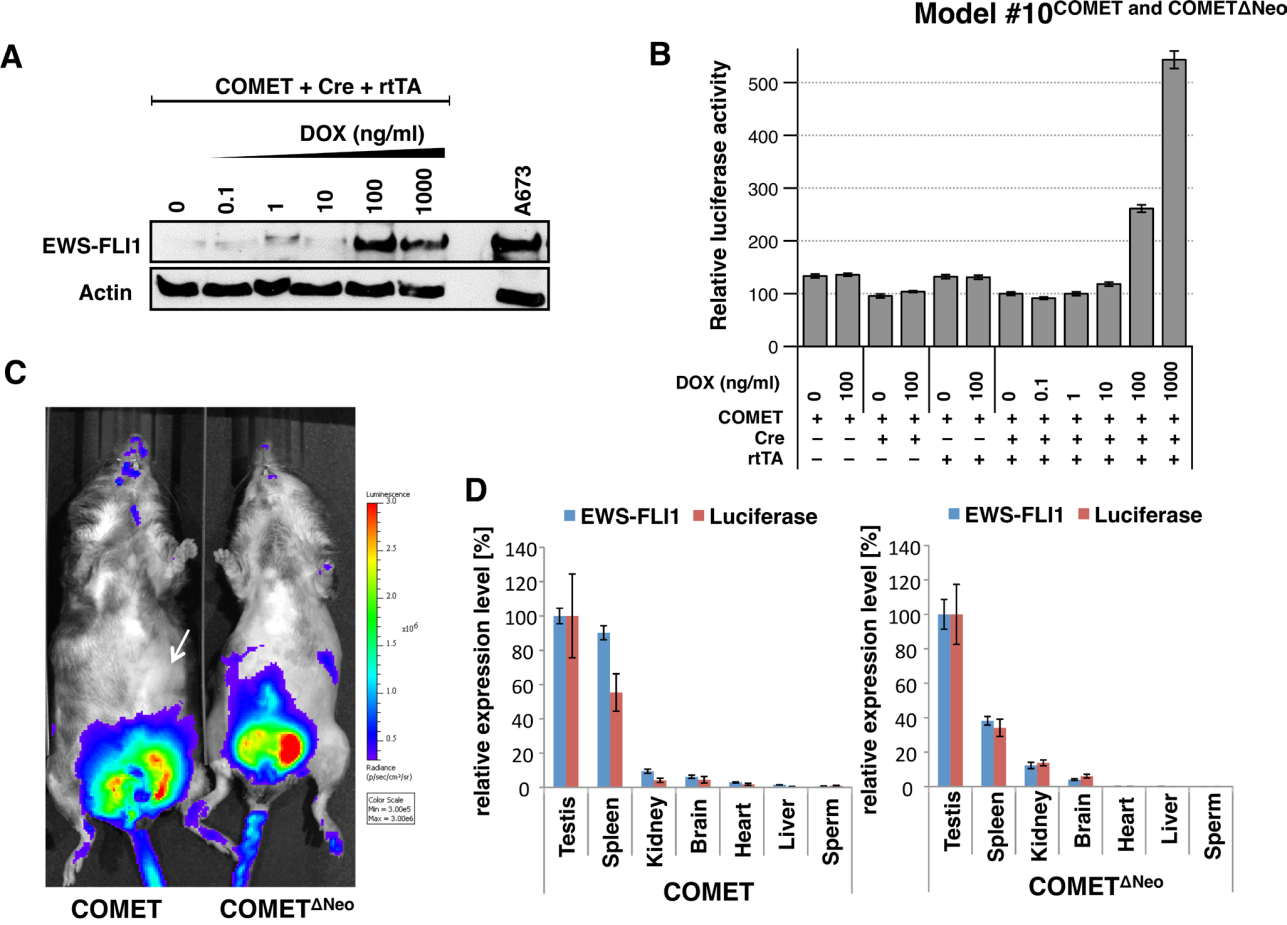
*In vitro* and *in vivo* expression of EWS-FLI1 and luciferase in Model #10^COMET and COMETΔNeo^ **A**. Western blotting against EWS-FLI1 and beta-actin was performed in triple-transfected (COMET + Cre + rtTA) 293T cells treated with increasing amounts of doxycycline (DOX). The A673 ES cell line is shown as the positive control for endogenous EWS-FLI1 expression (right lane). **B**. The COMET construct was transiently co-transfected *in vitro* with (+) or without (-) Cre recombinase and rtTA and in the presence or absence of DOX. Varying amounts of DOX (0 to 1,000 ng/ml) were added to the media. **C**. Models #10^COMET^ (left) #10^COMETΔNeo^ (right) chimera were imaged for *in vivo* luminescence measurements. **D**. Quantitative RT-PCR for *EWS-FLI1* and *luciferase* in #10^COMET^ (left panel) and #10^COMETΔNeo^ (right panel) expression in various organs extracted from the imaged mice in panel C. Relative expression (to testis expression level) of duplicates with respective SD is shown.

Learning from our past unsuccessful attempts (Supplementary Table S2) and from others [15, 16, 28, 29], we aimed to generate an inducible and conditional transgenic *EWS-FLI1* mouse model. We created a knock-in (*Gt(ROSA)26Sor* locus) Cre/lox tetO inducible Mouse model for Ewing Tumor (COMET) (Supplementary Figure S16). For the conditional approach, an antisense *EWS-FLI1* cassette was flanked with single mutated Lox sites (*Lox66, Lox71*). Upon Cre-mediated recombination, *EWS-FLI1* is reversed and maintained in the sense orientation due to the generation of a double mutated *lox72* site (unrecognized by the Cre) and a *loxP* site [46]. For the inducible strategy, a tet-On cassette containing a *TREtight*-inducible promoter oriented in the antisense direction was introduced in the *Gt(ROSA)26Sor* locus, as this was reported to improve its inducibility in this locus [47]. In order to track EWS-FLI1 expression using noninvasive bioluminescence imaging, an *IRES luciferase* (*IRES Luc*) cassette was added downstream of *EWS-FLI1*. A *Frt*-flanked *neomycin selection cassette* was also added. All of these cassettes were finally cloned into the pROSA26-1 vector to generate a pROSA26-1-COMET construct (Supplementary Figure S16). Western blotting against EWS-FLI1 (Figure 2A) confirmed the functionality of the COMET construct *in vitro* by co-transfecting a combination of COMET, Cre and rtTA plasmids in the presence or absence of doxycycline. Chemiluminescence only detected *IRES*-driven luciferase expression in the cells transfected with the COMET, Cre and rtTA constructs in a doxycycline dose-dependent manner (Figure 2B).

To generate Model #10^COMET and COMETΔNeo^, a knock-in strategy into the well-characterized *Gt(ROSA)26Sor* locus was favored over classical transgenesis (Supplementary Figure S16) due to its permanent accessibility for transcriptional activation in mice [48]. Homologous recombination was performed in murine embryonic stem cells with the pROSA26-1-COMET construct, and two positive clones (2C12, 2D8) were confirmed by Southern blotting (Supplementary Figure S17). We injected these two clones into mouse blastocysts. Among seven chimeric mice, only three generated agouti F1. However, none of the 31 F1 agoutis contained the COMET allele (Supplementary Table S3). This led us to speculate about the possible leaky expression of EWS-FLI1 leading to toxicity in Model #10^COMET^ chimeric tissues. To overcome a described bidirectional activity of the PGK promoter that may have led to EWS-FLI1 expression [49], embryonic stem cell clones, 2C12 and 2D8, were transiently transfected with the pCAGGS-Flpe plasmid in order to delete the PGK-Neo selection cassette through FLPe/FRT-mediated excision (Supplementary Figure S16). Two clones deleted for the PGK-Neo cassette (1C3, 2A4) were injected into mouse blastocysts, giving rise to seven Model #10^COMETΔNeo^ chimeric mice. Unfortunately, none of the 33 F1 agoutis contained the Model #10^COMETΔNeo^ allele after backcrossing (Supplementary Table S3).

To determine whether the manipulated genetics of both the Model #10^COMET^ and Model #10^COMETΔNeo^ chimeric mice were functioning as designed, we measured bioluminescence. A clear signal was detected in the testes of both chimeric mouse models (Figure 2C). RNA from various organs of these chimeric mice was extracted, and RT-qPCR experiments for the expression levels of *luciferase* and *EWS-FLI1* were performed (Figure 2D). The highest expression level of luciferase was found in the testis and correlated with *EWS-FLI1* expression. In Model #10^COMET^, comparable mRNA levels were detected in the testis and spleen. However, bioluminescence was absent in the spleen (Figure 2C, white arrow), indicating that the protein may not be correctly processed in this organ.

To investigate the underlying cause of the leaky EWS-FLI1/luciferase expression, various organs were collected from Model #10^COMET and COMETΔNeo^ chimeric mice. As untranslated transcripts of the *Gt(ROSA)26Sor* locus are expressed at high levels in the epididymis and the testis, we evaluated whether the *EWS-FLI1/luciferase* transcript might be expressed from the *Gt(ROSA)26Sor* promoter. In Model #10^COMET^ chimeric mice, using RT-PCR, a fusion transcript between part of exon 1 of the RNA-gene trap *ROSA 26* transcript (NR_027009.1) and the *PGK* promoter was detected only in the testis (Supplementary Figures S18A and B). However, this fusion transcript was expressed at very low levels and may have accounted for only a fraction of the high luciferase expression detected in the testes of Model #10^COMET^ mice. In Model #10^COMETΔNeo^ chimeric mice, a fusion transcript between exon 1 of the RNA-gene trap *ROSA 26* transcript and the *lox71* site (located just upstream of *EWS-FLI1*) could be detected in various organs (Supplementary Figures S18C and D). This fusion transcript was much more abundant than that was detected in Model #10^COMETΔNeo^ mice and may have therefore accounted for a substantial part of the high luciferase expression detected in the testes of Model #10^COMETΔNeo^ mice.

### Transgenic approaches: mesenchymal tissue

#### EWS-FLI1 expression in early limb bud mesenchyme *via* the *Prx1* promoter (Model #11^Prx1Cre-EF^)

In parallel to using Cre driven by the *Col1a1* promoter, we also targeted the expression of EWS-FLI1 to the early limb bud mesenchyme using *Prx1-Cre* transgenic mice (Supplementary Figure S19 and [50]). Again, no viable *EWS-FLI1; Prx1-Cre* positive pups were obtained at birth (more than 100 genotyped). Lin *et al*. reported using the same *Prx1-Cre* mouse to drive EWS-FLI1 expression in 3 transgenic lines [28]. These authors reported severe limb development defects in E14.5 embryos and embryonic lethality in one of their transgenic lines (EF-c), while two other transgenic lines showed milder limb deformities and a normal life span. Because the expression of EWS-FLI1 in our model was driven by the native *EWS* promoter, we speculated that the high levels of EWS-FLI1 expression in the early limb bud mesenchyme may have caused severe developmental defects in the limbs leading to embryonic lethality.

To circumvent the embryonic lethality of EWS-FLI1, in an earlier study, we crossed an *EWS-FLI1*KI mouse with a transgenic mouse expressing Cre fused to a mutated estrogen receptor driven by the *CAG* promoter (*B6.Cg-Tg(CAG-Cre/Esr1)5Amc/J*, Jackson Laboratory). Mice carrying both *EWS-FLI1* and *CreER* genes were viable and healthy. However, the induction of EWS-FLI1 expression with tamoxifen led to the rapid death of the mice, demonstrating the cytotoxic effects of EWS-FLI1 even in somatic cells [16]. Collectively, these results highlight the difficulty in generating an ES mouse model and the importance of limiting EWS-FLI1 expression to a permissive cell type (presumed to be the EWS-FLI1 expression-tolerant tumor cell of origin).

#### Somatic chromosomal translocation between endogenous EWSR1 and FLI1 loci in mesenchymal and neural crest tissue (Model #12^Cre-TL-EF^)

**Figure 3:**
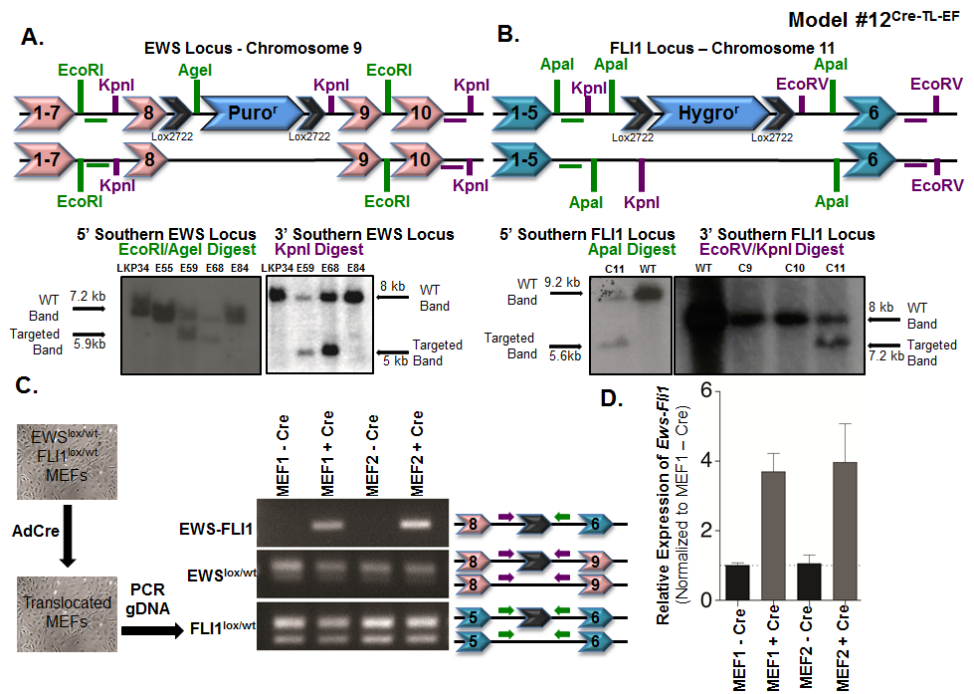
Somatic chromosomal translocation between endogenous *Ewsr1* and *Fli1* loci in Model #12^Cre-TL-EF^ **A**. Targeting mouse embryonic stem cells to insert a *lox-puromycin*r*-lox* cassette between exons 8 and 9 of the *Ewsr1* locus. Genomic DNAs from the embryonic stem cell clones were EcoRI/AgeI (left) or KpnI (right) digested and were analyzed for the 5’ and 3’ integrations using Southern blot. Green and purple horizontal bars represent the probes used in the Southern blots. **B**. Targeting mouse embryonic stem cells to insert a *lox-hygromycin*r*-lox* cassette between exons 5 and 6 of the *Fli1* locus. Genomic DNAs from the embryonic stem cell clones were ApaI (left) or EcoRV/KpnI (right) digested and were analyzed for the 5’ and 3’ integrations using Southern blot. Green and purple bars represent the probes used in the Southern blots. **C**. Schematic illustration for adenoviral Cre infection of *Ews*lox/wt*; Fli1*lox/wt MEFs *in vitro*. Genomic PCR was used to detect the translocated and untranslocated *Ews* and *Fli1* chromosomes. The locations of the primers used are presented in the schematic. **D**. qPCR for *Ews-Fli1* on total RNA from adenoviral Cre-treated MEFs. *Hprt* was used as the control gene, and samples were normalized to uninfected MEFs.

We targeted mouse embryonic stem cells to insert a single *loxP* site into the *Ewsr1* locus and a single *loxP* site into the *Fli1* locus (Supplementary Figure S20). To avoid the potential loss of germline transmission, we chose to generate two separate strains instead of a single embryonic stem cell line in which both loci were targeted. This approach allowed us to delete the selectable markers prior to breeding the *Ewsr1*- and *Fli1*-targeted mice together without concern for extemporaneous recombination events. *Ews*loxpurolox mice were made by inserting a *lox-puromycin*r*-lox* cassette between exons 8 and 9 of *Ewsr1* on chromosome 9. Embryonic stem cell clones surviving puromycin selection were screened by Southern blot analysis for correct targeting (Figure 3A). In addition, Southern blot analysis allowed for a comparison between the intensities of the targeted *Ews*loxpurolox and the non-targeted WT alleles in order to select clones that were likely to have a single targeted and single non-targeted *Ewsr1* allele. We used a similar approach to generate the *Fli1*loxhygrolox mice. A *lox-hygromycin*r*-lox* cassette was inserted into the *Fli1* locus on chromosome 11, between exons 5 and 6 (Supplementary Figure S20). As before, Southern blots were used to confirm correct targeting (Figure 3B). Targeted embryonic stem cells (from non-albino C57BL/6 cells) were injected into albino C57BL/6 blastocysts to generate chimeric mice. Once we obtained chimeras with suspected germline chimerism, we bred them to albino C57BL/6 mice and screened black pups for the presence of the lox-flanked cassettes. Mice carrying the cassettes were then bred to a *CMV-Cre* strain, in which Cre is expressed during implantation of the developing embryo [51]. Early Cre expression resulted in the deletion of the selection cassettes and the generation of mice with a single lox site in the middle of the *Ewsr1* locus (*Ews*lox/wt) and in the middle of the *Fli1* locus (*Fli1*lox/wt), which was confirmed by genomic DNA sequencing (Supplementary Figure S21A). *Ews*lox^/wt^ and *Fli1*lox/wt mice were bred to generate homozygous *Ews*lox/lox and *Fli1*lox/lox mice (Supplementary Figure S20). These homozygous mice were used to generate all of the mice needed for the following experiments.

To test the feasibility of a reciprocal translocation event between chromosomes 9 and 11 *in vivo*, we first verified *in vitro* that this event could occur. Timed breedings between *Ews*lox/lox and *Fli1*lox/lox mice were set up to isolate mouse embryonic fibroblasts (MEFs). These *Ews*lox/wt*; Fli1*lox/wt MEFs were treated *in vitro* with adenovirus expressing Cre recombinase (Ad-Cre) (Figure 3C). PCR analysis using primers from the *Ewsr1* and *Fli1* introns from Ad-Cre-treated MEF genomic DNA confirmed that the recombination event could take place (Figure 3C). The presence of both the WT and lox alleles for *Ewsr1* and *Fli1* demonstrated that the recombination event was not 100%, which was as expected based on published models using this system in mice (Figure 3C). The *Ews-Fli1* and *Fli1-Ews* junctions were sequenced to confirm that the single lox site was flanked by genomic DNA from each chromosome. To determine whether the *Ews-Fli1* gene was transcribed into *Ews-Fli1* mRNA, qRT-PCR was performed using primers that would span *Ewsr1* exon 8 to *FLI1* exon 6. *Ews-Fli1* mRNA was detected in the MEF lines treated with Ad-Cre (Figure 3D). The *Ews-Fli1* cDNA was then cloned using two rounds of nested PCR and sequenced to verify the correct splicing between *Ewsr1* and *Fli1*. The CT values for the qPCR were relatively high, suggesting that *Ews-Fli1* was expressed at a very low level. This low level of *Ews-Fli1* expression may have resulted from the Ad-Cre infection; however, the batch of Ad-Cre had been used routinely in the lab to recombine lox sites found on a single chromosome with very high efficiency. Alternatively, the low level of detected *Ews-Fli1* expression may have resulted from the loss of the cells expressing the translocation due to toxic effects. Attempts to quantify the levels of recombination using fluorescent *in situ* hybridization probes for chromosomes 9 and 11 were unsuccessful. However, given that the qPCR results confirmed that the translocation could be expressed, we hypothesized that even this low level of expression might be sufficient to initiate tumorigenesis *in vivo*.

To establish a physiologically relevant mouse model of ES, the *Ews*lox/lox; *Cre*+ and *Fli1*lox/lox colonies were crossed to generate cohorts of mice to observe for tumors over their life span. The mice in the experimental cohorts were *Ews*lox/+*; Fli1*lox/+*; Cre*+, and those in the control cohorts were *Ews*lox/+*; Fli1*lox/+*; Cre*-. We used 5 different Cre strains to generate tumor study cohorts: *Col1a2-Cre* (Cre^+^ *n* = 16, Cre^-^ *n* = 6), *Dermo1-Cre* (Cre^+^ *n* = 25, Cre^-^ *n* = 18), *P0-Cre* (Cre^+^ *n* = 20, Cre^-^ *n* = 15), *Prx1-Cre* (Cre^+^ *n* = 25, Cre^-^ *n* = 24), and *Sox9-Cre* (Cre^+^ *n* = 21, Cre^-^ *n* = 15). *Dermo1*-*Cre* mice crossed with a LacZ reporter showed expression in mesenchymal tissues as early as E9.5; later, expression was detected in the condensing mesenchyme, chondrocytes and osteoblasts [52]. *Col1a2*-*Cre* showed a broader staining pattern than *Dermo1-Cre*, as LacZ expression was found starting around E8.5 in all dermal cells of the mouse as well as in the chondrocytes and osteoblasts of the developing bones [53]. The expression of *Prx1-Cre* began around E10 in the undifferentiated mesenchyme of the developing limb buds, increasing in expression with the development of the limb buds, in the mesenchyme between the limbs, and in the non-neural mesenchyme in the brain [50]. Some *Prx1*-expressing cells later expressed *Sox9-Cre*, giving us a subpopulation of *Prx1-Cre* cells to study [54]. The cells derived from the *Sox9* lineage in the limb bud include chondrocytes and osteocytes, the cells that form tendons as well as the synovium (the soft tissue that lines the joints). A caveat of *Sox9-Cre* is that it has been shown to be expressed more broadly in various organ progenitor populations. To test whether a neural crest stem cell is the cell of origin for ES, we used the *P0-Cre* strain to drive Cre expression during the development of the neural crest lineage [55]. *P0-Cre* expression was shown to begin as early as E9.0 in tissues derived from the neural crest, and later in development, *P0-Cre* expression spread to the tissues derived from migrating neural crest cells. The notochord, which is not derived from the neural crest, also showed positive expression.

In addition to the above tumor study cohorts, a smaller cohort using *CMV-Cre* mice was observed to test whether the Cre expression that occurred very early in the developing embryo would show an embryonic lethal phenotype or a tumor phenotype [51]. Whereas *Dermo1-Cre* and *Col1a2-Cre* bred to *E/F* mice resulted in embryonic lethality, we obtained mice born at Mendelian ratios for all Cre strains without any lethality [29]. All of the *CMV-Cre*+ (*n* = 12) and *CMV-Cre*- (*n* = 9) mice were observed for >80 weeks without any differences. For the tumor cohorts with the mesenchymal and neural crest Cre strains, no signs of tumorigenesis were apparent after 70 weeks, so the cohorts were euthanized for analysis. Unfortunately, there were no differences between any of the *Cre*+ cohorts compared to the *Cre*- cohorts, and no obvious macroscopic tumors reminiscent of sarcomas were found (Supplementary Figures S22A-C). These results suggested either (i) the single hit of EWS-FLI1 expression in a subset of cells was not sufficient to induce tumor formation in the mouse (e.g. EWS-FLI1 might not have been expressed in enough cells to facilitate tumorigenesis) or (ii) the *Ews-Fli1* translocation event never occurred in the first place.

We have shown that the *Ews-Fli1* reciprocal translocation event did not induce tumor formation when expressed in mouse tissues using specific Cre strains. To test whether the loss of the tumor suppressors *Ink4a* and *Arf* would enable tumor formation or whether EWS-FLI1 was able to alter the tumors formed with *Ink4a/Arf* loss, cohorts of mice were generated such that Cre expression would delete the *Ink4a/Arf* locus as well as induce *Ews-Fli1* translocation. The main groups of this study consisted of mice with the genotype *Ews* lox/+*; Fli1*lox/+*; Ink4a/Arf*flox/flox*; Cre*+, which were tested using the following Cre strains: *Col1a2-Cre*, *Dermo1-Cre*, *P0-Cre*, *Prx1-Cre*, or *Sox9-Cre*. The control mice lacked Cre (*Ews*lox/+*; Fli1*lox/+*; Ink4a/Arf*flox/flox*; Cre*-) or were hemizygous for the floxed *Ink4a/Arf* allele (*Ews*lox/+*; Fli1*lox/+*; Ink4a/Arf*flox/+*; Cre-* and *Ews*lox/+*; Fli1*lox/+*; Ink4a/Arf*flox/+*; Cre*+). In addition, cohorts of mice with the genotypes *Ews*lox/lox*; Ink4a/Arf*flox/flox*; Cre*+ and *Ews*lox/lox*; Ink4a/Arf*flox/flox*; Cre*^-^ using *Dermo1*-*Cre*, *P0-Cre* and *Prx1-Cre* were generated to test the effects of *Ink4a/Arf* loss itself on tumorigenesis. Mice were observed until they appeared moribund or they reached the age of 70 weeks. The survival curves for these mice demonstrated that EWS-FLI1 did not enhance or change the tumor phenotype of these mice (Supplementary Figures S22D-F). A summary of the types of tumors and the number of mice with each type of tumor are presented (Supplementary Table S4). The loss of *Ink4a/Arf* alone either with or without EWS-FLI1 expression led to decreased survival of the mice such that the majority of the *Ink4a/Arf*flox/flox*; Cre*+ mice died earlier than the control mice. To confirm that EWS-FLI1 was not expressed in the tumors that developed in these mice, total RNA from tumor tissues was isolated and qRT-PCR for *Ews-Fli1* was performed. By normalizing to a line that did not express mouse *Ews-Fli1* and using a positive control for mouse *Ews-Fli1* expression (mouse *EWS-FLI1*-overexpressing MEFs), we demonstrated that none of the tumors from the *Ink4a/Arf* tumor study expressed *Ews-Fli1* at levels above background (Supplementary Figure S21B).

#### Expression of EWS-FLI1 in murine mesenchymal stem cells (MSCs) (Model #13^RetroLTR-EF^ and Model #14^piggyBac-EF^)

**Figure 4:**
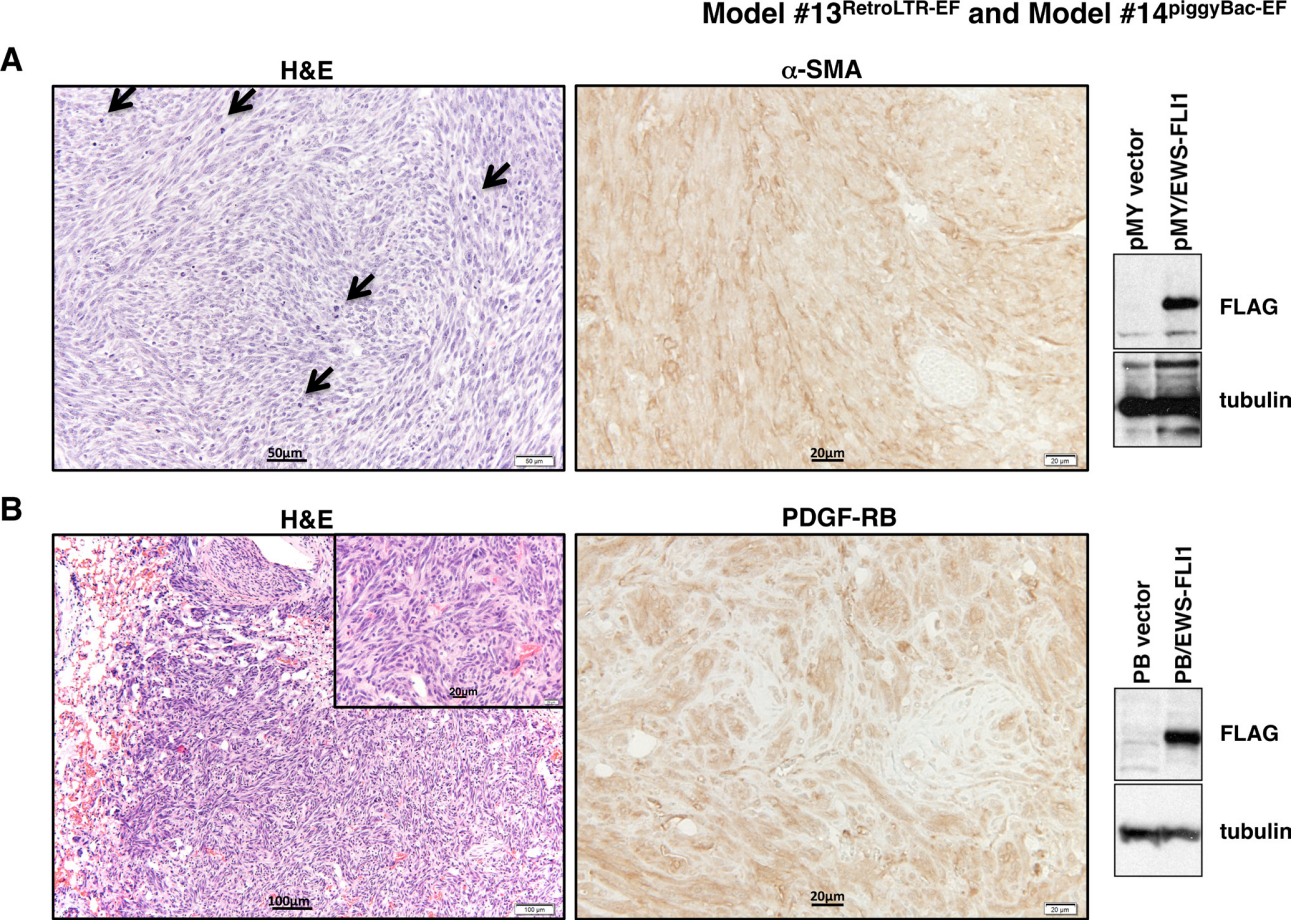
Histopathological analysis of tumors from Model #13^RetroLTR-EF^ and Model #14^piggyBac-EF^ **A**. Fibrosarcoma developed in Model #13^RetroLTR-EF^. The storiform pattern of spindle-shaped, pleomorphic tumor cells is remarkable. Frequent mitotic figures (arrows) indicate aggressive tumor growth (left). α-SMA is a marker of smooth muscle and myofibroblastic cells. Human fibrosarcoma stains positive for α-SMA, whereas ES stains negative (middle). Expression of EWS-FLI1 in tumor tissue was confirmed using an anti-FLAG M2 antibody (right). **B**. Fibrosarcoma with a similar histology as (A) was also induced in Model #14^piggyBac-EF^. Invasive growth of the tumor in lung tissue is noted (left). PDGF-RB is a mesenchymal marker that is frequently positive in human fibrosarcoma and negative in ES (middle). The expression of EWS-FLI1 was confirmed by western blotting (right).

MSCs are characterized by self-renewal activities and multi-directional differentiation [56]. It was expected that the cellular plasticity of MSCs might help EWS-FLI1 to function as a proper ES oncogene. We expressed EWS-FLI1 in bone marrow-derived MSCs (BM-MSCs; [57]) using retrovirus- or transposon-mediated gene transfer. FLAG-tagged *EWS-FLI1* was inserted into the pMYs-IRES-Neo retroviral vector (Supplementary Figure S23). BM-MSCs of a BALB/c background were transduced with the retrovirus and then injected into sublethally irradiated (8 Gy) BALB/c recipients *via* the tail vein. All recipients (*n* = 5) developed multiple tumors in the lungs three weeks after injection. Histologically, the tumors exhibited storiform growth of spindle cells with frequent mitotic figures, which was consistent with the characteristics of fibrosarcoma (Figure 4A). The *EWS-FLI1* transposon construct was generated using the *piggyBac* (PB) transposon/transposase system [58] (Supplementary Figure S24). BM-MSCs were transfected with PB-EF along with mRNA of the *piggyBac* transposase to increase the transfection efficiencies. The transfectants were transplanted into irradiated recipients as above (*n* = 5) or transplanted subcutaneously (*n* = 9). Again, 100% of the recipients developed fibrosarcoma-like tumors within 8 weeks (Figure 4B). Thus, although EWS-FLI1 expression could induce neoplastic transformation of BM-MSCs, the lesions did not recapitulate the morphologic characteristics of the small, round cells observed in human ES.

### Non-transgenic approaches: localized Cre delivery

#### Localized Cre delivery *via in vivo* electroporation in EWS-FLI1 translocation mice (Model #15^CreEP-TL-EF^)

Localized expression of EWS-FLI1 was also tested in the *Cre-loxP*-mediated somatic chromosomal translocation model mice [30]. A Cre expression plasmid, *pMC1-Cre*, was delivered into the muscles of the lower legs of *Ewsr1*fl/+*:Fli1*fl/+ mice *via* electroporation (Supplementary Figure S25). The delivery of Cre *via* electroporation *in vivo* was successfully achieved to induce pleomorphic rhabdomyosarcoma in adult mice [59]. The mice received *in vivo* electroporation of the Cre plasmid four times and were observed for 1 year. No CreEP-TL-EF mice (*n* = 12) developed neoplastic lesions in the muscle, though the mice did show localized degeneration of the muscle fibers (Supplementary Figure S26), which was comparable to the damage in the myocardium in Cag-Cre:*Ewsr1*fl/+*:Fli1*fl/+ mice [30].

#### Localized adenovirus-mediated Cre delivery in *E/F* mice (Model #16^Ad5Cre-EF^)

Expressing EWS-FLI1 in most tissues resulted in embryonic lethality, as described in earlier models. To circumvent the reliance on tissue-specific promoters and to overcome embryonic lethality, we utilized adenovirus-delivered Cre to allow for the localized expression of EWS-FLI1 at different stages of postnatal development in *E/F* mice (Supplementary Figure S27). Adenovirus-Cre-mediated recombination has been successfully used to generate sporadic lung cancer [60], pancreatic adenocarcinoma [61], and colon cancer [62] mouse models. We hypothesized that the target cell may be present or susceptible to EWS-FLI1 tumorigenicity at certain stages of postnatal development. Therefore, adenovirus-Cre (Ad5-Cre) was delivered *via* intramuscular (IM), intraperitoneal (IP), or intravenous (IV) injections at different ages.

**Figure 5:**
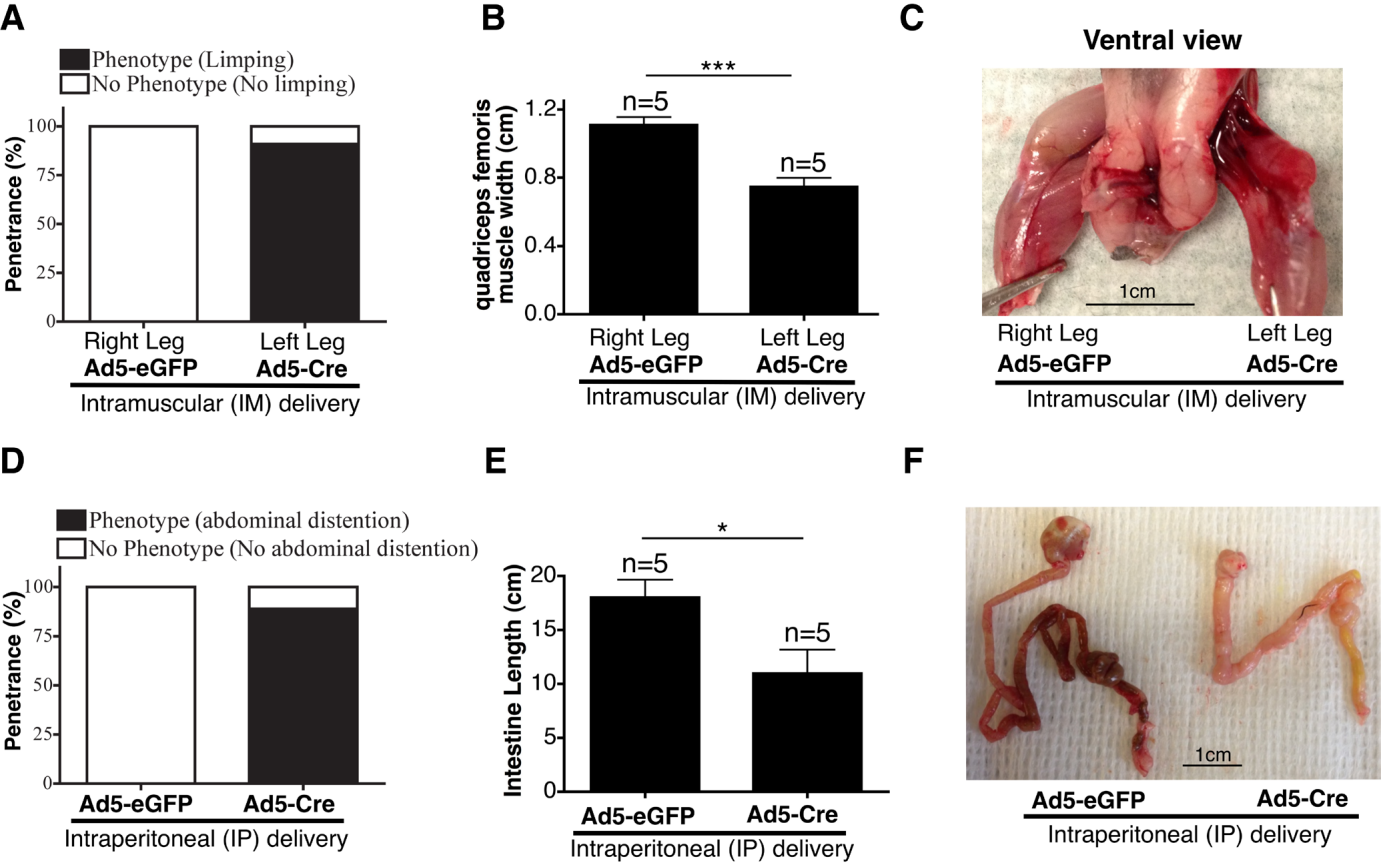
Intramuscular and intraperitoneal delivery of adenovirus-Cre in 1-day-old *E/F*^+/+^ mice in Model #16^Ad5Cre-EF^ **A**. Penetrance of limping phenotype observed in Ad5-Cre-injected left leg *vs*. Ad5-eGFP-injected right leg (*n* = 11) at an age of 1 day. **B**. Comparison of quadriceps femoris muscle width between an Ad5-eGFP-injected right leg and Ad5-Cre-injected left leg. **C**. Representative image showing muscle atrophy observed in Ad5-Cre-injected leg. **D**. Penetrance of abdominal distention phenotype observed in 1-day-old mice IP-injected with Ad5-Cre (*n* = 9) *vs*. Ad5-eGFP (*n* = 7). **E**. Comparison of intestine length (from stomach to rectum) in 1-day-old mice IP injected with Ad5-Cre *vs*. Ad5-eGFP. **F**. Representative image showing shortened intestines observed in Ad5-Cre-injected mice compared to littermate mice of the same genotype injected with Ad5-eGFP.

For IM delivery, *E/F* mice were injected with 10^9^ plaque-forming units (pfu) Ad5-Cre in the left leg and 10^9^ pfu Ad5-eGFP in the right leg at 1 day, 1 week, or 2 weeks of age. Cre mediated removal of the STOP-cassette 48 h after IM delivery of Ad5-Cre was confirmed using genomic DNA PCR (Supplementary Figure S28A). We observed muscle loss, limping, and prolonged clasping of the hind leg in the Ad5-Cre injected leg, but not in the Ad5-eGFP injected leg (Figure 5A and Supplementary Figure S28B). Significant muscle loss was observed in the Ad5-Cre-injected left leg compared to the Ad5-eGFP-injected right leg of the same mouse, which was quantitated as the width of the quadriceps femoris muscle (Figure 5B and 5C). EWS-FLI1 expression has been previously reported to induce caspase 3 transcription, which subsequently triggers apoptosis *in vivo* [16]. EWS-FLI1 expression in cultured cardiac muscle cells also induced apoptosis *in vitro* and cardiomyopathy *in vivo* [30]. Hence, the observed myopathy in the legs injected with Ad5-Cre may have been due to the apoptotic effect of EWS-FLI1 in the myocytes in which Cre-mediated recombination may have occurred. This phenotype was more evident in mice injected at day 1 postnatal compared to the mice injected at 1 or 2 weeks, in which the penetrance of the phenotype observed was 90.9%, 9.1%, and 11.1%, respectively (Figure 5A and Supplementary Figure S28C). This observation may have indicated a differential sensitivity of murine muscle cells to EWS-FLI1 expression during early postnatal development. Alternatively, the variability in the penetrance of the phenotype may have been because the dosage per body weight was much higher in the 1-day-old mice compared to the older mice injected with the same number of Ad5-Cre viral particles. None of the IM-injected mice developed sarcoma when they were observed up to 9 months of age.

For IP delivery, *E/F* mice were injected with 10^9^ pfu of Ad5-Cre at 1 day or 1 week of age. As the negative control, we used 10^9^ pfu Ad5-eGFP injected littermates of the same age and genotype. Cre expression was confirmed 48 h after IP delivery of Ad5-Cre-eGFP using Maestro fluorescence imaging (Supplementary Figure S29A). Three weeks following injection in 1-day-old mice, most of the Ad5-Cre mice presented with abdominal distention (Figure 5D and Supplementary Figure S29B). The Ad5-Cre-injected *E/F* mice also showed significantly shortened small intestines (Figures 5E and 5F). Additionally, we observed malformed livers in the Ad5-Cre-injected mice; the liver had a more globular shape compared to the normal disc shape (Supplementary Figure S29C). The same phenotypes were present in the mice injected with Ad5-Cre at 1 week of age (Supplementary Figure S29D). The *E/F* mice intraperitoneally injected with Ad5-Cre at 1 week of age survived up to 6 months, but none of them developed any detectable ES. Histopathological analysis of the intestines did not reveal any defects in the villi or crypt structures in the majority of the intestine, but there were small areas of necrosis and perforation in the wall in multiple locations in each mouse, which may have led to leakage into the peritoneal cavity that caused distention of the abdomen (Supplementary Figure S30). The growth arrest and apoptotic effects of EWS-FLI1 expression may explain the defects in small intestine development [14–16, 63]. The ability of EWS-FLI1 to antagonize β-catenin/TCF-mediated transcription may have also contributed to the shortened small intestine phenotype [64, 65]. Furthermore, as an effort to deliver Cre recombinase systematically, we injected 10^9^ pfu Ad5-Cre intravenously in 3-week-old *E/F* mice; their littermates were injected with 10^9^ pfu Ad5-eGFP as a control. Over 7.5 months of follow-up, neither the Ad5-Cre- nor the Ad5-eGFP-injected mice presented with any evident phenotype.

## DISCUSSION

Several pediatric sarcomas contain tumor-specific chromosomal translocations that produce chimeric proteins with novel functions. These neoplasms include alveolar rhabdomyosarcoma, myxoid liposarcoma, synovial sarcoma, and clear cell sarcoma harboring *PAX3:FKHR* t(2;13), *TLS-CHOP* t(12;16), *SYT-SSX* t(X;18), and *EWS-ATF1* t(12;22) chromosomal translocations, respectively [22, 23, 66, 67]. Similar to the role of EWS-FLI1 in ES, both Pax3:Fkhr and SYT-SSX have been shown to play critical roles in the malignant phenotypes of rhabdomyosarcoma and synovial sarcoma cell lines, respectively. These observations led to development of useful transgenic mouse models for both tumors. When PAX3:FKHR expression was targeted by the *Myf6* promoter in terminally differentiating skeletal muscle, mice developed alveaolar rhabdomyosarcoma with a very low penetrance (< 1%). However, when the transgene was expressed on the *Ink4a/Arf* or *p53* null background, the penetrance increased to 30-40% [24]. Similarly, the expression of the TLS-CHOP chromosomal translocation product under the control of the *Prx1* promoter (in early mesodermal tissue) induced myxoid liposarcomas only on the *p53* null background [25]. Moreover, the expression of the EWS-ATF1 chromosomal translocation product in neural crest-derived cells resulted in clear cell sarcomas [26], and when SYT-SSX expression was induced in Myf5-expressing myoblasts, 100% of mice developed synovial sarcoma-like tumors [27]. Interestingly, the induction of SYT-SSX expression through *Hprt-Cre*, *Pax3-Cre*, or *Pax7-Cre* resulted in embryonic lethality, and SYT-SSX expression in Myf6-expressing myocytes or Myf6-expressing myofibers resulted in myopathy but no tumors. Therefore, tumor-specific chromosomal translocation products can produce specific types of sarcomas in mice when they are expressed at the right time, in the right cell population and, in some cases, with the help of deleting tumor suppressors.

The successes in developing clinically relevant mouse models for rhabdomyosarcoma, myxoid liposarcoma, clear cell sarcoma, and synovial sarcoma have not been translated to ES, regardless of an exhaustive series of modeling attempts. Several factors have contributed to the failure of developing an ES transgenic mouse model by expressing EWS-FLI1. In most cell types, the expression of EWS-FLI1 induces growth arrest or apoptosis [14–16, 63]. Our findings are consistent with the detrimental effect of EWS-FLI1 expression in sensitive tissues, as we repeatedly observed embryonic lethality or developmental defects. Cooperation from the loss of additional tumor suppressor genes was not observed in our experiments, demonstrating that the correct dosage and timing of EWS-FLI1 expression was not met in these models despite multiple efforts and protocols used. The loss of *p53* and *Rb* or *p16* and *p19* did not result in ES formation in the presence of EWS-FLI1.

The exact cell of origin for ES remains uncertain, which further impairs our ability to target the correct ontologic cell at the correct developmental stage. Even though many publications support the hypothesis that MSCs may be the cell type of origin [17, 18, 68–71], the lack of lineage-specific promoters as well as a range of MSC phenotypes prevents targeting EWS-FLI1 expression in these cells. In our studies, we used several promoters (Model #11^Prx1Cre-EF^, Model #12^Cre-TL-EF^, Models #13^RetroLTR-EF^ and #14^piggyBac-EF^) that can target EWS-FLI1 expression in different mesenchymal tissues without any success in generating ES in mice.

For Model #1, we concluded that the cells expressing high levels of Cre, which resulted in high EWS-FLI1 expression, did not survive. Following Cre-induced recombination, cells either died by apoptosis or were cleared by the immune system. Bone development is a dynamic process, and normal cells with lost Cre and EWS-FLI1 expression overtook the proper development of bone structures. One possible explanation for our negative results with the *E/F*+/- *Runx2-Cre*+ lines could be that the binding of EWS-FLI1 onto the *Runx2* promoter negatively regulates Runx2 activity, which could inhibit differentiation of mesenchymal cells [72]. One could assume that EWS-FLI1 down-regulates Runx2 at the transcriptional level, and this is consistent with the lack of Cre expression, which itself retains a certain degree of toxicity.

EWS-FLI1 expression driven by *Col 2.3-Cre* or *Col 3.6-Cre* proved fatal to developing embryos. The original study reported robust Cre activity in osteoblast progenitors in E18.5 to postnatal day 5 animals [43]. However, it is unknown whether Cre activity is detected earlier in development. Furthermore, low but detectable levels of Cre activity were reported in the non-osseous tissues of these mice, suggesting the possibility that non-osseous EWS-FLI1 expression caused embryonic lethality. Targeted expression of EWS-FLI1 in the early limb bud mesenchyme (E9.5) with *Prx1-Cre* was also fatal. Based on the abundant EWS expression levels in most tissues, it can be surmised that the native *EWS* promoter drives the high level of EWS-FLI1 expression upon *Prx1-Cre* activation, leading to embryonic lethality. Embryonic lethality was also observed in the *Prx1-Cre*-driven EWS-FLI1 transgenic line [28].

Accumulating evidence suggests that EWS-FLI1 may support oncogenic phenotypes beyond its role as a DNA-binding transcription factor. Recent epigenomic studies have identified EWS-FLI1 as an epigenetic driver. Similarly to MYC, EWS-FLI1 may function as a transcriptional amplifier of gene expression by binding to open promoters of widely expressed genes; EWS-FLI1 may also activate novel enhancers and superenhancers [73, 74]. It is therefore possible that the tissue- or stage-specific chromatin accessibility to key genes of ES tumorigenesis differs between mice and men, thus vexing our attempts at modeling [75].

Alternative splicing is emerging as an important mechanism in carcinogenesis [76]. Alternative splicing of the same gene may result in two proteins with opposite functional roles. *BCL2L1* and *FAS Receptor (TNR6)* are two examples that can create both pro-apoptotic and anti-apoptotic protein products as a result of excluding or including a specific exon. EWS-FLI1 interacts with key proteins in the splicing complex and regulates alternative splicing of a specific set of genes that do not always overlap with transcriptional target genes (FLI1 target genes) [77–81]. Therefore, EWS-FLI1 may function through regulating transcription of certain genes and regulating translation of a different set of genes. The homology between human and mouse genes does not always cover the alternative splicing sites. Due to differences in alternative splicing sites between mice and humans, the expression of EWS-FLI1 in human cells may potentially result in a different set of alternatively spliced products than the products in murine cells. If these sets of genes are critical for ES development, then creating an ES model in mice by expressing EWS-FLI1 may not be possible.

Another difference between the human and mouse genomes involves microsatellite sequences. EWS-FLI1 regulates target gene expression through GGAA microsatellite response elements [82–84]. The number of GGAA microsatellite motifs and their distance to promoters significantly alter target gene expression [85–87]. Furthermore, recent findings indicate that ES patients preferentially carry the A risk allele (compared to T) of the rs79965208 single-nucleotide polymorphism (SNP). An ES genome-wide association study identified microsatellite susceptibility variants near the *EGR2* gene. The increase in the length of GGAA microsatellites may therefore contribute to EWS-FLI1 oncogenesis [88], but this microsatellite and SNP are not conserved in mice. Therefore, even though the human and mouse genomes may have similar EWS-FLI1 target genes, their expression patterns may be completely different due to differences in GGAA microsatellite motifs between the human and mouse genomes.

Human ES cells express high levels of cell surface protein CD99, which is routinely used for confirming diagnosis [89–92]. In addition to its diagnostic value, inhibition of CD99 expression or engagement by CD99 antibodies stop growth of ES cell lines both in culture and in xenograft models [93–97]. Mouse homolog of CD99 has less than 50% amino acid identity to its human counterpart [98, 99]. Therefore, biological pathways regulated by human CD99 and mouse CD99 may show significant differences. Because human CD99 is a critical component of ES pathogenesis, it is possible that the lack of a comparable protein in mouse cells may be responsible for the lack of ES development from EWS-FLI1 expression.

ES patients harbor a balanced chromosomal translocation, which generates two chimeric fusion genes, *EWS-FLI1* and *FLI1-EWS*. Many researchers in the field could not detect expression of FLI1-EWS mRNA or protein in ES cell lines or human tumor samples. However, a recent publication provided data suggesting that FLI1-EWS is expressed in ES cells and more importantly FLI1-EWS expression is required for EWS-FLI1 mediated transformation [100]. If this hypothesis is true, the lack of FLI1-EWS component in our attempts might have been responsible for the failed mouse models.

In summary, the ectopic expression of EWS-FLI1 in murine MSCs, neural crest-derived stem cells, or osteochondrogenic progenitor cells could transform primary cells into malignant tumor cells. However, achieving tumor growth in animals with intact immune systems is a higher-level challenge than anchorage-independent growth or tumor development in immunocompromised mice with cells transformed *in vitro*. Hence, it remains possible that an EWS-FLI1-driven sarcoma model can be generated, although the number of failed attempts at creating a transgenic model suggest that this challenge has yet to be accomplished. This comprehensive analysis of those models that have been attempted, without success, should allow future investigations to advance without repeating unsuccessful models.

## MATERIALS AND METHODS

All procedures involving mice were approved by the respective institutions’ animal care and use committees.

### Model #1^Runx2Cre-EF^

Mice were kept under standardized conditions at the Decentralized Biomedical Facility of the Medical University of Vienna. *E/F* mice harboring a Cre-inducible EWS-FLI1 knocked into the ubiquitous *ROSA26* locus (*ROSA26loxP-STOPloxP-HA-EWS-FLI1* allele) [29] were crossed to three different *Runx2-Cre* isoforms [35]. *Runx2* was expressed from promoters *p1* and *p2* [101]. These strains were generated with a BAC (bacterial artificial chromosome) approach with different transgenic integrations and copy numbers (#777, #784, #1634). *E/F*+/-*Runx2-Cre*+ mice were also bred to mice deficient in p16^INK4A^ and p19^ARF^ proteins [37]. All mice were on mixed background (Sv129 and C57BL/6).

### Model #2^OsxCre-EF^

#### Mice

*E/F* mice were generously provided by Dr. S. Baker (St. Jude Children's Research Hospital) and were on a C57BL/6 background. *Osx-Cre* mice on a C57BL/6J background were obtained from the Jackson Laboratories (Bar Harbor, ME). *Osx-*Cre+*p53*fl/fl *pRb*fl/fl animals were generously provided by Dr. Stuart Orkin (Harvard University) and were on a C57BL/6J 129 FVB/n hybrid background. *E/F* mice were maintained as homozygotes (*E/F*+/+) and bred with either *Osx-*Cre^+^ or *Osx-*Cre^+^*p53*fl/fl *pRb*fl/fl animals. Mice received doxycycline diet (2000mg/kg diet, Harlan Laboratories) throughout the pregnancy (average 21 days) and withdrawn at the weaning age of pups (postnatal day 21). Mice were euthanized if the animals showed signs of pain and distress or when the animals reached the age of 300 days.

#### Flow cytometry

For cell surface analysis of splenocytes, harvested spleens were minced into small pieces in 1x PBS to release the blood cells, which were subsequently strained using 70 μm cell strainer (Fisher, Cat No. 352350) to prepare a single cell suspension. The strained mix was then subjected to centrifugation at 350 g for 5 min. Pelleted cells were resuspended in 1x PBS and spleen cell count was determined using hemocytometer. 0.25 μl of CD43-PE (Clone S11, BioLegend, Cat No. 143205), 0.5 μl of CD45-Alexa Fluor-488 (Clone 30-F111, BioLegend, Cat No. 103122), 1 μl of CD71-Brilliant Violet 421 (Clone RI7217, BioLegend, Cat No. 113813), and 2.5 μl of CD117-APC (Clone 2B8, BioLegend, Cat No. 105812) fluorescent conjugated primary antibodies were used to stain one million splenocytes in 100 μl 1x PBS for 20 min on ice in the dark. The stained cells were washed twice with 1x PBS. The cells were then resuspended in 500 μl of 1x PBS and analyzed using flow cytometer.

#### Western blot

Total protein extracts were prepared from spleens and livers of *E/F*+/- *Osx-Cre*+ *p53*fl/+ *pRb*fl/+ mice using the following protocol. Small fragments (˜2mm^3^) of flash frozen spleens were homogenized using hand held mortar mixer (VWR, Cat. No: 47747-370) in 200 μl phospholysis buffer (50 mM HEPES pH 7.9, 100 mM sodium chloride, 4.0 mM sodium pyrophosphate, 10 mM EDTA, 10 mM sodium fluoride, and 1% Triton X-100 v:v) containing 2.0 mM sodium vanadate, 1.0 mM PMSF, 4.0 µg/ml aprotinin, and 4.0 µg/ml leupeptin. Once the spleens were fully homogenized, the lysates were incubated on ice for 30 min. Next, the lysates were subjected to centrifugation for 10 min at 16,000 g at 4°C. Proteins were denatured in 5x Laemmli sample buffer and subjected to SDS-PAGE (10% polyacrylamide). Resolved proteins were transferred to 0.45 μm Immobilon-P PVDF membrane (Millipore, Cat No. IPVH00010). The membranes were blocked in 5% nonfat dry milk in 1x TTBS (20 mM Tris-HCl, pH 7.5, 150 mM NaCl, 0.5% Tween 20 v:v) for 2 hrs. Dilutions for primary antibodies were anti-FLI1 (Santa Cruz Biotechnology, Cat No. sc-356) at 1:1000, anti-HA (Roche, Cat No. 1867423) at 1:500, and anti-actin-horseradish peroxidase (C-11, Santa Cruz Biotechnology, Cat No. sc-1615 ) at 1:5000. Primary antibodies were added to the membranes in 5% nonfat dry milk in 1x TTBS for 2hrs at room temperature. The membranes were then washed three times in 1x TTBS and incubated for 1h with 1:5000 dilution of horseradish peroxidase-linked anti-rabbit (GE Healthcare, Cat No. LNA934V/AG) or anti-rat secondary antibody (R&D, Cat No. HAF005) prepared in 5% nonfat dry milk. Blots were washed three times in 1x TTBS and then developed using Immobilon Western Chemiluminescent HRP Substrate per the manufacturer's instructions (Millipore Corporation, Cat No. WBKLS0100). Chemiluminescence was detected using a Fujifilm LAS-3000 imaging system.

#### RT-qPCR

RNA was extracted from ˜50 mg flash frozen spleen fragments using TRIzol according to the manufacturer's protocol (Invitrogen, Cat No. 15596-018). Extracted RNA was reverse transcribed to cDNA using QuantiTect reverse transcription kit (Qiagen, Cat No. 205311) as described by the manufacturer using Applied Biosytems Veriti Thermal Cycler. Real-time quantitative PCR was performed in an Eppendorf Mastercycler realplex using KiCqStart SYBR Green qPCR ReadyMix (Sigma-Aldrich, Cat No. KCQS00) per manufacturer's protocol. Data were analyzed for expression relative to 18S rRNA using the comparative Ct method. Forward and reverse qPCR primers for EWS-FLI1 were CAGCCTCCCACTAGTTACCC and GTTGAGGCCAGAATTCATG, respectively.

### Model #3^Col1a2.3Cre-EF and Col1a3.6Cre-EF^ and Model #11^Prx1Cre-EF^

*Ews-FLI1*KI mice (C57BL/6 background) had been described earlier [16]. *Col1a2.3-Cre* and *Col1a3.6-Cre* mice were generated previously [43] and *Prx1-Cre* mouse was purchased from Jackson Laboratory. *Ews-FLI1*KI mice were crossed with *Col1a2.3-Cre*, *Col1a3.6-Cre* or *Prx1-Cre* and pups were genotyped at weaning and at birth (n>100 for each cross). No viable pups carrying both the *EWS-FLI*KI and *Cre* alleles were obtained in any of the crosses.

### Models #4^Cosco-EF^, #5^Pgk-EF^, #6^Nse-EF and Nse-EF-SV^, #7^NEFL-EF^, #8^MT-EF^, #9^PLAPtTA-EF^, and #10^COMET and COMETΔNeo^

#### Generation of the targeting vector and COMET mouse

The 6158 bp COMET cassette (Supplementary Figure S16) was gene synthetized (EpochBiolabs, INC. Missouri city, TX) and cloned into Sma1 digested pUC19. The resulting pUC19-COMET plasmid was digested with Nhe1, the resulting COMET cassette was subcloned into Xba1 digested pROSA26-1 vector (gift from P. Soriano). The resulting pROSA26-1-COMET plasmid was linearized using SacII and purified. This targeting construct was electroporated into CK35 (129 Stevens Pasteur background) mouse embryonic stem cells for homologous recombination. G418-resistant clones were isolated, screened by PCR for homologous recombination, and confirmed by southern blot (150 bp EcoR1/HindIII fragment of pROSA-5’, gift from P. Soriano). Two positive embryonic stem cell clones (2C12, 2D8) were injected into C57BL/6J blastocysts to generate the first generation of chimeras (Model #10^COMET^). FRT flanked Neomycin selection cassette was deleted *in vitro* by transient transfection of pCAGGS-Flpe (Gene Bridges GmbH, Heidelberg, Germany) plasmid into 2C12, 2D8 ES clone according to manufacturer protocol. The deleted subclones 2A3 (derived from 2C12) and 1C3 (derived from 2D8) were injected into C57BL/6J blastocysts to generate the second generation of chimeras (Model #10^COMETΔNeo^).

#### Quantitative RT-PCR

cDNAs were synthesized from 1µg of RNA using the GeneAmp RNA PCR core Kit (Applied Biosystem, Courtaboeuf, France). Quantitative PCR analyses were performed using SYBR green (Applied Biosystem). The following primers were used: EWS-FLI1 (5’-GCCAAGCTCCAAGTCAATATAGC-3’, 5’-GAGGCCAGAATTCATGTTATTGC-3’); luciferase (5’-TTACACGGCGATCTTTCCGCCC-3’, 5’-AGTTGCGCGGAGGAGTTGTGTT-3’). Reactions were run on an ABI/PRISM 7500 (Applied Biosystem) and analyzed using the 7500 system SDS software. For detection of splice variant between *Gt(ROSA)26Sor* transcripts and COMET transcript, the following primers were used (EWSR1-kozac-Rev 5’-TCCGTGGACGCCATGGTGAATT-3’, ROSA26-EX1-Fw 5’-CTGCCGGGGCCGCCTAAAGAA-3’) with Phusion High-Fidelity DNA polymerase (**Finnzymes,** Finland); 35 cycles of 98°C for 7 seconds, 60°C for 20 seconds, and 72°C for 30 seconds.

#### Luciferase assay

293T cells were plated into six well plates and grown in DMEM (Invitrogen, Cergy Pontoise, France) supplemented with 10% fetal calf serum. The day after, cells were CaCl_2_ transfected for six hours using 1µg of pUC19-COMET alone and/or pIRES-Cre (Cre recombinase cloned into Xma1-Not1 digested pIRES) and/or pTet-On Advanced (Takara Bio Europe/Clontech, France) and 20 ng of pREP7-Rluc, (kindly provided by Keji Zhao). Twenty-four hours post-transfection, doxycycline (0 to 1000 ng/ml) was added to the media. Forty-eight hours post-transfection, cells were lysed and assayed for luciferase activity using the dual luciferase reporter assay system (Promega, Charbonnieres-les-bain, France) according to the manufacturer's instructions. Firefly activity was normalized to Renilla luciferase activity to adjust differences in transfection efficiency.

#### Antibodies

β-actin antibody (A-5316) was purchased from Sigma-Aldrich (Saint-Quentin Fallavier, France). Anti-FLI1 antibody (7.3) was described previously [102].

#### Noninvasive bioluminescence imaging

Approximately twenty minutes prior imaging, mice were injected intraperitoneally with 10 µl/g of body weight of sterile D-luciferin PBS solution (15 mg/ml). Mice were imaged using an IVIS Spectrum system (Caliper Life Sciences S.A., Villepinte, France) for 1 minute. Anesthesia was administered in an induction chamber with 2.5% isoflurane and reduced to 1.5% while the animal was in the IVIS imaging device. The data are reported as radiance (photons/sec/cm2/steradian).

### Model #12^Cre-TL-EF^

#### Mice

Generation of *Ews*loxpurolox/wt, *Fli1*loxhygrolox/wt and EWS-FLI1-V5 mice was carried out using standard gene targeting protocols. Correctly targeted embryonic stem cells were injected into albino C57BL/6 blastocysts by the Transgenic Facility at Stanford University. Southern blot analyses on embryonic stem cell clones were performed following standard protocols. *Sox9-Cre*, *Dermo1-Cre*, *P0*-*Cre* and *Col1*α2-*Cre* mice were obtained from researchers that made the mice. *Prx1-Cre* mice were obtained from the Jackson Laboratory (B6.Cg-Tg(Prrx1-cre)1Cjt/J Stock Number: 005584). *CMV-Cre* mice were obtained from the Jackson Laboratory (B6.C-Tg(CMV-cre)1Cgn/J Stock Number: 006054). *Ink4a/Arf* floxed mice were a kind gift from Dr. Ron DePinho [103]. At the end of the tumor study, mice were euthanized and a necropsy was carried out. Gross abnormalities by eye were noted and chunks of tumors were collected and snap frozen in liquid nitrogen for future study. Tumors chunks were crushed cold using a mortar and pestle chilled by liquid nitrogen and the ground tissue was resuspended in Trizol.

#### Cell culture

MEFs were isolated as following standard procedures from E13.5 embryos and cultured in DMEM with 10% BGS and 100U/100ug/2 mM Pen/Strep/Glutamine (Invitrogen). Mouse bone progenitor cells were isolated as previously described and cultured in α-MEM supplemented with 20% fetal bovine serum (Omega Scientific) and 100U/100μg/2 mM Pen/Strep/Glutamine (Invitrogen) [104]. Adenoviral GFP or Cre infections were performed overnight in culture. Hematopoietic cells were isolated following standard protocols and immediately resuspended in TRIzol [105].

#### RNA analysis

RNA was isolated using TRIzol reagent (Invitrogen) following the manufacturer's specifications. cDNA was synthesized using either the DyNAmo cDNA synthesis kit (New England Biolabs, F470) or the Maxima First Strand cDNA synthesis kit (Thermo, K1642). qPCR was performed using SYBR Green (Applied Biosystems).

#### Primers

EWS lox genomic DNA TGGCCAGGCTATAAAACTACTTCCA TGCTGGGATGACTAGTTACAATTCC

FLI1 lox genomic DNA GTGACGGAGATCCCGAATTCTTTCC AACTGGGCCAGCCAACGCTTTC

EWS-FLI1 CAATATAGCCAACAGAGCAGCA GCTCCTCTCCTGACAGAGTCAT

GGGCAGCAGCCTCCTACTA TTCCATGCTCCTCTCCTGAC

HPRT TGACACTGGTAAAACAATGCA GGTCCTTTTCACCAGCAAGCT

Ink4a TGAGGCCGGATTTAGCTCTGCTC TCCGCTGCAGACAGACTTGCCAG

Arf TGAGGCCGGATTTAGCTCTGCTC CTTGGTCACTGTGAGGATTC

### Models #13^RetroLTR-EF^ and #14^piggyBac-EF^

BM-MSCs were obtained from 6-week-old BALB/c mice (Crea Japan), according to the previously described method [57]. Self-renewal activities of BM-MSCs were confirmed by *in vitro* clonogenic assays, and differentiation into multiple lineages were confirmed by induction toward osteogenic, chondrogenic, neuronal, adipogenic and myogenic differentiation [56]. A FLAG-tagged *EWS-FLI1* was subcloned into pMYs-ires-neo retroviral vector, and retroviral packaging was carried out using PLAT-E cells (a gift from T. Kitamura). BM-MSCs were then transduced with the *EWS-FLI1* retrovirus and injected from tail vein into irradiated BALB/c mice (model #13). *EWS-FLI1* was also subcloned into piggyBac transposon plasmid (a gift from P. Liu), and BM-MSCs were transfected with PB-EF and transposase mRNA generated by using an mMESSAGE mMACHINE T7 Ultra kit (Ambion). The cells were transplanted subcutaneously or injected *via* tail vein into irradiated recipients. Expression of EWS-FLI1 was confirmed by western blotting using anti-FLAG M2 and anti-beta-tubulin antibodies (Sigma).

### Model #15 ^CreEP-TL-EF^

Generation of *Ewsr1*fl/+*:Fli1*fl/+ mice (C57BL/6) was described elsewhere [30]. Genotypes of mice were confirmed by genomic PCR. The Cre expression vector pMC1-Cre (a gift by T. Yagi) was delivered by *in vivo* electroporation. The gastrocnemius muscles of 8-week-old mice were injected with 50 µg of plasmid DNA, and the site of inoculation was immediately given electric pulses with a CUY21EDIT Square Wave Electroporator (NEPAGENE) using a CUY560-3 electrode. The pulse was 50 ms in duration at a voltage of 50V. Cre expression was confirmed 2 weeks after electroporation by RT-PCR.

### Model #16^Ad5Cre-EF^

#### Mice

Both adenovirus-Cre (Ad5-CMV-Cre-eGFP) and adenovirus-eGFP (Ad5-CMV-eGFP) were purchased from University of Iowa Viral Vector Core Facility (Iowa city, IA). For intramuscular (IM) delivery *E/F*+/+ mice were injected with 20μl of 10^9^pfu Ad5-CMV-Cre-eGFP in the left leg and 20μl of 10^9^pfu Ad5-CMV-eGFP in the right leg at 1 day, 1 week, or 2 weeks of age using 31guage 3/8 inch Thinpro insulin syringe (Terumo). For IP delivery, *E/F*+/+ mice were injected with 20μl of 10^9^pfu Ad5-Cre at 1 day or 1 week of age. Littermate mouse of the same age and genotype were injected with 20μl of 10^9^pfu Ad5-eGFP as a control. For IV delivery, 20μl of 10^9^pfu Ad5-Cre was intravenously injected to 3 weeks old *E/F*+/+ mice. Their littermates were injected with 20μl of 10^9^pfu Ad5-eGFP as a control.

#### PCR

DNA extracted from *E/F*+/- and *E/F*+/+ mice livers were used as negative controls while DNA from leukemic *E/F*+/- *Mx1-Cre*+ mouse liver was used as a positive control. DNAs extracted from muscles of *E/F*+/+ mice injected with Ad5-eGFP in the right legs were also used as negative controls. DNA was isolated using wizard genomic DNA purification kit per manufacturer's protocol (Promega, A1120) Extracted DNAs were subjected to PCR to confirm that Cre-mediated recombination has occurred. A forward primer located in the endogenous Rosa26 locus (GATCCACTAGTTCTAGAGCGGC) and a reverse primer that lies shortly after the first loxP site (GAGTTGTTATCAGTAAGGGAGC) were used to detect the knock-in allele. On the hand, the same forward primer in combination with a reverse primer that lies within the EWS-FLI1 sequence (GGTATCATAAGCACCAGTG) were used to detect the removal of the STOP-cassette.

The generation of this model was supported by charity funds collected by St. Anna Kinderkrebsforschung e.V. and from support from the Deutsche Forschungsgemeinschaft Collaborative Research Centre 1149 ‘Trauma’ (INST 40/492-1), “Immunobone” (SPP 1468 Tu220/6-2). We thank Dr. S. Baker for providing E/F mice.

### Models #2^OsxCre-EF^ and #16^Ad5Cre-EF^

Support for this work came from the Children's Cancer Foundation (Baltimore MD), St. Baldrick's Foundation, Go4theGoal, a Burroughs Wellcome Clinical Scientist Award in Translational Research (J.T.), and the NIH RC4CA156509 (J.T.), R01CA133662 (J.T.), R01CA138212 (J.T.). We wish to thank the Flow Cytometry/Cell sorting and Histopathology & Tissue Shared Resource (HTSR) Shared Resources at the Lombardi Comprehensive Cancer Center (Georgetown University), which are supported by a grant P30 CA51008 (PI Louis Weiner) from the National Cancer Institute. In addition, we would like to thank Kelli Schanze for her excellent technical support.

### Models #4^Cosco-EF^, #5^Pgk-EF^, #6^Nse-EF and Nse-EF-SV^, #7^NEFL-EF^, #8^MT-EF^, #9^PLAPtTA-EF^, and #10^COMET and COMETΔNeo^

This work was supported by grants from the Institut National de la Santé et de la Recherche Médicale, the Institut Curie, the Institut National du Cancer, the Ligue Nationale contre le Cancer (Equipe labellisée and CIT program), the Réseau National des Génopoles, Agence National de la Recherche, the société Française des Cancers de l’Enfant, and the following associations: Courir pour Mathieu, Dans les pas du Géant, Olivier Chape, Les Bagouzamanon and les Amis de Claire.

### Model #12^Cre-TL-EF^

We thank Dr. Charles Chan and Dr. Irving Weissman for helpful discussions. We especially thank Dr. Charles Chan for teaching us to isolate embryonic mouse bone progenitors, for reagents and for FACS training. We thank Dr. Hong Zeng and the rest of the members of the Transgenic Facility at Stanford University for help in generating the chimeras and discussions about ES targeting. We thank Dr. Ronald DePinho for the conditional Ink4a/Arf mice. We thank Dr. David Ornitz for the *Dermo1*-*Cre* mice. We thank Dr. Mohan Subburaman and Dr. Peter Angel for the *Col1*a2*-Cre* mice. We thank Dr. Benoit de Crombrugghe for the *Sox9-Cre* mice. We thank Dr. Joe Kissil for the *P0-Cre* mice.

## SUPPLEMENTARY MATERIALS FIGURES AND TABLES


