# Supplementary material for: Combined experience of six independent laboratories attempting to create an Ewing sarcoma mouse model
Source: Oncotarget. 2016 May 15;8(21):34141–63. doi: 10.18632/oncotarget.9388 (PMC5470957; doi:10.18632/oncotarget.9388)
Supplement: Supplementary file 1 [file oncotarget-08-34141-s001.pdf]

Combined experience of six independent laboratories attempting to create an Ewing sarcoma mouse model

Supplementary Material

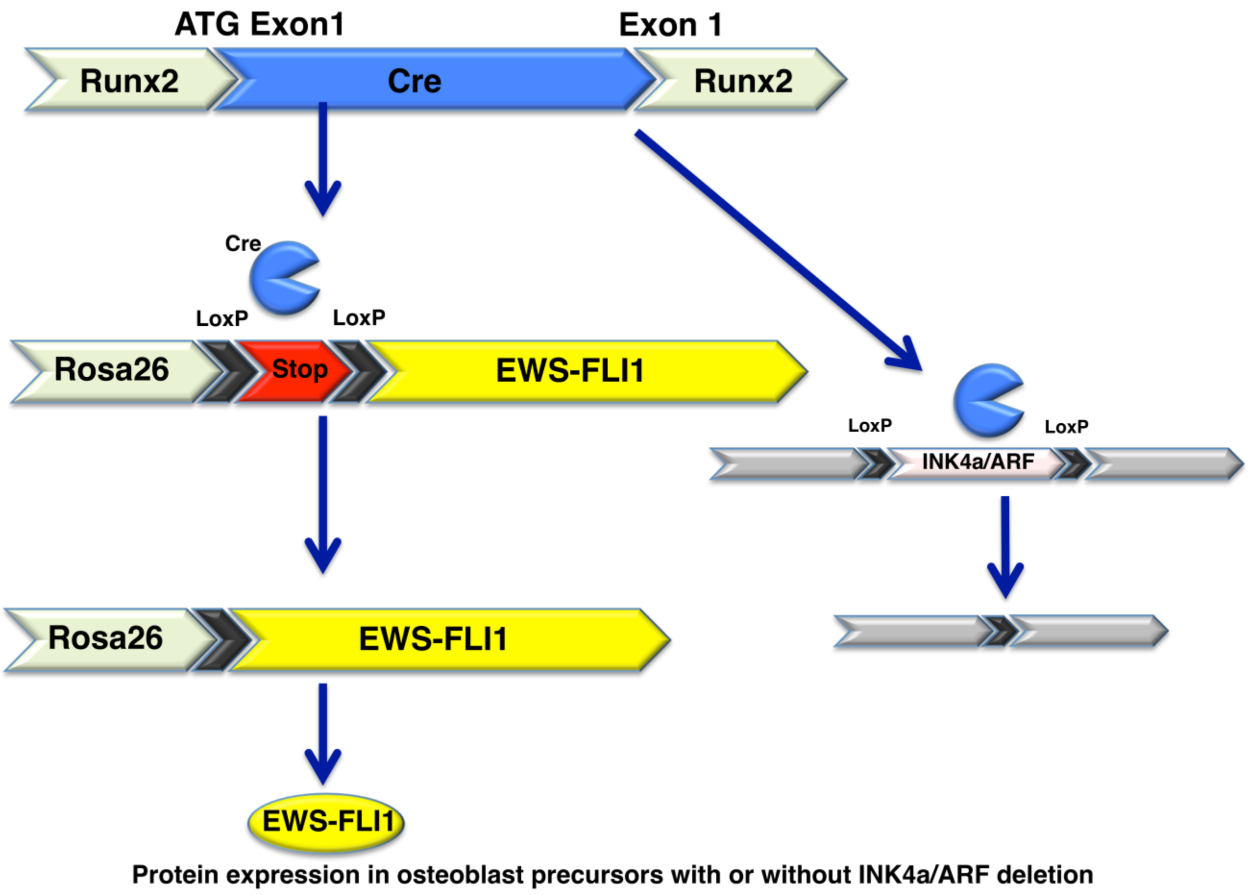

Supplementary Figure S1: (Model #1$^{Runx2Cre\text{-}EF}$): A schematic illustration of Model #1$^{Runx2Cre\text{-}EF}$.

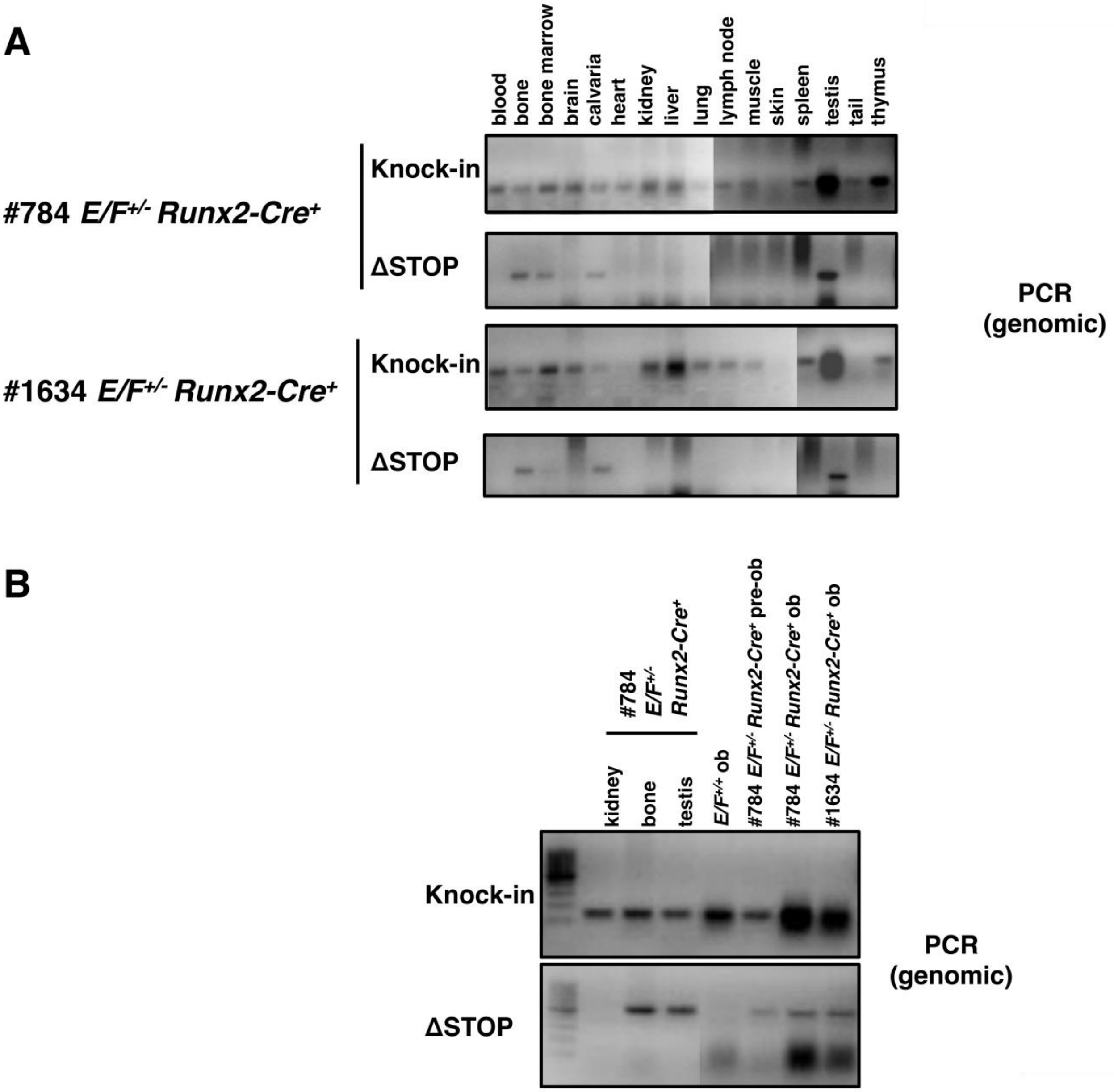

Supplementary Figure S2: (Model #1$^{\text{Runx2Cre-EF}}$):

(**A**) The excision of the STOP-cassette in different organs was confirmed by genomic PCR. (**B**) Different organs and premature (pre-ob) and mature osteoblasts (ob) were isolated from different *E/F*$^{+/-}$ *Runx2-Cre*$^{+}$ mouse lines and subjected to genomic PCR to determine the presence of the *E/F* transgene and the STOP-cassette deletion.

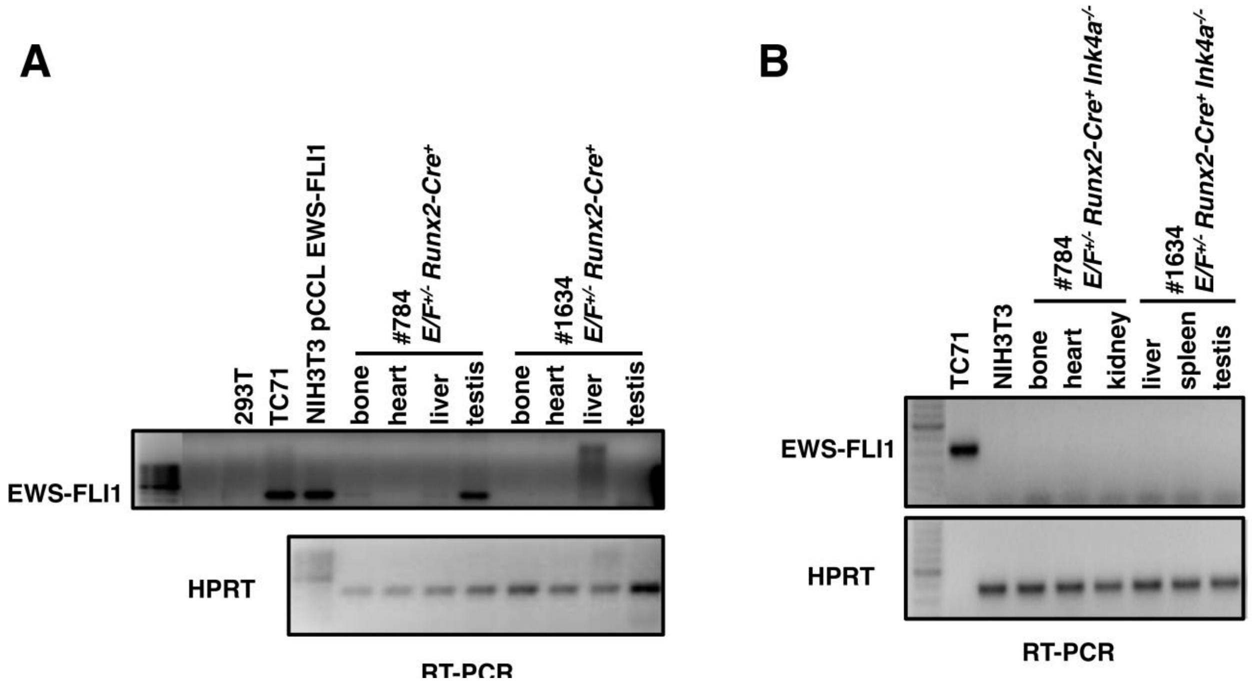

Supplementary Figure S3: (Model #1$^{\text{Runx2Cre-EF}}$):

Expression of EWS-FLI1 mRNA in multiple tissues of *E/F*$^{+/-}$ *Runx2-Cre*$^{+}$ mice with (**A**) and without (**B**) *Ink4a* knockout was evaluated by RT-PCR. EWS-FLI1 expression could not be detected in different organs except for the testis in just one mouse line. 293T cells served as the negative control, and NIH3T3 cells transfected with pCCL EWS-FLI1 and TC71 served as the positive control for EWS-FLI1 expression.

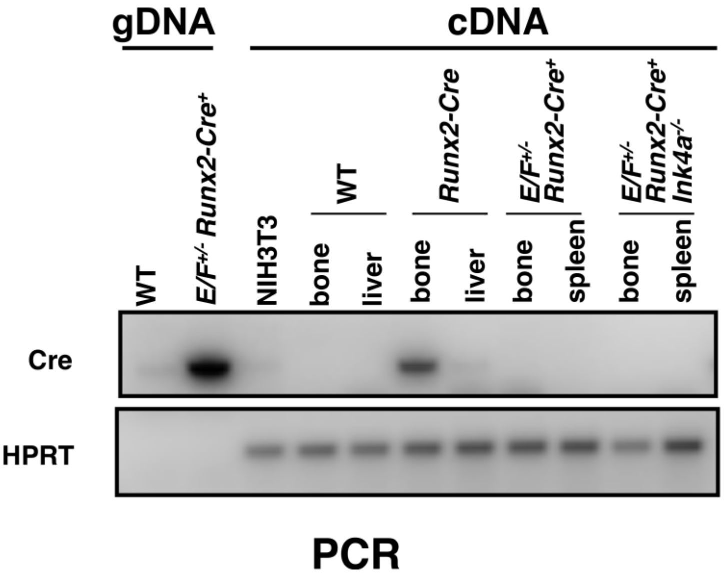

Supplementary Figure S4: (Model #1$^{\text{Runx2Cre-EF}}$):

Runx2-Cre was only expressed in mice without EWS-FLI1 expression. (**A**) The presence of the *Runx2-Cre* transgene in genomic DNA was detectable, but there was no *Cre* mRNA expression in mice with *EWS-FLI1* transgene, suggesting that cells expressing *Runx2-Cre* and *EWS-FLI1* have a growth disadvantage. *Cre* RNA was detected from reverse-transcribed cDNA with primers specific for the *Cre* recombinase. gDNA from wild-type (WT) and #784 *E/F*$^{+/-}$ *Runx2-Cre*$^{+}$ mice were used as negative and positive controls for the presence of Cre DNA, respectively. The presence of the *Cre* transcript could only be verified in #784 *Runx2-Cre* mice but not in #784 *E/F*$^{+/-}$ *Runx2-Cre*$^{+}$ and #784 *E/F*$^{+/-}$ *Runx2-Cre*$^{+}$ *Ink4a*$^{-/-}$ mice. mHPRT was used to ensure proper cDNA transcription. The primers specific for mHPRT were designed in two different exons so that only mRNA, but not the genomic DNA, was recognized.

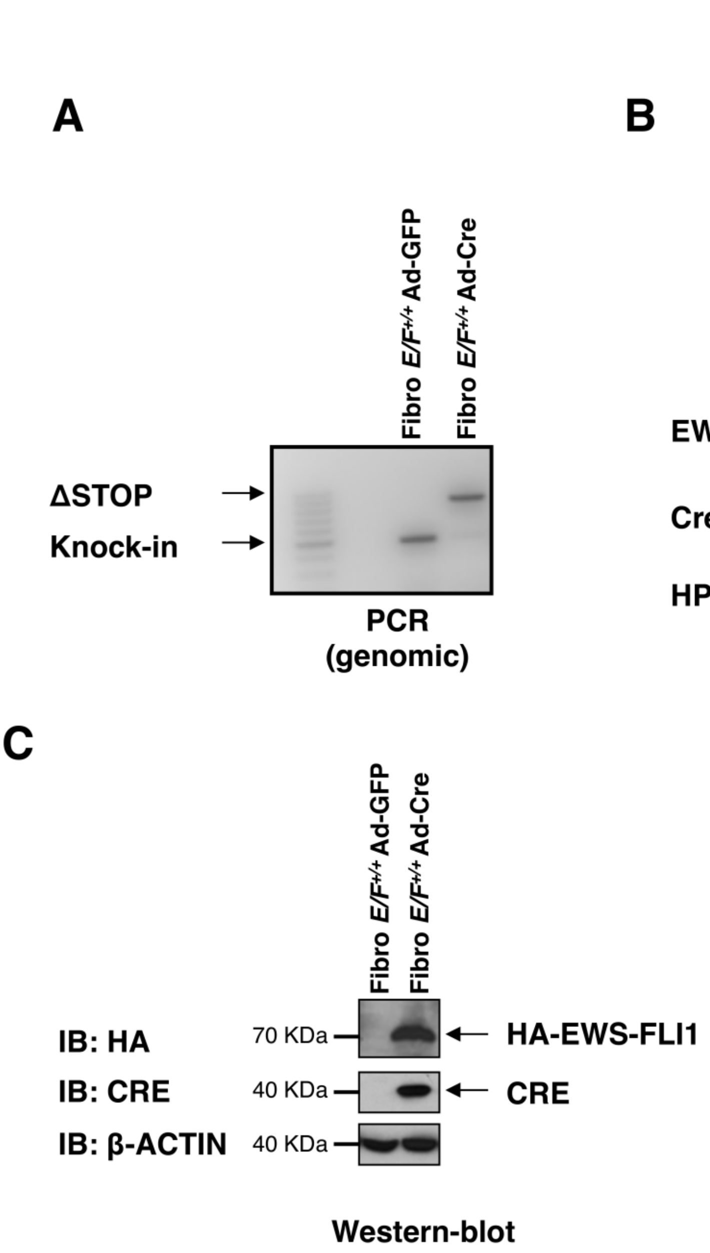

Supplementary Figure S5: (Model #1$^{Runx2Cre-EF}$):

EWS-FLI1 could be expressed from the *Rosa26* locus *in vitro*. Ear skin fibroblasts isolated from *E/F* mice were transduced with Cre-expressing adenovirus. GFP adenovirus was used as a negative control. Cre-mediated recombination in genomic DNA was confirmed by PCR (**A**). EWS-FLI1 and Cre expression was confirmed at the mRNA (**B**) and protein (**C**) levels.

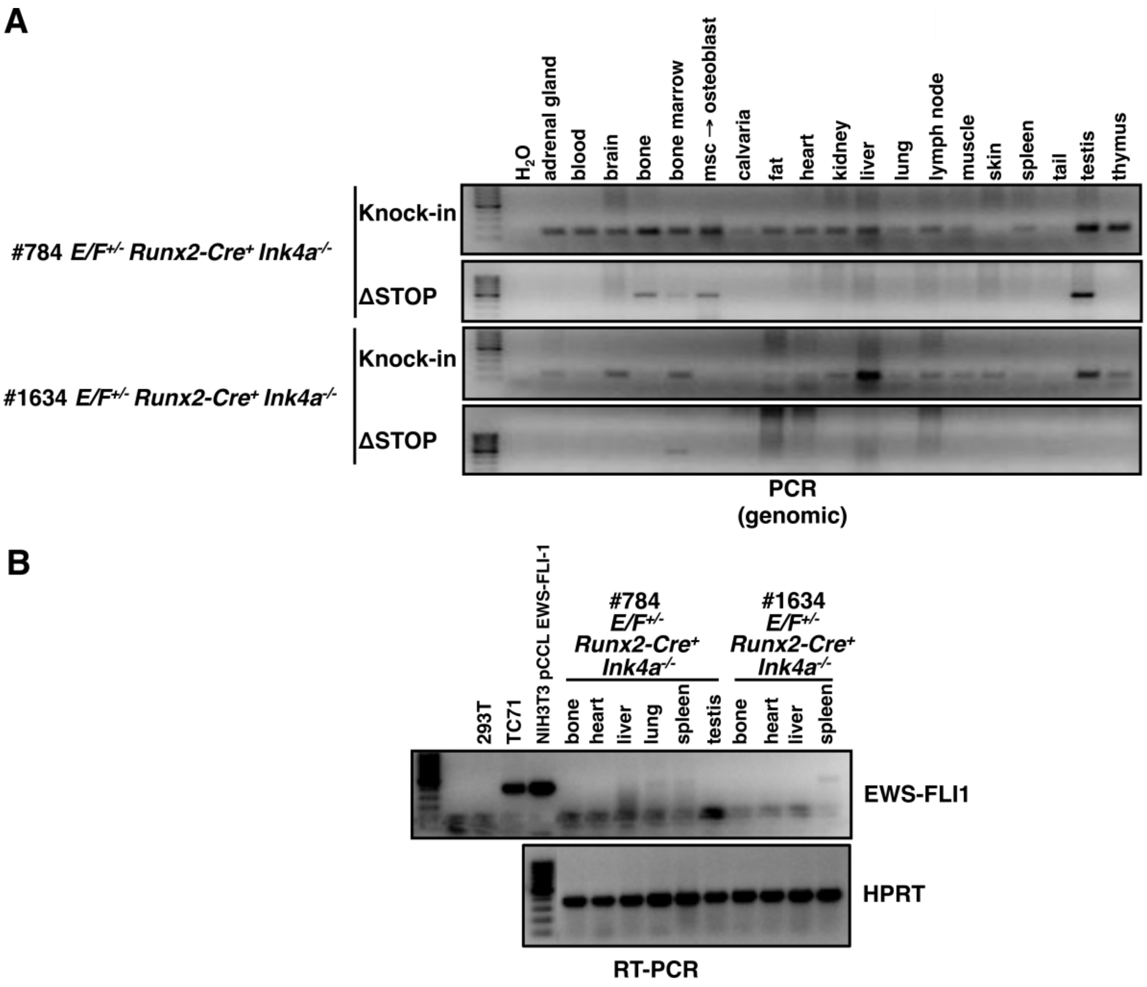

Supplementary Figure S6: (Model #1$^{\text{Runx2Cre-EF}}$):

(A) Genomic DNA isolated from several different tissues of #784 and #1634 *E/F*$^{+/-}$ *Runx2-Cre*$^{+}$ *Ink4a*$^{-/-}$ mice was analyzed for deletion of the STOP-cassette (ΔSTOP) using PCR. The existence of the knock-in allele of *E/F* was used as a control to ensure the presence of the transgene in genomic DNA. (**B)** *EWS-FLI1* RNA was detected with specific primers from reverse-transcribed cDNA but could not be observed in the tissues of #784 *and* #1634 *E/F*$^{+/-}$ *Runx2-Cre*$^{+}$ *Ink4a*$^{-/-}$ mice. *Hprt* was used to ensure proper cDNA transcription. The human TC71 cell line showed no *Hprt* band because the primers were specific for the mouse homologue. The 293T cell line was used as a negative control for EWS-FLI1 expression; the TC71 ES cell line was used as a positive control for EWS-FLI1 expression.

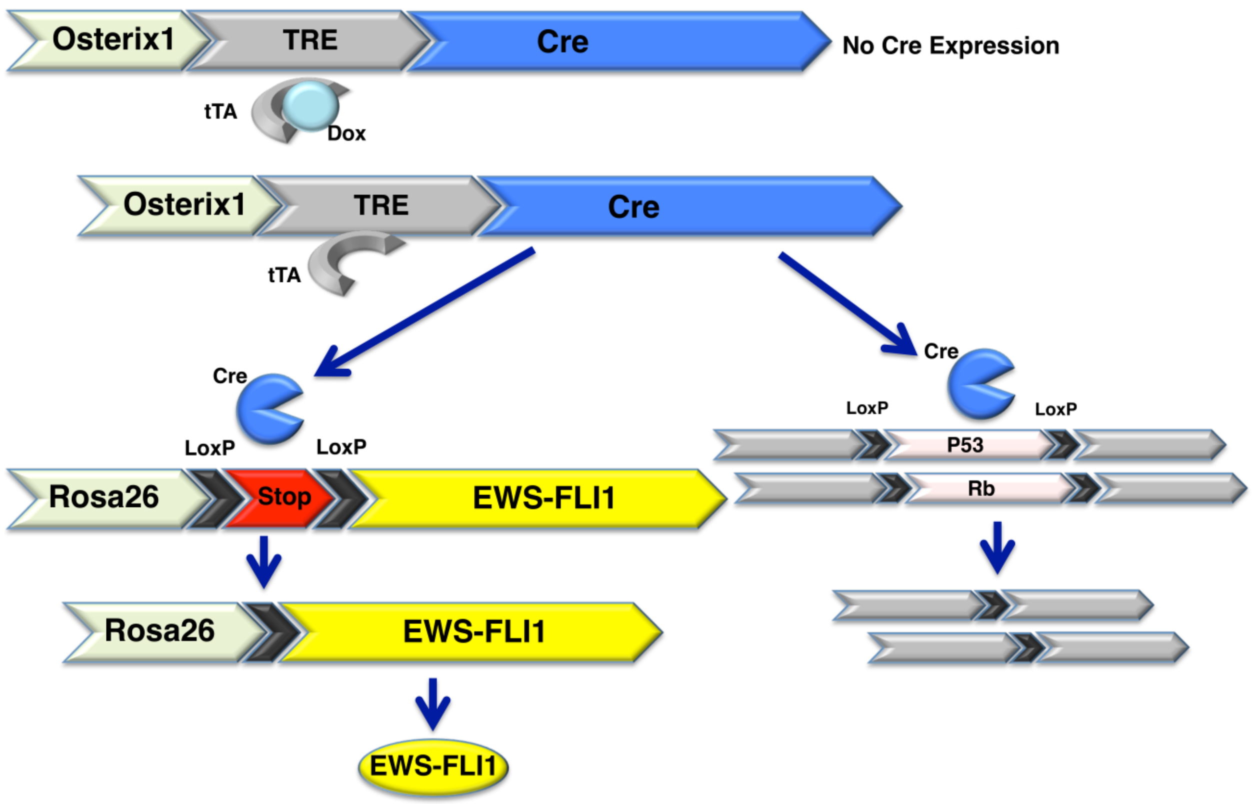

Supplementary Figure S7: (Model #2$^{\text{OsxCre-EF}}$):

Schematic demonstrating the anticipated active *EWS-FLI1* allele produced upon Cre-mediated recombination between loxP sites and subsequent removal of the STOP-cassette in conjunction with or without *p53* and *pRb* heterozygous deletion in osteoblast precursor cells. Cre was expressed under the Tet-Off-based *Osterix* promoter.

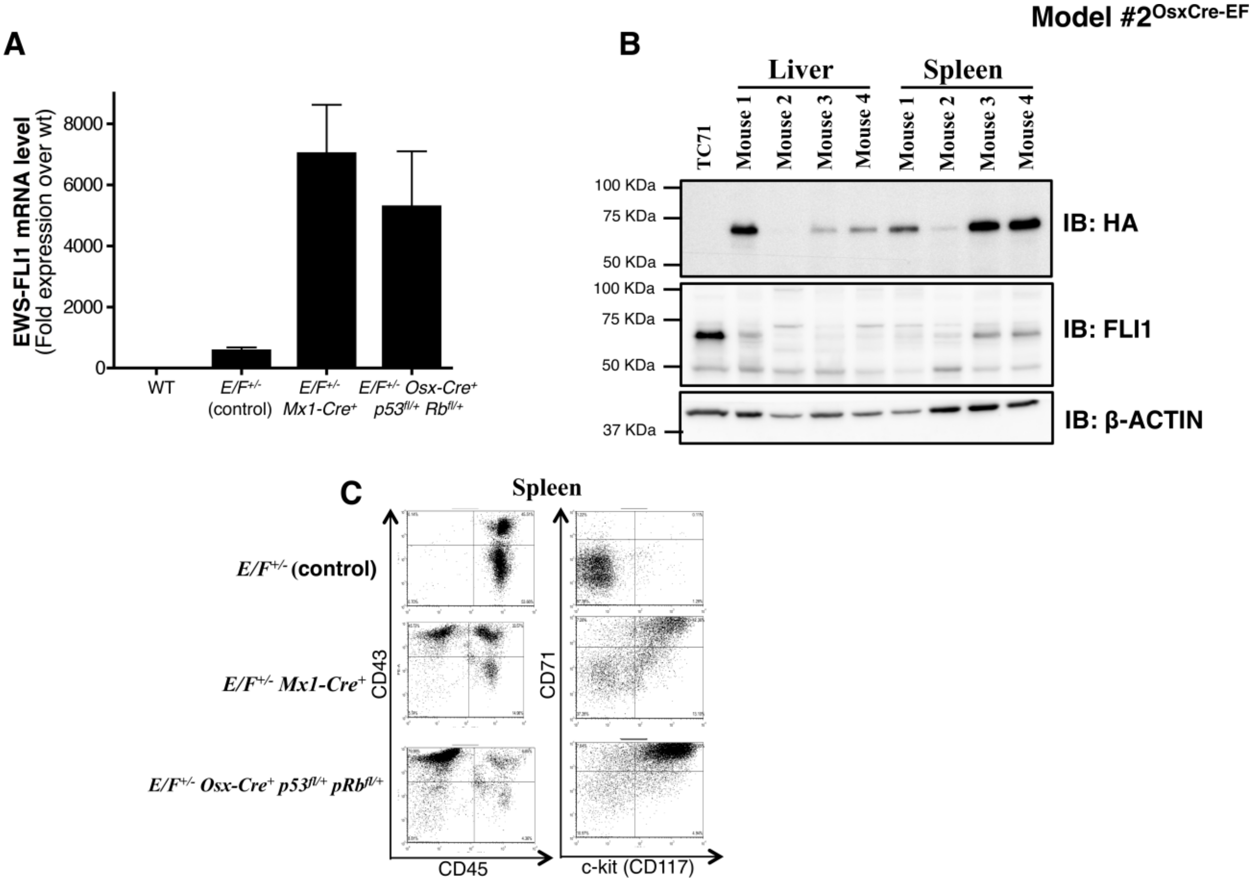

Supplementary Figure S8: (Model #2$^{OsxCre\text{-}EF}$):

Analysis of the expression of EWS-FLI1 at the mRNA **(A)** and protein **(B)** levels in the liver and spleen of *E/F*$^{+/-}$ *Osx-Cre*$^{+}$ *p53*$^{fl/+}$ *pRb*$^{fl/+}$ mice by RT-qPCR and western blot. EWS-FLI1 was detected in spleen lysates using an FLI1 antibody and anti-hemagglutinin (HA) antibody, as EWS-FLI1 was tagged with an HA epitope. Lysate from the ES cell line TC71 was used as a control for EWS-FLI1. **(C)** Immunophenotyping of cells from the spleens of mice with EWS-FLI1-induced leukemia in *E/F*$^{+/-}$ *Osx-Cre*$^{+}$ *p53*$^{fl/+}$ *pRb*$^{fl/+}$ vs. *E/F*$^{+/-}$ *Mx1-Cre*$^{+}$ vs. *E/F*$^{+/-}$ *p53*$^{fl/+}$ *pRb*$^{fl/+}$ control mice. Spleen cells were stained with fluorescently labeled CD71, CD43, CD117, and CD45 antibodies and were analyzed using FACS.

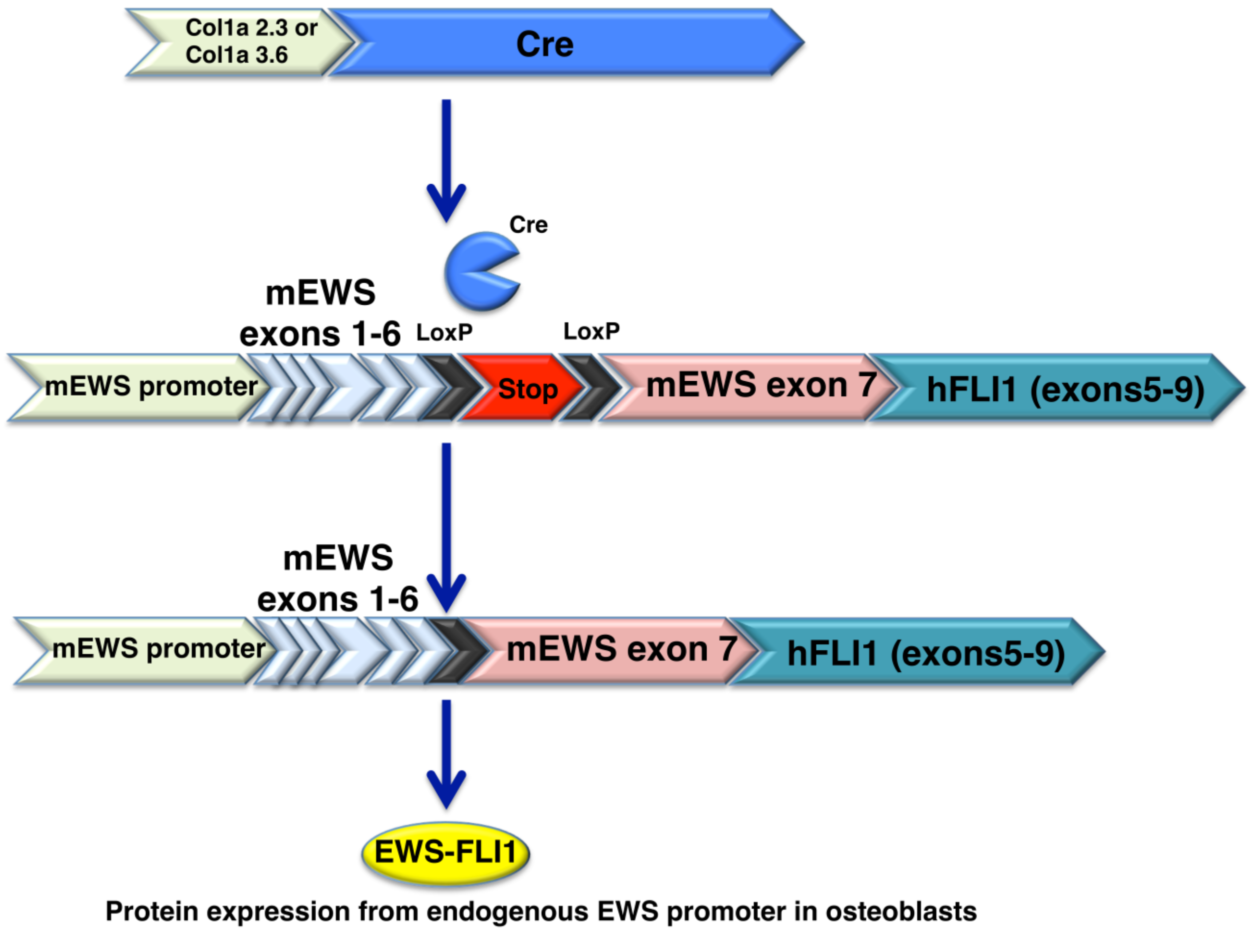

Supplementary Figure S9: (Model #3$^{\text{Col1a2.3Cre-EF and Col1a3.6Cre-EF}}$):

A schematic illustration of Model #3$^{\text{Col1a2.3Cre-EF and Col1a3.6Cre-EF}}$. EWS-FLI1 expression was designed to be driven by the endogenous *Ews* promoter in bone.

Model #4$^{Cosco\text{-}EF}$

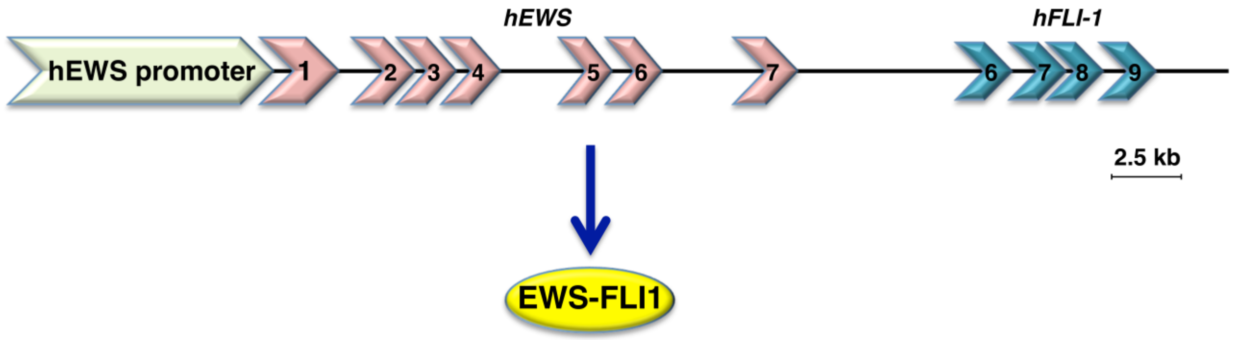

Ubiquitous protein expression from human EWS promoter.

Supplementary Figure S10: (Model #4$^{Cosco\text{-}EF}$): Generation of the Model #4$^{Cosco\text{-}EF}$.

A cosmid containing the full *EWS-FLI1* genomic fusion and a neomycin resistance cassette (45 kb) was co-transfected with *pB17* (containing a *PGK* intron to facilitate the insertion of the construct) into CGR8 mouse embryonic stem cells. Eighteen out of 144 neomycin-resistant clones were positive by PCR screening for *EWS-FLI1*. A clone was used to generate 44 chimeric mice, but none of these were transgenic.

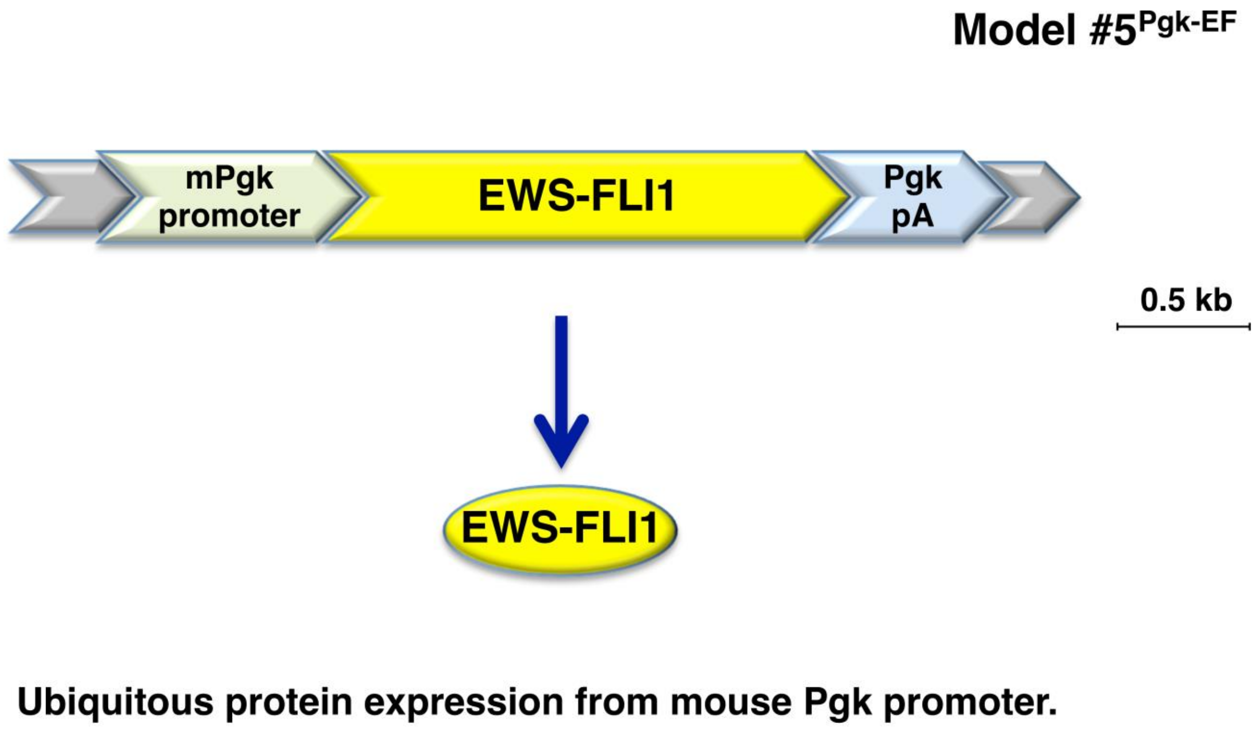

Supplementary Figure S11: (Model #5$^{Pgk\text{-}EF}$): Generation of the Model #5$^{Pgk\text{-}EF}$.

The *EWS-FLI1* coding sequence was cloned upstream of the murine (-794 +317) *phosphoglucokinase* (*Pgk*) promoter that was cloned into the pUC19 plasmid. This construct was microinjected to generate transgenic mice. Two out of 16 F0 mice were transgenic, but none of the F1 progeny were positive.

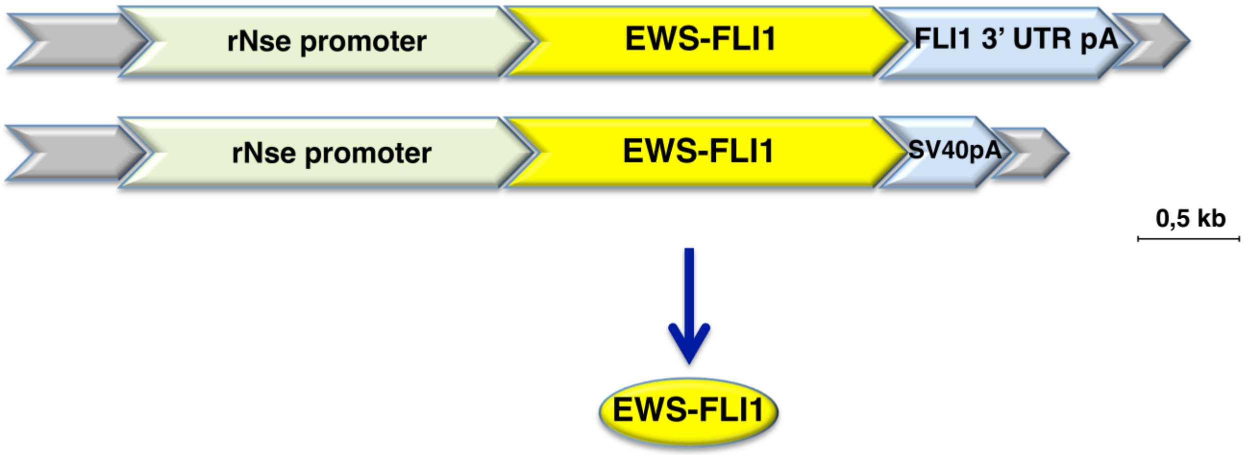

Protein expression from Nse promoter in neuronal tissues.

Supplementary Figure S12: (Model #$6^{\text{Nse-EF and Nse-EF-SV}}$): Generation of the Model #$6^{\text{Nse-EF and Nse-EF-SV}}$.

The 1.8kb rat *neuron specific enolase* (*Nse*) promoter [106] was cloned into the pbluescript SK (+) plasmid. For the *Nse-E/F* construct, the *EWS-FLI1* coding sequence and the full *FLI1* 3'UTR were cloned upstream of the promoter. For the *Nse-E/F-SV* construct, the *EWS-FLI1* coding sequence was cloned upstream of the promoter, and the *SV40* polyadenylation sequence was cloned downstream of *EWS-FLI1*. Both constructs were co-transfected with pB17 (containing a *PGK* intron to facilitate the insertion of the construct) into CGR8 mouse embryonic stem cells. Twenty-one of 120 neomycin-resistant clones were positive by PCR screening for *EWS-FLI1* integration, and one positive clone for each construct was used to generate chimeras. Only one *Nse-E/F* chimera contained the transgene, but no stable mouse strain could be obtained.

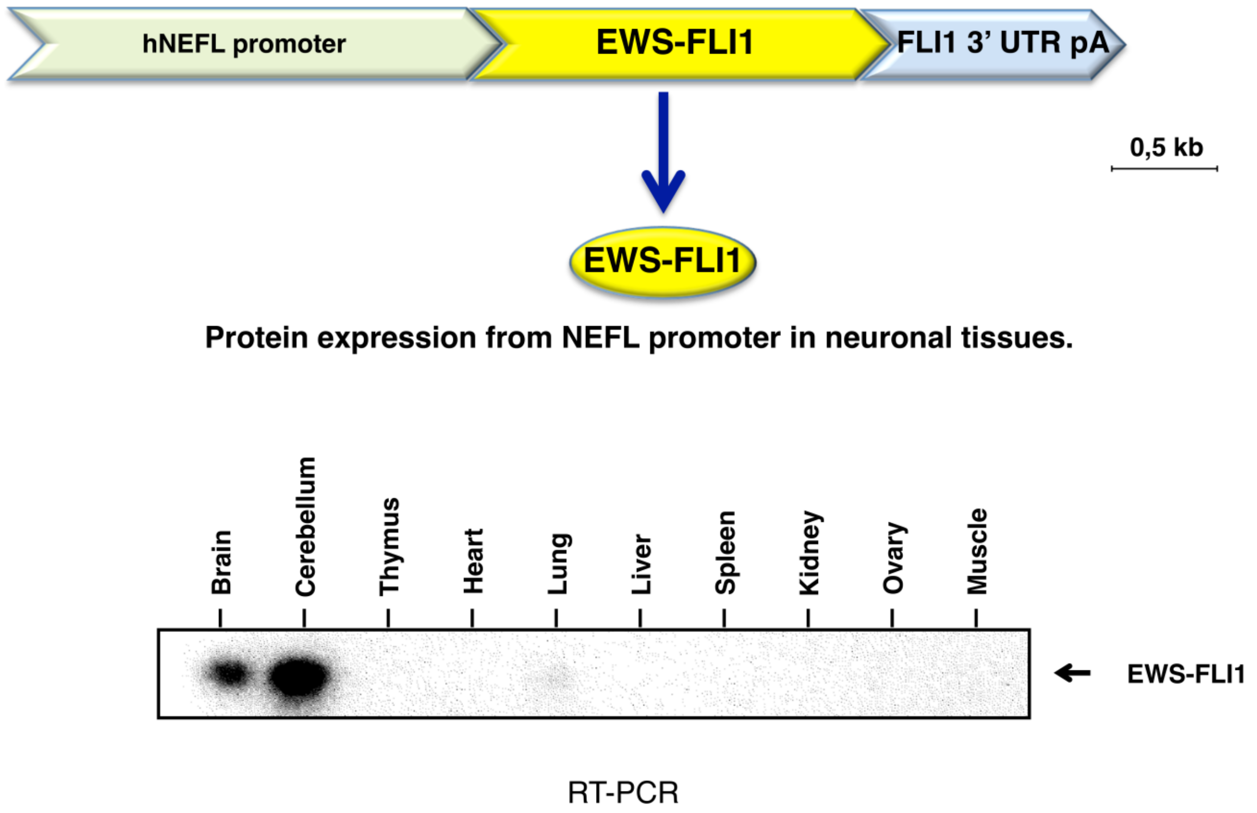

Supplementary Figure S13: (Model #7$^{NEFL\text{-}EF}$): Generation of Model #7$^{NEFL\text{-}EF}$.

The -1416 +15 human *neurofilament light* (*NEFL*) promoter was cloned into the pbluescript SK (+) plasmid. The *EWS-FLI1* coding sequence and the full *FLI1* 3'UTR were cloned upstream of this promoter. This construct was microinjected to generate transgenic mice. Ten founders were obtained, but only five of ten gave transgenic progeny upon backcrossing. Mendelian inheritance was observed for several generations in four of five transgenic mouse lines. Agarose electrophoresis for the *EWS-FLI1* RT-PCR product from various organs of a representative adult *NEFL-E/F* mouse is shown at the bottom.

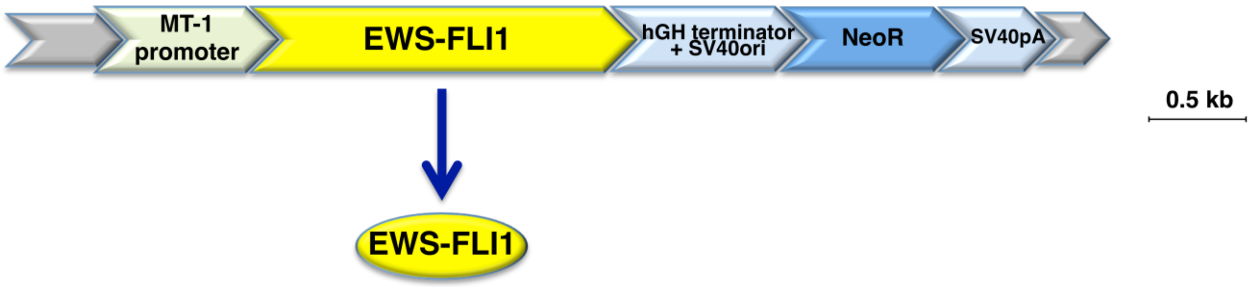

$ZnCl_2$ inducible ubiquitous protein expression from MT-1 promoter.

Supplementary Figure S14: (Model #8$^{MT\text{-}EF}$): Details for the generation of the Model #8$^{MT\text{-}EF}$. The *EWS-FLI1* coding sequence was cloned into the pMTCB6+ plasmid upstream of the zinc-inducible *metallothionein* promoter. The Pvu1 linearized fragment was used to generate four transgenic mouse strains.

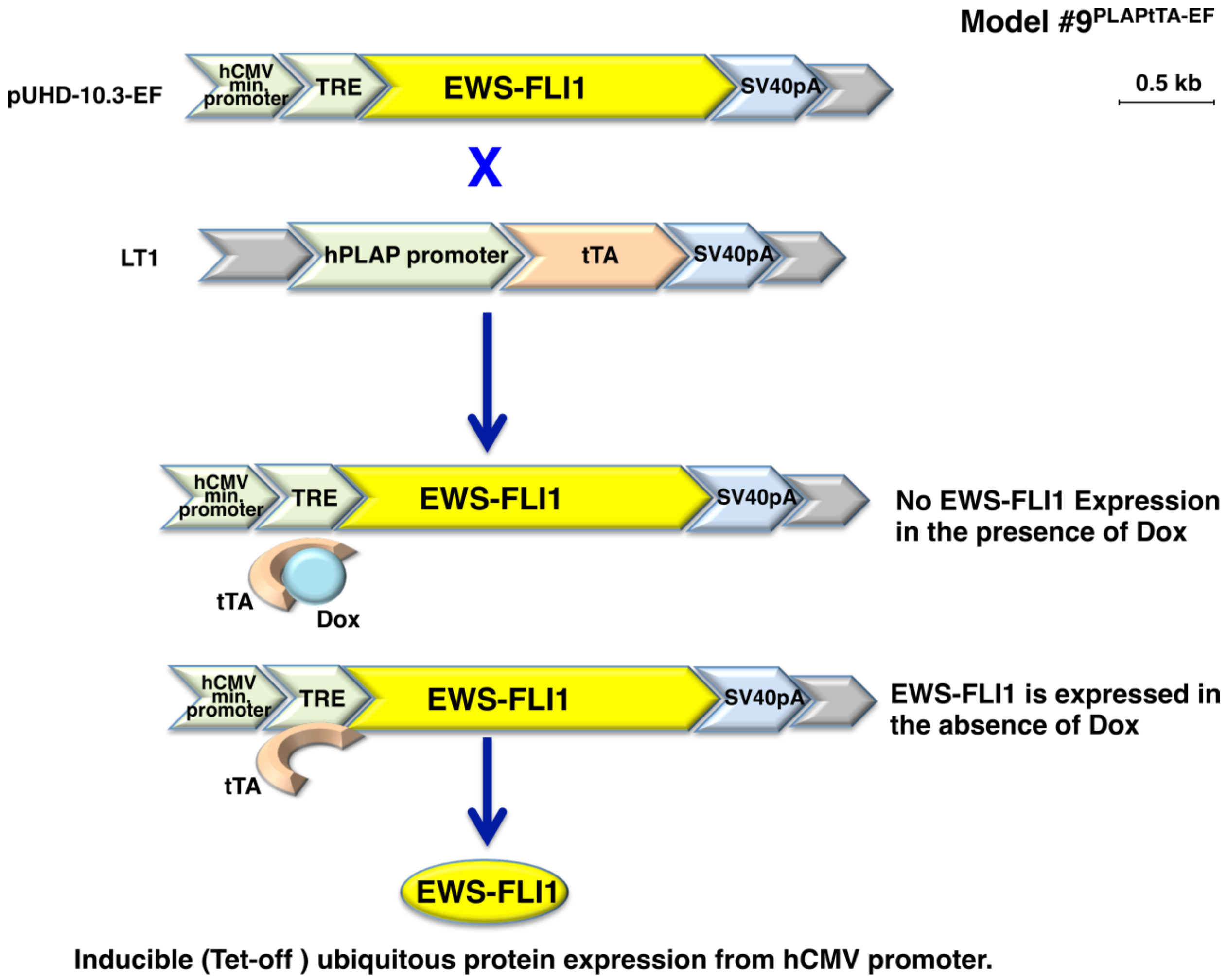

Supplementary Figure S15: (Model #9$^{\text{PLAPtTA-EF}}$): Details for the generation of Model #9$^{\text{PLAPtTA-EF}}$.

The *EWS-FLI1* coding sequence was cloned upstream of the *hCMV* minimal promoter with heptamerized upstream tet-operators into the pUHD-10.3 plasmid. The Nar1-Xho1 linearized fragment was used to generate a transgenic line (5347) that was backcrossed with the LT1 strain (expressing the tetracycline-controlled transactivator (tTA) under the human *placental alkaline phosphatase* promoter). None of these backcrosses gave rise to double transgenic mice.

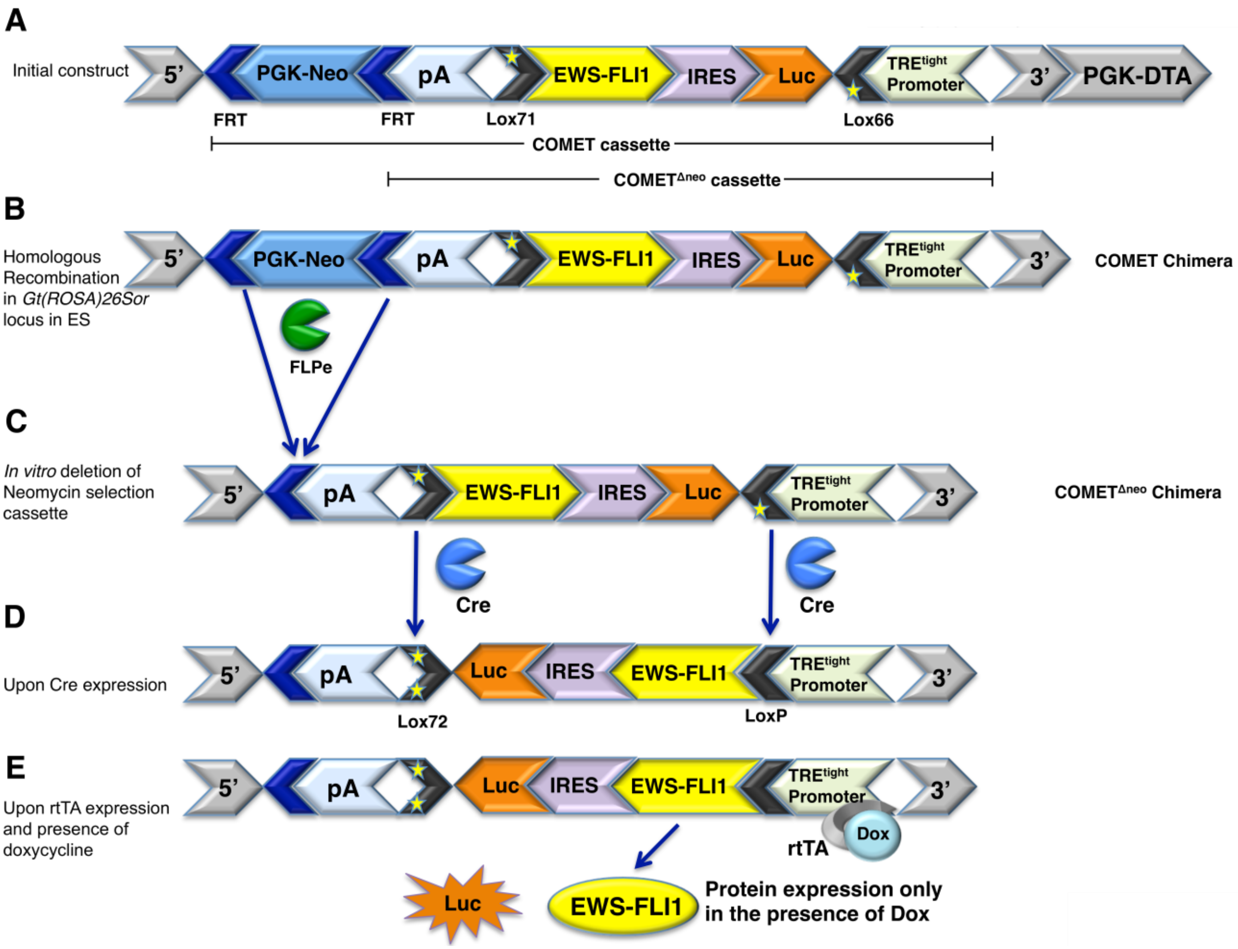

Supplementary Figure S16: (Model #10$^{\text{COMET and COMET}\Delta\text{Neo}}$): Strategy to express EWS-FLI1 in a conditional and inducible manner.

(A) The raw construct contains full-length *EWS-FLI1* followed by an *IRES Luciferase* (Luc) sequence. This cassette is flanked by modified *LoxP* sites (*Lox66* and *Lox71*) and is oriented antisense to the *TREtight* inducible promoter. The 6158 bp COMET cassette delimitation is shown. **(B)** Region homologous to the *Gt(ROSA)26Sor* locus. The positive (*neomycin*) and negative (*diphtheria toxin A)* selection cassettes allow for homologous recombination in murine embryonic stem cells. Model #10^{COMET} chimeric mice were generated from these embryonic stem cells. **(C)** Deletion of the *FRT* flanked *neomycin* selection cassette was performed *in vitro*

in embryonic stem cells by the transient expression of FLPe recombinase. Model #10$^{COMET\Delta Neo}$ chimeric mice were generated from these embryonic stem cells. **(D)** Upon expression of Cre recombinase, the *EWS-FLI1 IRES luciferase* cassette is flipped and oriented in sense with the *TREtight* inducible promoter. This recombination generates a *LoxP* site and a *Lox72* site that are no longer recognized by the Cre recombinase; therefore, the *EWS-FLI1 IRES luciferase* cassette remains locked in this orientation. **(E)** Upon the addition of doxycycline and expression of rtTA, this complex can then initiate transcription of the *EWS-FLI1 IRES luciferase* cassette, resulting in EWS-FLI1 and luciferase expression.

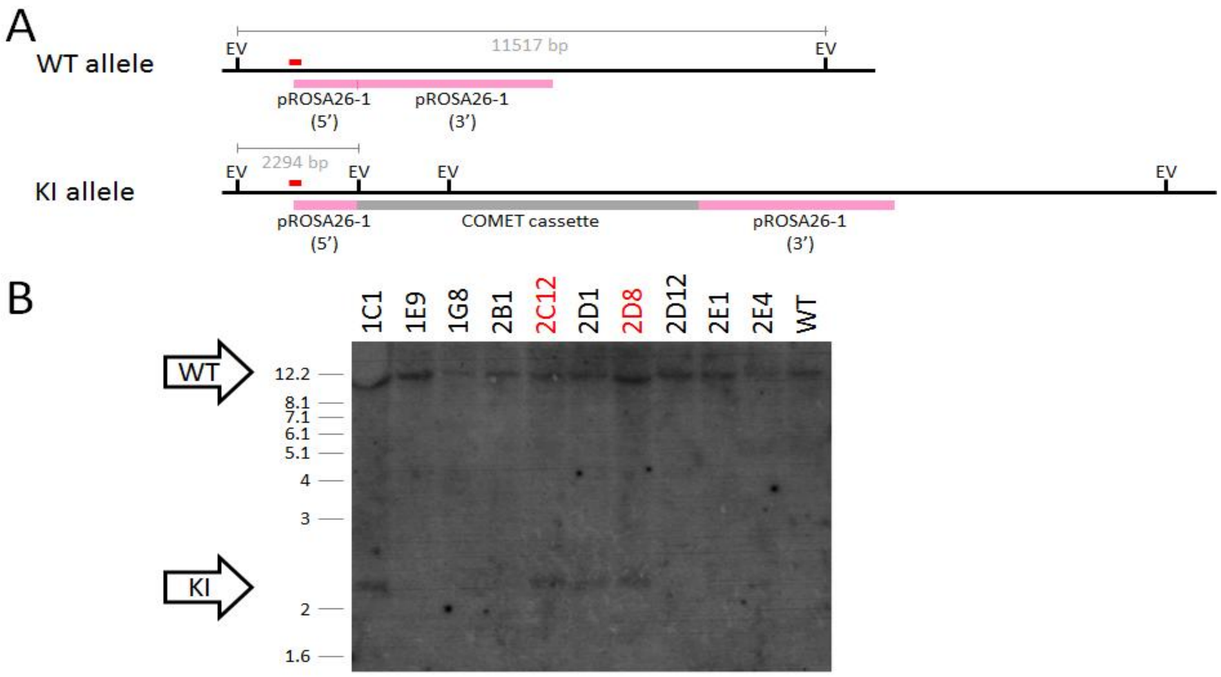

Supplementary Figure S17: Southern blotting for Model #10^{COMET} embryonic stem cell clones. **(A)** Scheme of the *Gt(ROSA)26Sor* locus in the wild-type (WT) allele and knock-in (KI) COMET allele. pRosa26-1 (5)' and pRosa26-1 (3)' regions (pink boxes) were used as homology regions for the homologous recombination to knock-in the COMET cassette (gray box). EcoRV restriction sites are shown (EV). A P^{32}-radiolabelled-150bp probe (EcoRI-HindIII fragment from pROSA26-5' plasmid, shown as red bar) was used for Southern blotting experiments using EcoRV-digested genomic DNA extracted from embryonic stem cell clones. **(B)** The Southern blot shows the expected hybridization at 2,294 bp for KI clones (1C1, 2C12, 2D1, 2D12). Clones 2C12 and 2D8 were used to generate Model #10^{COMET} chimeric mice.

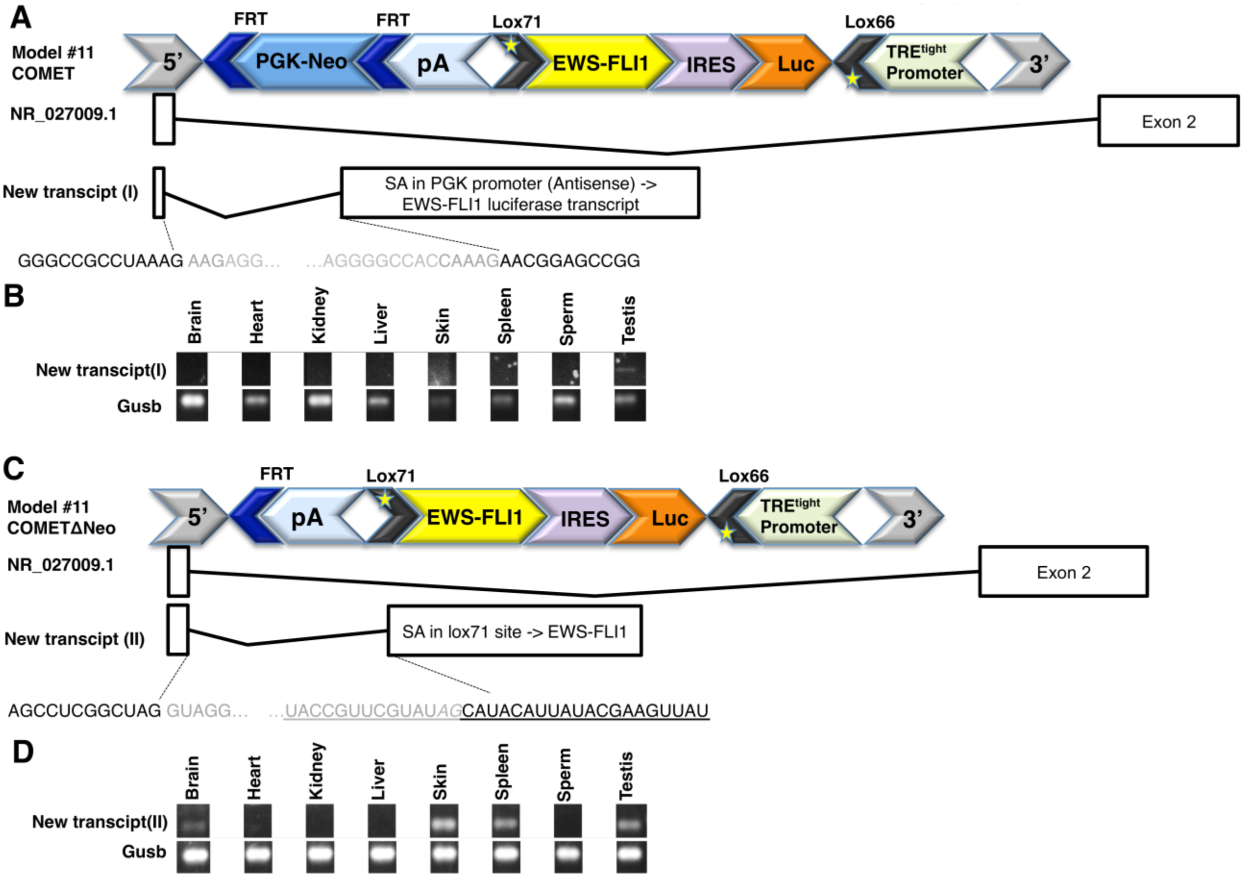

Supplementary Figure S18: (Model #10$^{\text{COMET and COMET}\Delta\text{Neo}}$): Alternative splicing identified in Model #10$^{\text{COMET and COMET}\Delta\text{Neo}}$ chimeric mice.

(A) The *Gt(ROSA)26Sor* locus containing the Model #10^{COMET} construct is shown. Below, the 2 exons of RNA-gene trap *ROSA 26* transcript variant 2 (NR_027009.1) are displayed. A new transcript (I) between the first exon of the RNA-gene trap *ROSA 26* transcript variant 2 and a splice acceptor (SA) located in the PGK promoter of the COMET cassette is highlighted. Underneath, nucleotides in black are part of the mature transcript, and nucleotides in gray are spliced out. **(B)** The new transcript (I) was only detected in the testis, as shown by RT-PCR on various organs. Gusb was used as housekeeping transcription control. **(C)** The same representation as in (A) without the PGK-Neo cassette, which was removed to generate the second-generation chimera (Model #10$^{\text{COMET}\Delta\text{Neo}}$). A new transcript (II) between the first exon of

the RNA-gene trap *ROSA 26* transcript variant 2 and a splice acceptor (SA) located in the *Lox71* site of the COMET cassette is highlighted. **(D)** The new transcript (II) was detected in the testis, spleen, skin and, to a lesser extent, in the brain, as shown by RT-PCR.

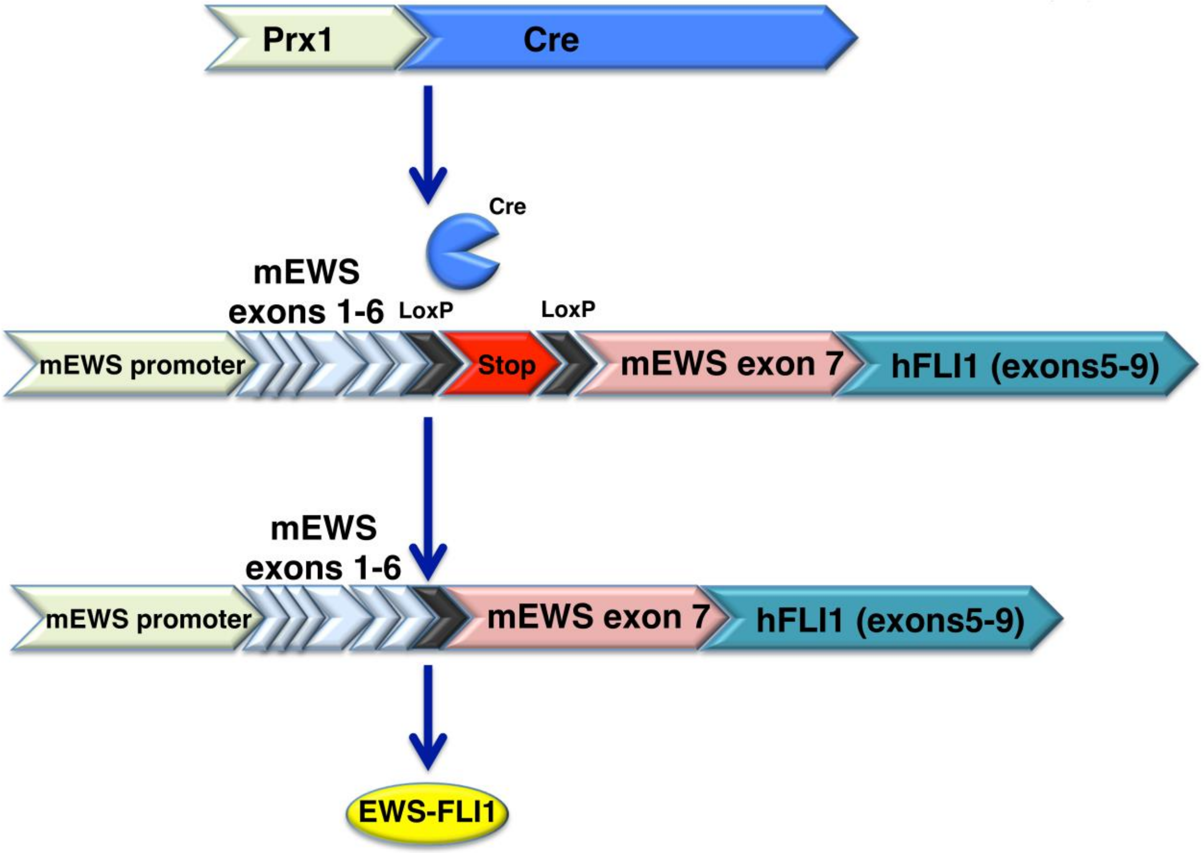

Protein expression from endogenous promoter in most mesenchymal tissues in limb bud

Supplementary Figure S19: (Model #11$^{Prx1Cre\text{-}EF}$): A schematic illustration of Model #11$^{Prx1Cre\text{-}EF}$.

EWS-FLI1 expression was designed to be driven by the endogenous *Ews* promoter in the mesenchymal tissue of the limb bud.

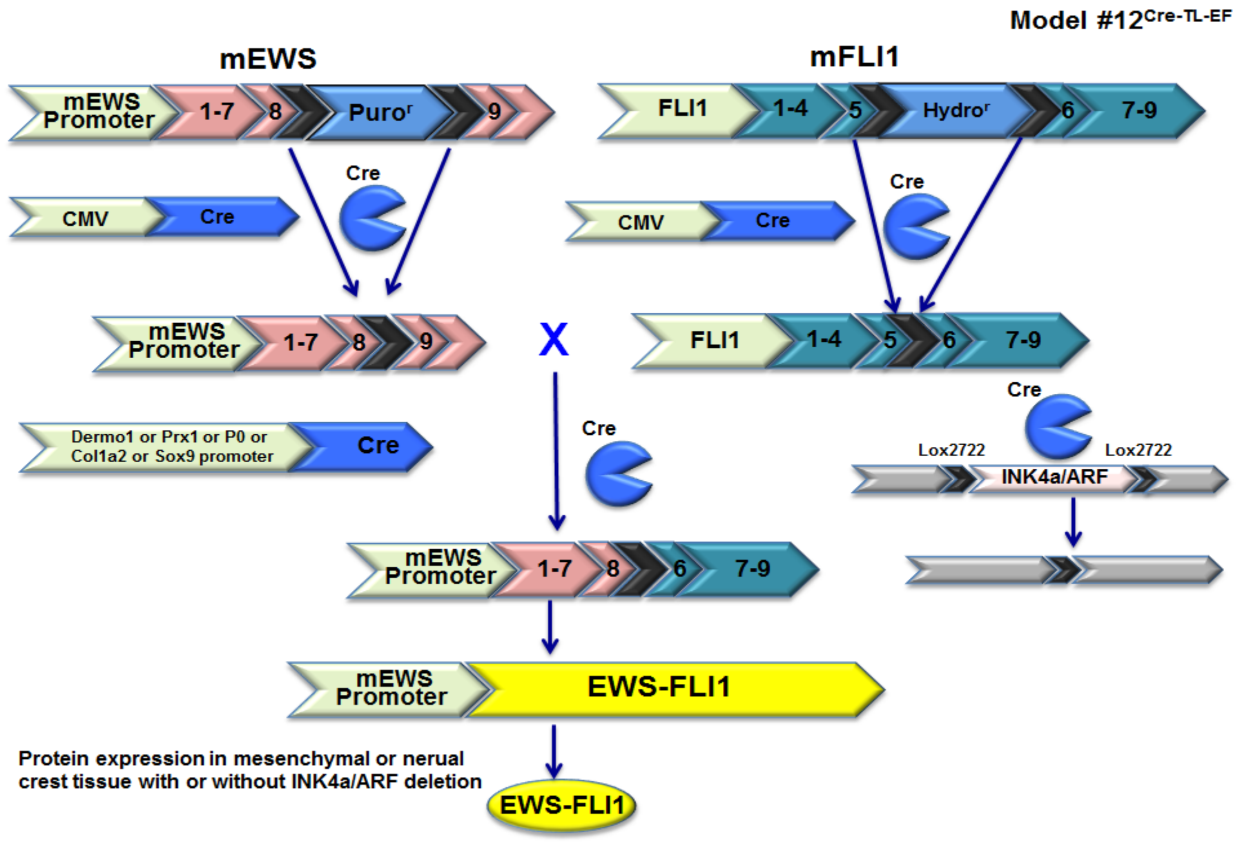

Supplementary Figure S20: (Model #12$^{Cre\text{-}TL\text{-}EF}$): A schematic illustration of the Model #12$^{Cre\text{-}TL\text{-}EF}$.

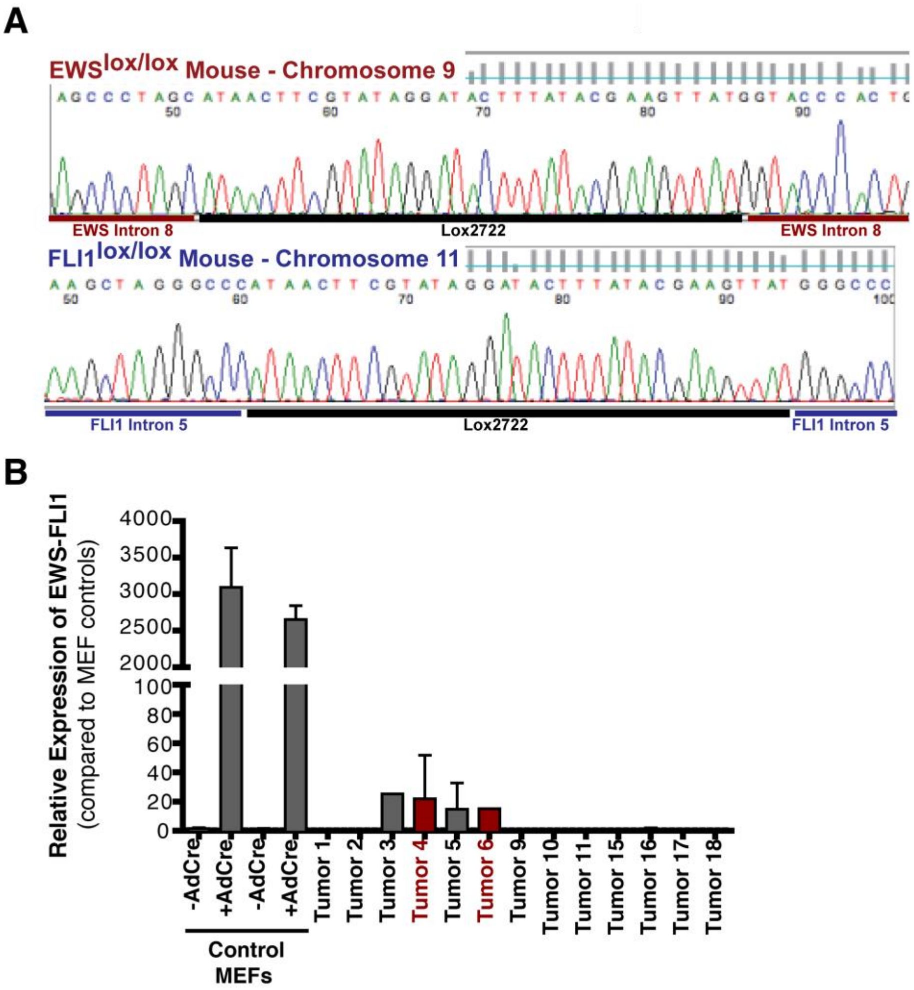

Supplementary Figure S21: (Model #12$^{Cre\text{-}TL\text{-}EF}$):

(A) Genomic DNA sequencing demonstrating loss of *Puro*^{r} and *Hydro*^{r} cassettes in the mouse *Ewsr1* and *Fli1* loci, respectively. Subsequently, single lox sites between exons 8 and 9 of *Ewsr1* and between exons 5 and 6 of *Fli1* were generated.

(B) RT-qPCR analysis of tumors isolated from the mice studied for mouse *Ews-Fli1* expression. Two tumors in red were obtained from control mice either lacking the *Fli1*^{lox} allele or *Cre*$^{-}$. MEFs expressing mouse *Ews-Fli1* were used as a control. *Hprt* was used as the control gene.

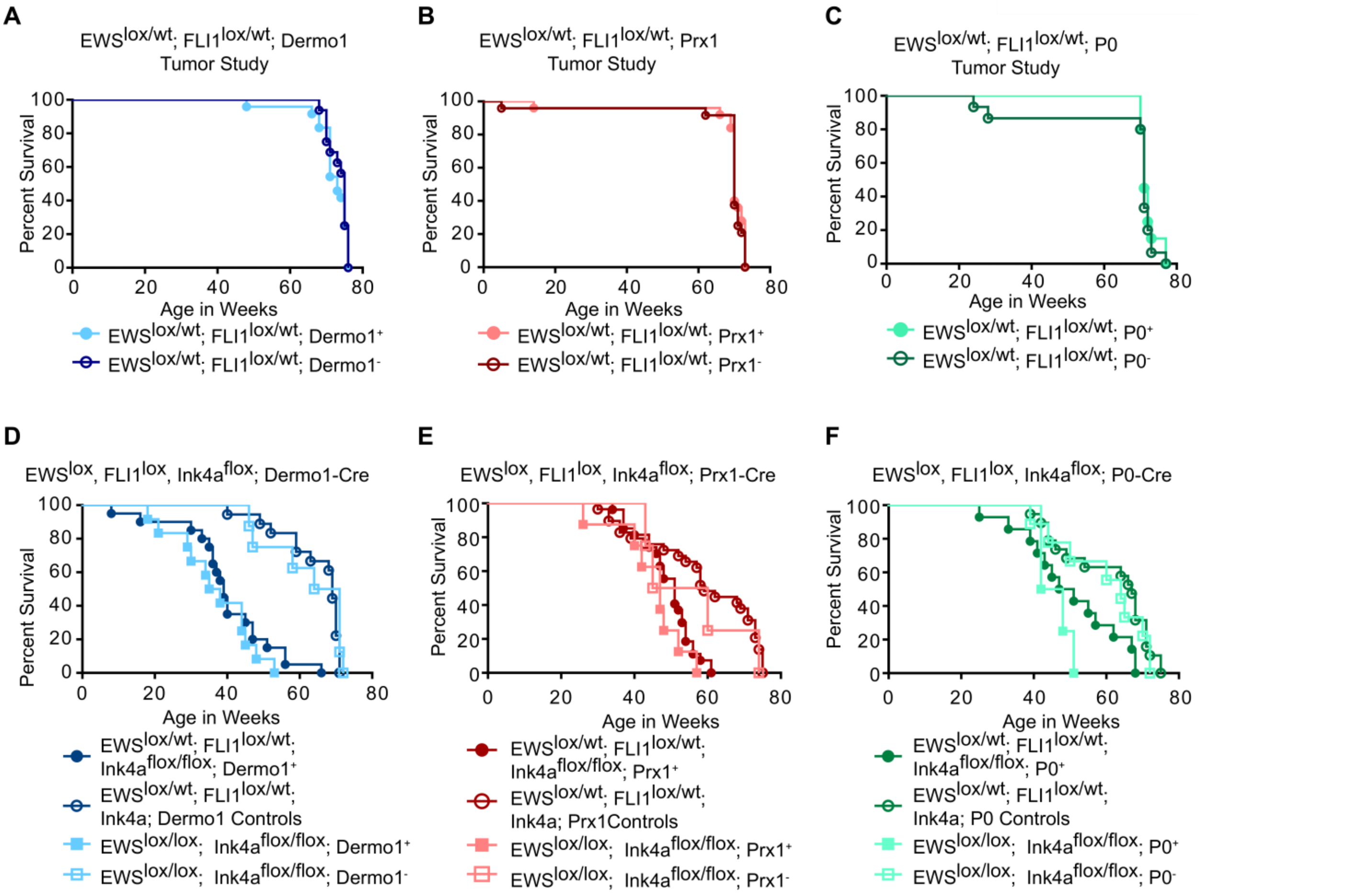

Supplementary Figure S22: (Model #12$^{Cre\text{-}TL\text{-}EF}$):

(A-C) Kaplan Meier curves for cohorts of *Ews*$^{lox/wt}$; *Fli1*$^{lox/wt}$; *Cre*$^{+}$ compared to *Ews*$^{lox/wt}$; *Fli1*$^{lox/wt}$; *Cre*$^{-}$ cohorts. **A.** *Dermo1-Cre*. **B.** *Prx1-Cre*. **C.** *P0-Cre*. **(D-F)** Kaplan Meier curves for cohorts of *Ews*$^{lox/wt}$; *Fli1*$^{lox/wt}$; *Ink4a/Arf*$^{flox/flox}$; *Cre*$^{+}$ compared to the control mice (*Ews*$^{lox/wt}$; *Fli1*$^{lox/wt}$; *Ink4a/Arf*$^{flox/flox}$; *Cre*$^{-}$, *Ews*$^{lox/wt}$; *Fli1*$^{lox/wt}$; *Ink4a/Arf*$^{flox/wt}$; *Cre*$^{+}$, and *Ews*$^{lox/wt}$; *Fli1*$^{lox/wt}$; *Ink4a/Arf*$^{flox/wt}$; *Cre*$^{-}$) as well as compared to INK4a control mice. *Ews*$^{lox/lox}$; *Ink4a/Arf*$^{flox/flox}$; *Cre*$^{+}$ compared to control mice (*Ews*$^{lox/lox}$; *Ink4a/Arf*$^{flox/flox}$; *Cre*$^{-}$ and *Ews*$^{lox/lox}$; *Ink4a/Arf*$^{flox/wt}$; *Cre*$^{+}$). **D.** *Dermo1-Cre*. **E.** *Prx1-Cre*. **F.** *P0-Cre*.

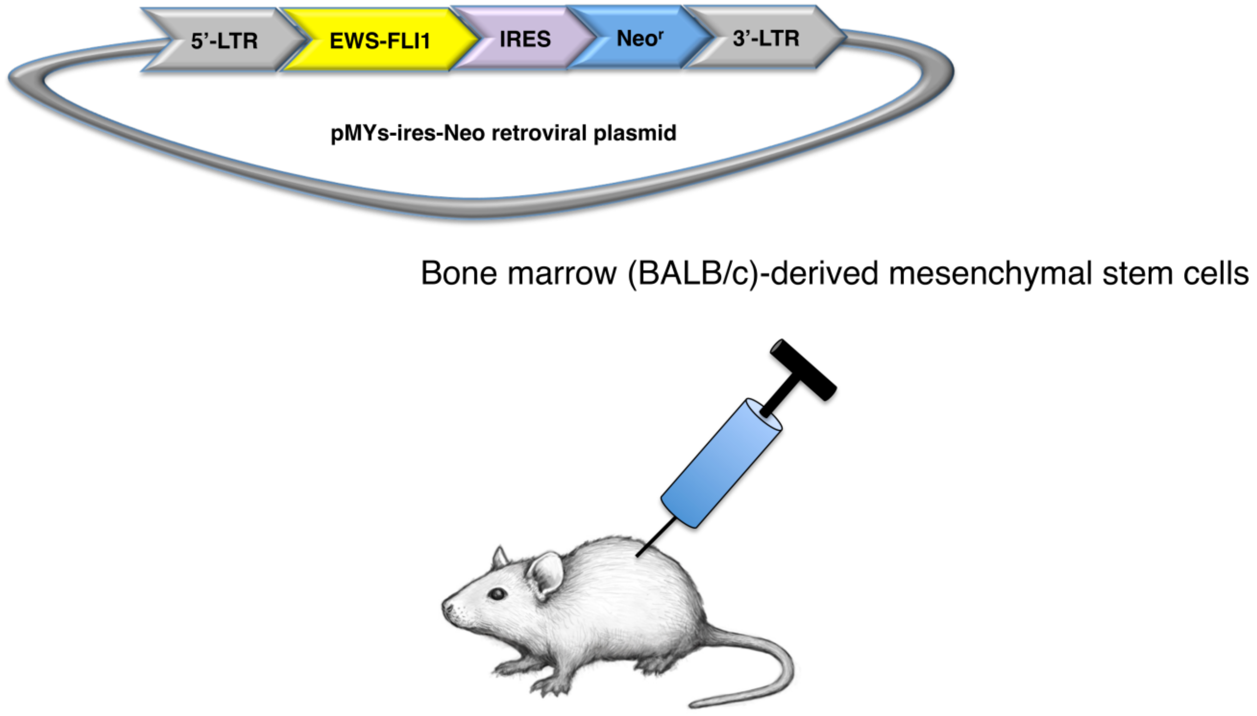

Supplementary Figure S23: (Model #13$^{\text{RetroLTR-EF}}$): The retroviral construct pMYs-EWS-FLI1-IRES-neo is shown. The EWS-FLI1 coding sequence was cloned into the pMYs-ires-Neo retroviral plasmid. Bone marrow (BALB/c)-derived MSCs were transduced with pMYs-EWS-FLI1 *in vitro* and were subcutaneously or intravenously injected into syngeneic mice.

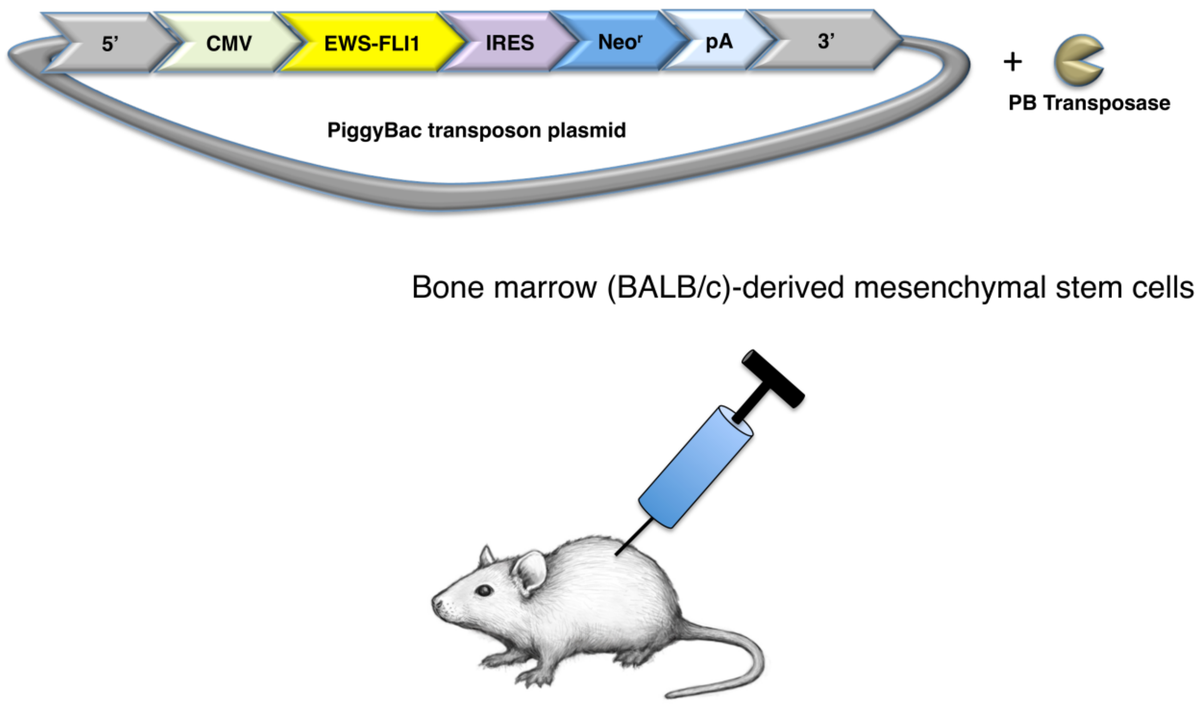

Supplementary Figure S24: (Model #14$^{piggyBac\text{-}EF}$): The transposon construct piggyBac-EWS-FLI1 is illustrated. The *EWS-FLI1* coding sequence was cloned into the piggyBac transposon plasmid. The plasmid was co-introduced with piggyBac transposase mRNA into bone marrow (BALB/c)-derived MSCs *in vitro*, and the cells were injected intravenously into irradiated syngeneic mice.

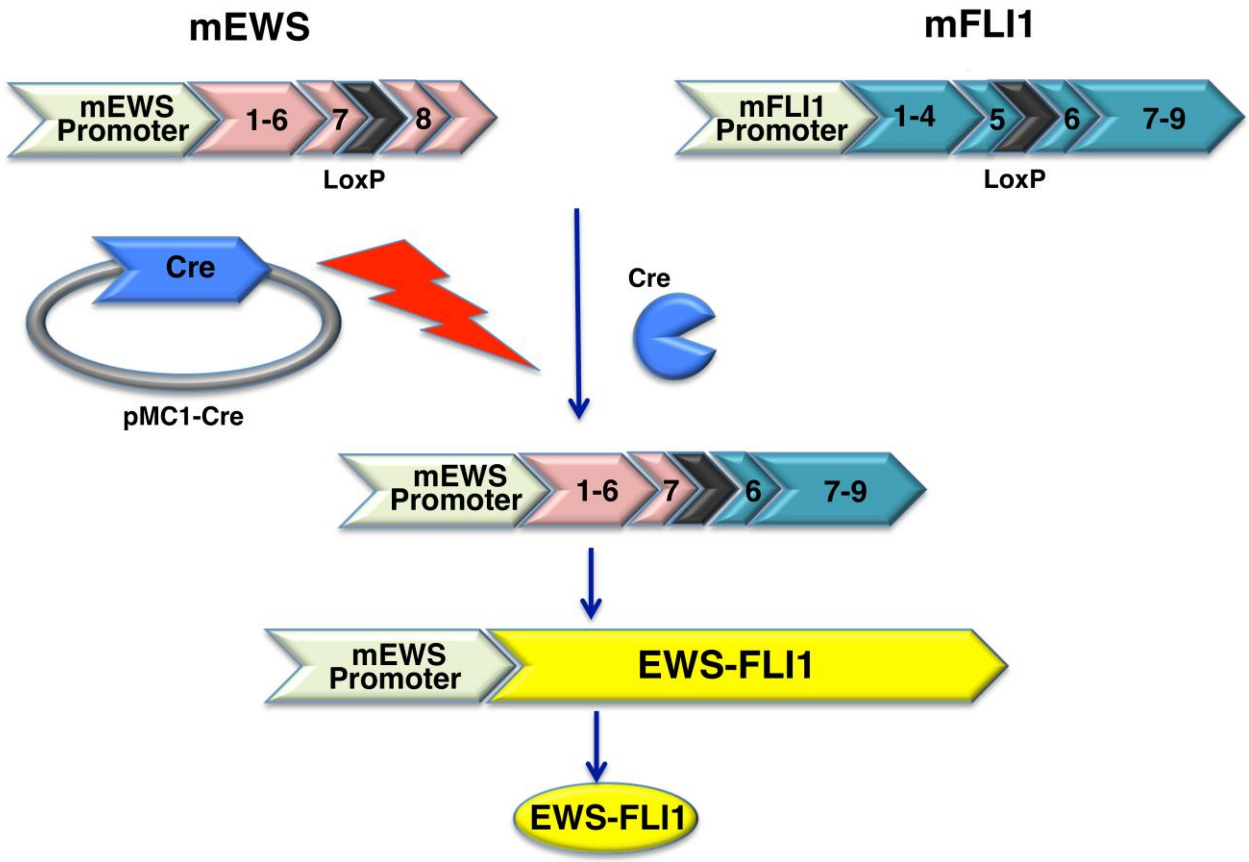

Local protein expression in muscle and surrounding tissues following in vivo electroporation.

Supplementary Figure S25: (Model #$15^{\text{CreEP-TL-EF}}$): A schematic illustration of Model #$15^{\text{CreEP-TL-EF}}$.

The *Cre* expression plasmid was delivered via electroporation into the muscle in *Ewsr1*$^{fl/+}$:*Fli1*$^{fl/+}$ mice *in vivo*, inducing somatic chromosomal translocation between *Ewsr1* intron 6 and *Fli1* intron 5.

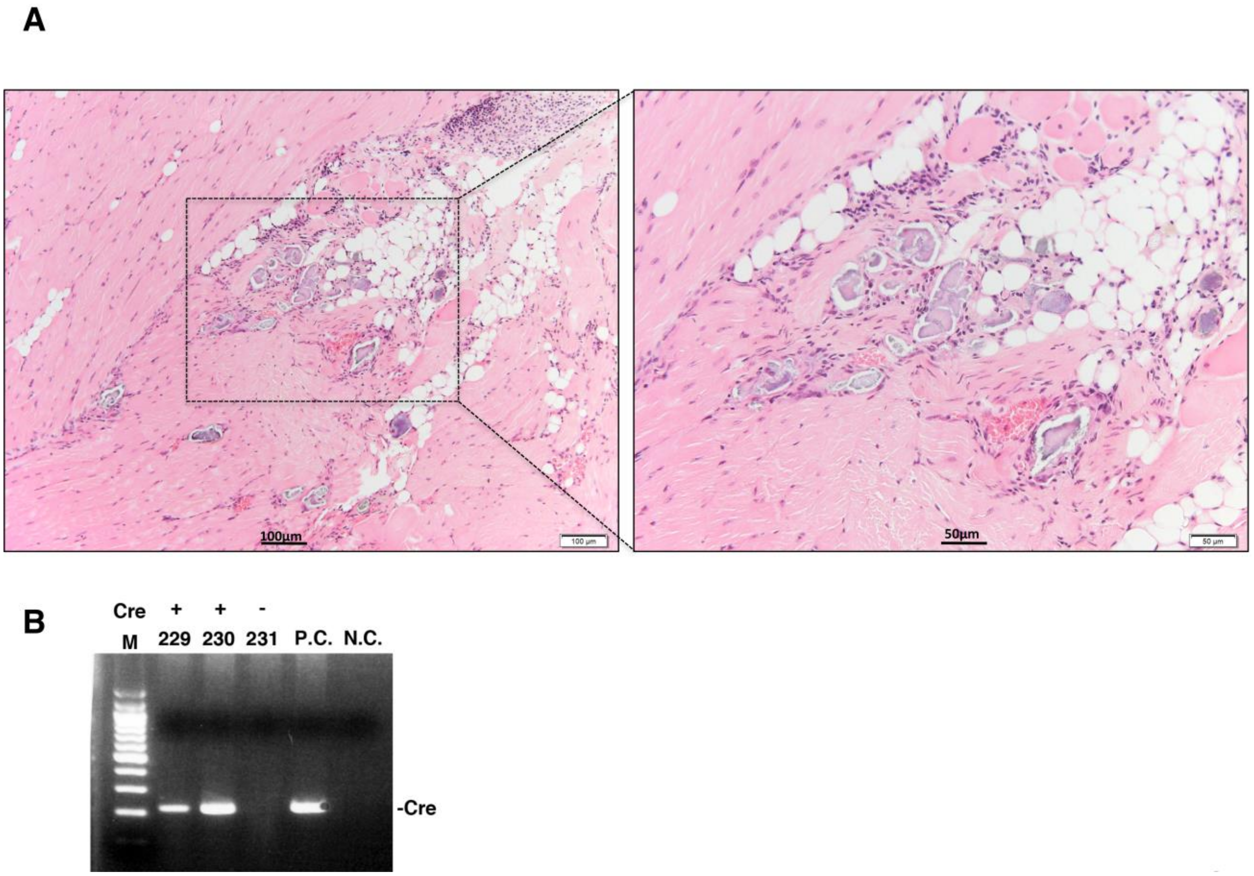

Supplementary Figure S26: (Model #15$^{\text{CreEP-TL-EF}}$): Muscular degeneration in a Model #15$^{\text{CreEP-TL-EF}}$ mouse.

(A) Disorganized muscular bundles are evident (left) in the electroporation site. Higher magnification revealed necrosis of the muscle fibers and the infiltration of inflammatory leukocytes, with accompanying adipocytes (right). **(B)** The expression of *Cre* recombinase at the regions of intra-muscular electroporation was confirmed by RT-PCR. The *Cre* expression plasmid was introduced into samples #229 and #230 but not into #231. The muscular tissue of the CAG-Cre transgenic mouse was used as a positive control (P.C.).

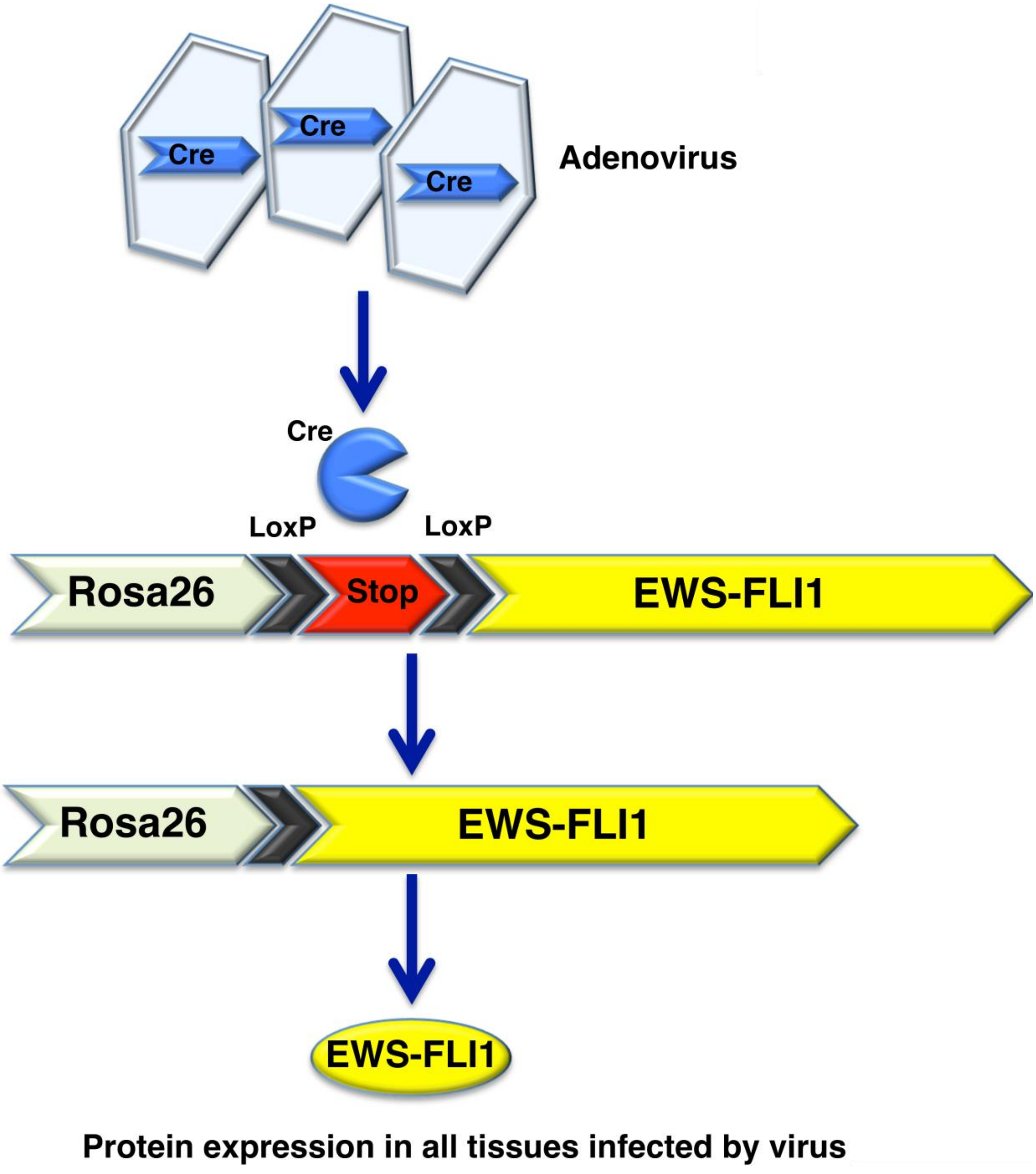

Supplementary Figure S27: (Model #16$^{Ad5Cre\text{-}EF}$): Schematic demonstrating the anticipated active *EWS-FLI1* allele produced upon Cre-mediated recombination between the loxP sites and the subsequent removal of the STOP-cassette in cells infected with adenovirus-Cre.

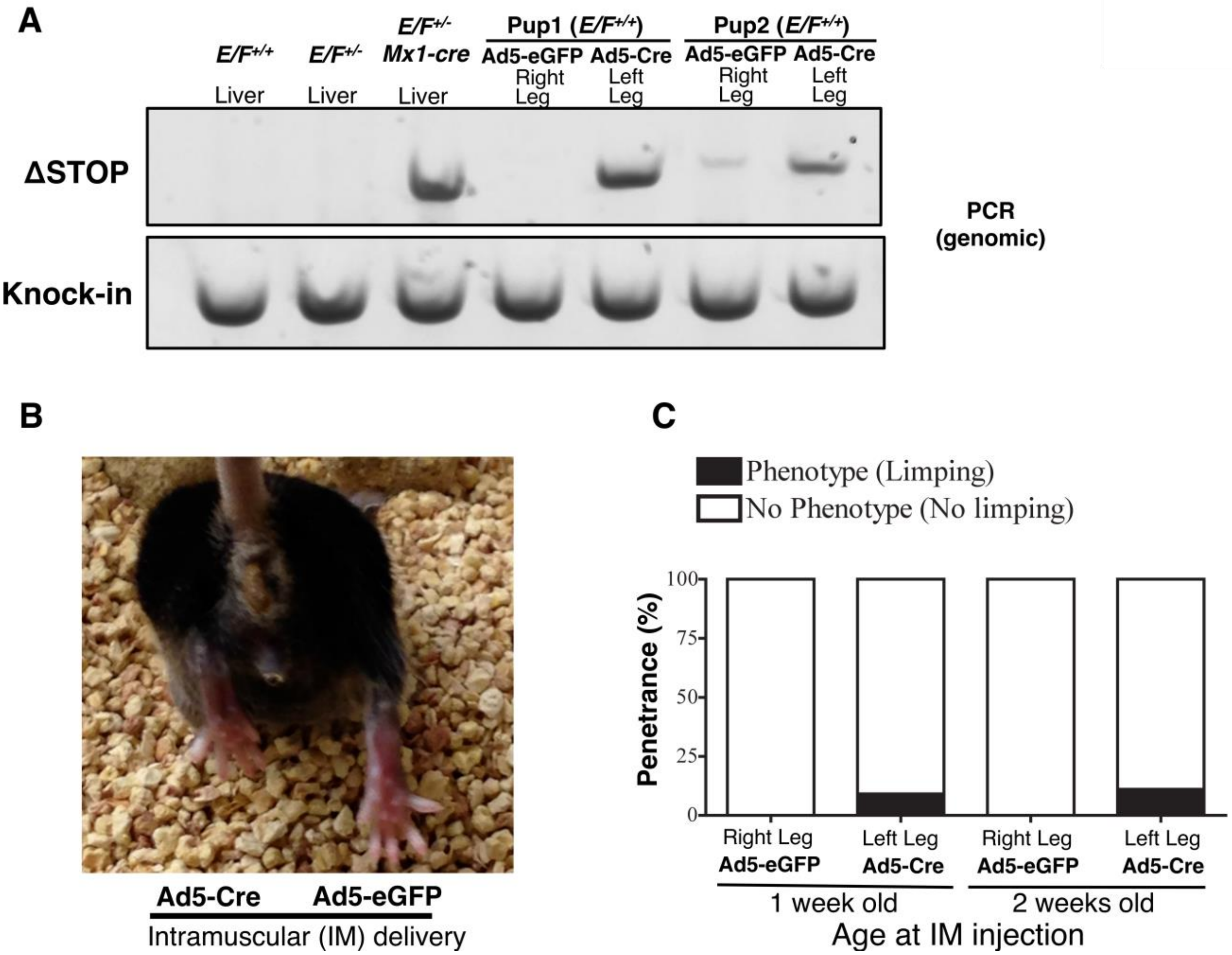

Supplementary Figure S28: (Model $\#16^{Ad5Cre\text{-}EF}$):

(A) Genomic DNA PCR confirming excision of the STOP-cassette in the left leg of 3-day-old *E/F*$^{+/+}$ pups 48h after IM injection with Ad5-Cre. The liver of leukemic *E/F*$^{+/-}$ *Mx1-cre*$^{+}$ mouse was used as a positive control for Cre-mediated recombination of the STOP-cassette. **(B)** Representative image showing prolonged clasping of the hind leg injected with Ad5-Cre, but not Ad5-eGFP, in a tail suspension test. **(C)** Variability in the penetrance of the limping phenotype observed in mice injected with Ad5-Cre in the left leg at 1 week (n=11) or 2 weeks (n=9) of age.

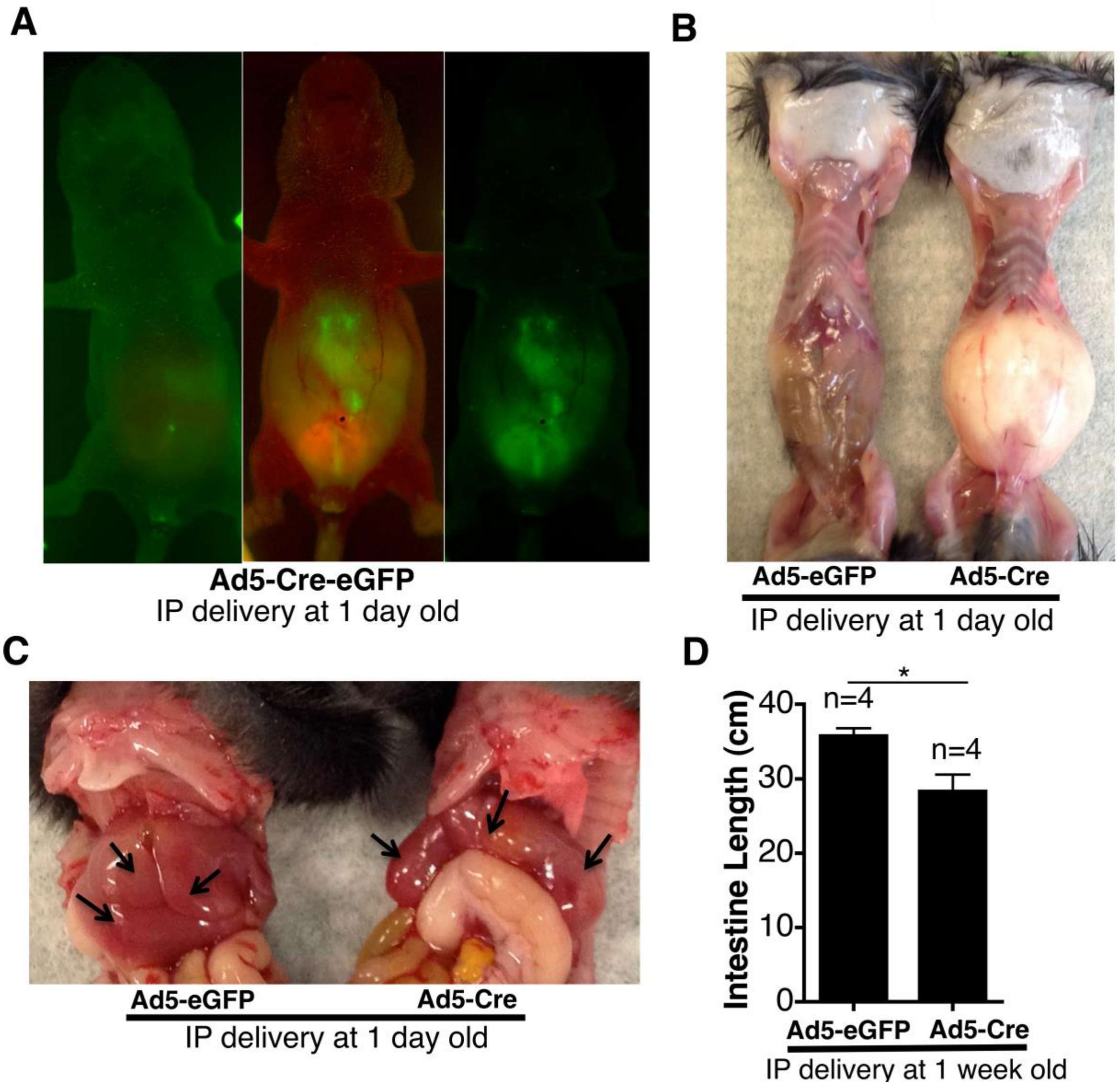

Supplementary Figure S29: (Model #16$^{Ad5Cre\text{-}EF}$):

(A) *Cre-eGFP* expression in 3-day-old $E/F^{+/+}$mice 48 h after IP injection with Ad5-Cre-eGFP. The Maestro whole-body imaging system was used to separate the autofluorescent background (left) from a true green fluorescent signal (middle and right). **(B)** Representative image showing the abdominal distention phenotype observed in mice IP-injected with Ad5-Cre vs. littermate controls of the same genotype injected with Ad5-eGFP. **(C)** An image showing the malformed liver observed in Ad5-Cre IP-injected mice but not in Ad5-eGFP IP-injected mice. **(D)** Intestine length of $E/F^{+/+}$ mice IP-injected with Ad5-eGFP vs. Ad5-Cre at an age of 1 week.

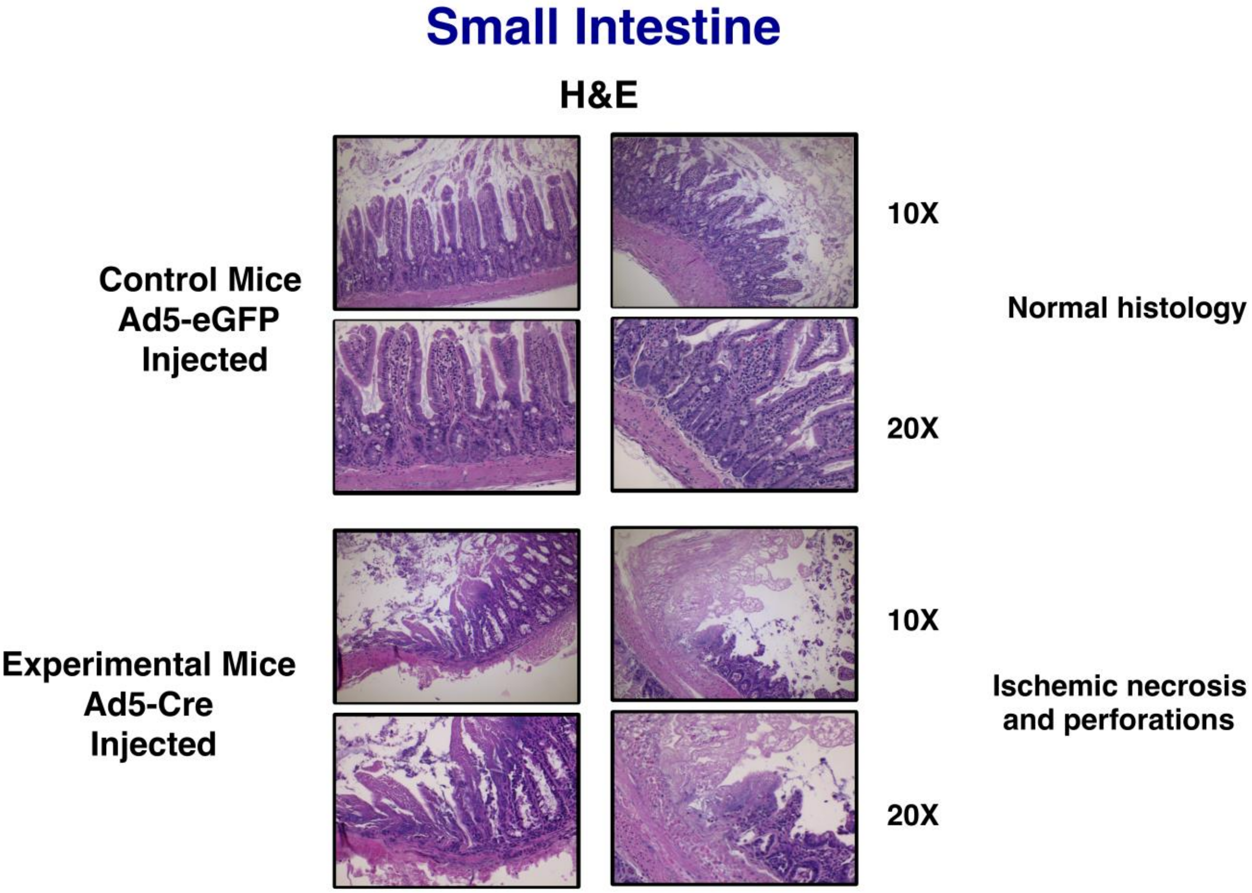

Supplementary Figure S30: (Model #16$^{Ad5Cre-EF}$):

Histopathological analysis of intestines from mice IP-injected with Ad5-eGFP or Ad5-Cre at 1 week of age. The intestine of Ad5-eGFP-injected mice showed normal histology, while ischemic necrosis and perforation was observed in the intestines of Ad5-Cre-injected mice.

Table S1: (Model #1$^{Runx2Cre\text{-}EF}$): Overview of mouse colonies generated with three different *Runx2-Cre* lines and loss of INK4A proteins.

	**Offspring**	**EWS-FLI1 Expression?**	**Phenotype**
#784 *E/F*$^{+/-}$ *Runx2-Cre*$^{+}$	viable	no	none
#1634 *E/F*$^{+/-}$ *Runx2-Cre*$^{+}$	viable	no	none
#777 *E/F*$^{+/-}$ *Runx2-Cre*$^{+}$	Embryonic lethal (before E13.5)	n.a.	Early embryonic death
#784 *E/F*$^{+/-}$ *Runx2-Cre*$^{+}$ *Ink4a*$^{-/-}$	viable	no	No shift in tumor spectrum compared to *Ink4a*$^{-/-}$ mice
#1634 *E/F*$^{+/-}$ *Runx2-Cre*$^{+}$ *Ink4a*$^{-/-}$	viable	no	No shift in tumor spectrum compared to *Ink4a*$^{-/-}$ mice

Table S2: (Models #4-9): Overview of transgenic mouse models #4 through #9.

**Models**	**Method**	**Number of F0**	**Transgenic F0**	**% of Transgenic mice**	**Remarks**
#4 $^{Cosco-EF}$	EmSC	44	0	0%	Embryonic lethal
#5 $^{Pgk-EF}$	µInject	16	2	12.5 %	No transgenic F1 from the 2 positive F0. (one female F0 was sterile)
#6 $^{Nse-EF}$	EmSC	32	1	3.1%	No transgenic F1 from the positive F0
#6 $^{Nse-EF-SV}$	EmSC	32	0	0%	Embryonic lethal
#7 $^{NEFL-EF}$	µInject	32	10	31.3%	4 transgenic lines were obtained (Mendelian inheritance). EWS-FLI1 expression in brain but no tumor development.
#8 $^{MT-EF}$	µInject	6	4	66%	4 transgenic lines were obtained (Mendelian inheritance) but EWS-FLI1 was not observed in tissue upon $ZnCl_2$ administration.
#9 $^{PLAPtTA-EF}$	µInject	2	2	100%	Two pUHD-10.3-EF stable lines were obtaind but back-cross with the tTA expressing strain (LT1) failed.

The transgenic mouse models were either generated by microinjection of the transgene (µinject) or through the generation of stable embryonic stem cell (EmSC) lines. The number of resulting F0 mice is listed in the third column with the transgenic F0 penetrance indicated in the fourth and fifth columns. The last column indicates the phenotype(s)/outcome(s) of each model.

Table S3: (Model #10$^{\text{COMET and COMET}\Delta\text{Neo}}$): Overview of chimeric mice generation.

**Chimera**	**Clone (from)**	**Chimera (% coat)**	**Agouti F1 (+)**
COMET	2D8	1 (90%)	
	2D8	2 (80%)	11 (0)
	2D8	3 (80%)	
	2D8	4 (70%)	3 (0)
	2D8	5 (50%)	
	2C12	6 (100%)	17 (0)
	2C12	7 (100%)	
COMET$^{\Delta\text{Neo}}$	1C3 (2D8)	8 (60%)	11 (0)
	1C3 (2D8)	9 (70%)	
	1C3 (2D8)	10 (85%)	22 (0)
	1C3 (2D8)	11 (80%)	
	1C3 (2D8)	12 (80%)	
	1C3 (2D8)	13 (85%)	
	2A3 (2C12)	14 (75%)	
Total			64 (0)

Chimeric mice were either generated from COMET or COMET$^{\Delta\text{Neo}}$ embryonic stem cell clones. Names of clones are indicated in the second column; 1C3 and 2A3 are respectively subclones of 2D8 and 2C12. The resulting chimeric mice are listed in the third column; the chimerism is determined by the percentage of agouti coat for each mouse. The number of agouti F1 obtained by backcrossing of chimera with C56BL/6 is indicated in the last column (no positive F1 carrying the COMET or COMET$^{\Delta\text{Neo}}$ construct is detected); black F1 are omitted.

Table S4: (Model #12$^{Cre\text{-}TL\text{-}EF}$):

	Genotype	Number of Mice	Sarcoma	Liver, Kidney & Spleen	Other	Unknown	68+ Weeks
1.	$EWS^{lox/wt}$; $FLI1^{lox/wt}$; $Ink4a/ARF^{flox/flox}$; Sox9+	21	4	2	6	8	1
	Controls	20	2	1	1	6	10
2.	$EWS^{lox/wt}$; $FLI1^{lox/wt}$; $Ink4a/ARF^{flox/flox}$; Prx1+	27	2	13	3	9	0
	Controls	29	0	3	1	13	12
3.	$EWS^{lox/wt}$; $FLI1^{lox/wt}$; $Ink4a/ARF^{flox/flox}$; Dermo1+	20	3	5	3	8	0
	Controls	18	2	3	0	3	10
4.	$EWS^{lox/wt}$; $FLI1^{lox/wt}$; $Ink4a/ARF^{flox/flox}$; P0+	14	1	8	1	2	2
	Controls	19	0	1	2	7	9

Macroscopic phenotype of the mice in the $EWS^{lox/wt}$; $FLI1^{lox/wt}$; $Ink4a/Arf^{flox/flox}$; Cre^{+} compared to the control mice ($EWS^{lox/wt}$; $FLI1^{lox/wt}$; $Ink4a/Arf^{flox/flox}$; Cre^{-}, $EWS^{lox/wt}$; $FLI1^{lox/wt}$; $Ink4a/Arf^{flox/wt}$; Cre^{+} and $EWS^{lox/wt}$; $FLI1^{lox/wt}$; $Ink4a/Arf^{flox/wt}$; Cre^{-}). Liver, Kidney and Spleen column refers to obvious abnormalities with these organs, usually enlargement and discolorment. Other refers to issues other than sarcomas or liver/kidney/spleen issues. Unknown are mice that were either found dead in the cage or nothing abnormal under necropsy. 68+ weeks are mice that were euthanized at the end of the study.
